# Outcome Reporting in Clinical Trials on Kangaroo Mother Care in Newborns: A Systematic Review for the Development of Core Outcome Set

**DOI:** 10.1155/jonm/5662163

**Published:** 2025-10-09

**Authors:** Ying Xin Li, Yan Ling Hu, Han Mei Peng, Yuan Li, Xia Li, Wen Qian Su, Xiao Wen Li, Qiong Chen, Xing Li Wan

**Affiliations:** ^1^Department of Neonatology Nursing, West China Second University Hospital, Sichuan University, Chengdu, Sichuan, China; ^2^Key Laboratory of Birth Defects and Related Diseases of Women and Children, Ministry of Education, Chengdu, Sichuan, China; ^3^Department of Nursing, West China Second University Hospital, Sichuan University, Chengdu, Sichuan, China

**Keywords:** core outcome set, evidence-based medicine, infant, kangaroo-mother care method, newborn, systematic review

## Abstract

**Background:**

Kangaroo mother care (KMC) reduces mortality and morbidity in newborns, particularly benefiting preterm infants. However, substantial heterogeneity in the selection, measurement, and reporting of outcomes across KMC trials hinders evidence synthesis and clinical application, underscoring the urgent need for standardized core outcomes.

**Objective:**

To identify outcomes used to evaluate KMC as the foundational step toward developing a core outcome set.

**Methods:**

We followed the Preferred Reporting Items for Systematic Reviews and Meta-Analyses (PRISMA) statement for reporting. A comprehensive search of MEDLINE, Cochrane Library, Embase, CINAHL, CNKI, SinoMed, WanFang Data, and clinical trial registries was conducted for studies published or registered from January 1, 2014 to July 7, 2024. Risk of bias in the studies was not assessed, as the review did not focus on efficacy. Data on literature and outcomes were extracted for narrative synthesis. Outcomes were categorized using the COMET outcome taxonomy.

**Results:**

A total of 5730 studies were screened, and 292 studies were included in this review, consisting of 162 articles and 130 protocols, with a total of 112,601 participants. These studies were conducted across 40 countries, with the majority from lower-middle-income countries and upper-middle-income countries. A total of 1306 outcomes were extracted, resulting in 142 unique outcomes, which spanned 5 core areas and 29 outcome domains. The most frequently reported core area was physiological/clinical (256 studies, 87.67%), followed by the life impact (116 studies, 39.73%). In contrast, the death core area received limited attention, with only 10.96% of studies reporting mortality or survival outcomes. The most commonly reported outcome domain was general outcomes (185 studies, 63.36%), followed by pregnancy, puerperium, and perinatal outcomes (84 studies, 28.77%). The three most reported outcomes were breastfeeding outcomes (84 studies, 28.77%), weight/length/head circumference/(82 studies, 28.08%), and pain (67 studies, 22.95%).

**Conclusions:**

Current research on outcome reporting for KMC is comprehensive, covering most outcome domain across the five core areas. However, significant heterogeneity in outcome definitions and reporting exists across studies. Therefore, the development of a core outcome set for KMC is critical to reduce this heterogeneity, facilitate comparisons between studies, and enhance the integration of results into clinical practice and future research.

## 1. Background

Global newborn health faces significant challenges, with 2.3 million neonates dying in 2022, amounting to 47% of all child deaths under the age of five years [1]. Complications arising from preterm birth continue to be the main cause of death among children under the age of five, with a mortality of 6.31‰, representing 17.7% of all childhood deaths [2, 3]. Therefore, reducing newborn mortality and improving their clinical outcomes, particularly for vulnerable groups like preterm infants, remain critical public health issues that require urgent attention.

Kangaroo mother care (KMC) entails continuous skin-to-skin contact between infants and their mothers, with breastfeeding whenever possible [4, 5]. KMC primarily developed for preterm and low-birth-weight infants, significantly reduces mortality and morbidity in this population [6]. Additionally, KMC is increasingly applied to term or near-term newborns in diverse clinical settings to reduce pain, enhance parent–infant bonding, promote breastfeeding, and improve thermoregulation [7–9]. To capture the full spectrum of outcomes and assess its broader applicability, this review includes studies involving all newborns, with a primary focus on preterm infants. However, the comparison of KMC studies is challenging due to significant heterogeneity in the selection, measurement, and reporting of outcomes, which limits the ability to conduct systematic reviews and meta-analyses and prevents the provision of higher-level evidence to support KMC practices [10]. This diminishes the value of KMC research, resulting in resource waste and raising concerns of potential selective reporting bias. Systematic reviews highlight the considerable heterogeneity in KMC studies, which complicates comparisons and underscores the need for standardized outcomes and measurements in future [11, 12].

Currently, there is a lack of consensus on how to assess the effectiveness of KMC, and no unified standard exists for outcomes in clinical practice. To address this critical issue, the core outcome measures in effectiveness trials (COMET) initiative advocates for the development of a core outcome set (COS). It is the minimum set of outcomes that must be measured and reported in clinical studies [13]. Furthermore, the COMET initiative has also recommended a comprehensive outcome taxonomy (a 38-category scale by Dodd et al.) that provides a standardized framework for outcome classification [14]. This taxonomy identifies five core areas: death, physiological/clinical, life impact, resource use, and adverse events, which can help to standardize outcome reporting [14]. This taxonomy is endorsed by the COMET initiative and Cochrane for classification [15, 16]. Adopting a COS alongside this standardized taxonomy improves the applicability of outcomes, reduces heterogeneity across various clinical trials, enhances the integration and comparison of related studies, and minimizes reporting bias [14].

Significant progress has been made in developing COS across many medical fields, offering effective solutions to address research heterogeneity. For example, in neonatal research, a COS has been developed for high-income countries (HICs), encompassing 12 core outcomes, including survival, sepsis, necrotizing enterocolitis (NEC), radiological brain injury, general gross motor, and cognitive abilities [17]. Another COS focused on family-centered care is currently under development, aiming to evaluate such practices in neonatal care [18]. Additionally, COS have been developed for specific conditions such as NEC and intrauterine growth restriction [19, 20]. These COS have standardized outcome reporting, facilitated evidence synthesis, and provided a solid foundation for the development of clinical practice guidelines.

However, these existing COS have not addressed KMC specifically. There is a critical need for a COS that is tailored to the unique characteristics and complexities of KMC. The definition and content of existing COS differ fundamentally from the nature of KMC, making them unsuitable for evaluating KMC practices. Consequently, the challenge of standardizing outcomes in KMC research persists, and a dedicated COS for KMC is urgently needed.

Thus, developing a COS for KMC is crucial to enhance the consistency and comparability of KMC trials. This study represents the first phase of the process to develop a COS for KMC [10]. The primary aim is to evaluate the current state of outcome reporting in KMC clinical trials and to lay the foundation for the development of a COS for KMC.

## 2. Methods

This study was registered with the international prospective register of systematic reviews (PROSPERO ID: CRD 42024611716). The protocol for the COS was also published in BMJ Open [10] and followed the methodology recommended by the COMET initiative [13]. It is reported in accordance with the PRISMA statement [21] and the PRISMA 2020 checklist as shown in supporting information (available here).

### 2.1. Search Strategy

In brief, we conducted a comprehensive search, including MEDLINE, Cochrane Library, Embase, CINAHL, CNKI, SinoMed, and WanFang Data. Additionally, we searched for ongoing trials in three platforms: the International Clinical Trials Registry Platform, ClinicalTrials.gov, and the Chinese Clinical Trial Registry [22–24]. A supplementary search was also performed on Google scholar. Due to the large of research in KMC, the search covered publications from January 1, 2014 to July 7, 2024, and was limited to articles published in English and Chinese. Detailed search strategies are shown in Supporting Table S1.

### 2.2. Eligibility Criteria

The inclusion criteria were as follows. Population: only studies involving neonates were eligible. Intervention: interventions that include any form of KMC were considered. Comparison: Studies comparing KMC with standard care, routine care, placebo, sucrose solutions, any other interventions or no intervention were included. Outcome: all outcomes reported in the eligible studies were considered for evaluation. Design: interventional trials, including randomized controlled trials (RCT) and non-RCT studies.

The exclusion criteria were studies where the full text was unavailable, studies that did not report any outcomes, and the design type is conference abstracts, or comments, or systematic reviews or meta-analyses, or observational studies, or reviews, or letters, or case reports.

### 2.3. Study Selection and Screening

We manage literature with EndNote 21.0. The initial screening process involved an independent evaluation of the titles and abstracts from the citations retrieved during the search. This step was performed separately by two reviewers (LYX, PHM). Subsequently, the full texts of potentially relevant studies were assessed to verify their eligibility. In cases of uncertainty regarding inclusion, a third reviewer (SWQ) was consulted to achieve consensus.

### 2.4. Quality Assessment

The objective of this review was to extract, analyze, and synthesize outcomes, as well as to calculate their frequency of use. Since the primary aim was to collect as broad a range of outcomes as possible, the quality of the studies was not assessed. The focus was not on evaluating treatment effects. So as with other systematic reviews conducted with the sole intent of developing COSs [25–28], we did not perform risk of bias assessment.

### 2.5. Data Extraction

Data extraction was using Microsoft Excel forms, which were pre-piloted prior to the start of the process. The forms captured essential details, including author(s), publication year, the country of origin, title, clinical characteristics of study participants (e.g., gestational age, birth weight), sample size, study design, type of KMC (intermittent, continuous, duration), comparator(s), outcomes, outcome definitions, timing of outcome assessed, measurement instruments, and methods.

After the data extraction process, cross-verification was performed to ensure accuracy. Any discrepancies were addressed through discussed and resolved by consensus, with a third author consulted when needed. Given the expected heterogeneity among the included studies, statistical data synthesis was not feasible and effect size was not extracted. The primary objective of this review was to provide a descriptive synthesis of the reported outcomes. Therefore, a narrative synthesis of outcomes was conducted.

### 2.6. Indexing of Outcomes and Domain Categorization

Outcomes were defined as all measurement terms used to specify clinical endpoints or physiological events in the included studies, covering all relevant domains. These outcomes were categorized based on the COMET outcome taxonomy [14]. Outcomes with similar or consistent definitions were independently consolidated into unique outcomes by two authors (LYX, HYL), and the process was reviewed by members of the research team (LYX, PHM, CQ). The frequency of each unique outcome, along with the total number of verbatim extracted outcomes, was recorded. The definition and explanation of the COMET outcome taxonomy are shown in Supporting Table S2.

All descriptive analyses and graphical presentations were performed using R (version 4.4.1).

## 3. Results

### 3.1. Search Results and Study Characteristics

**Figure 1:**
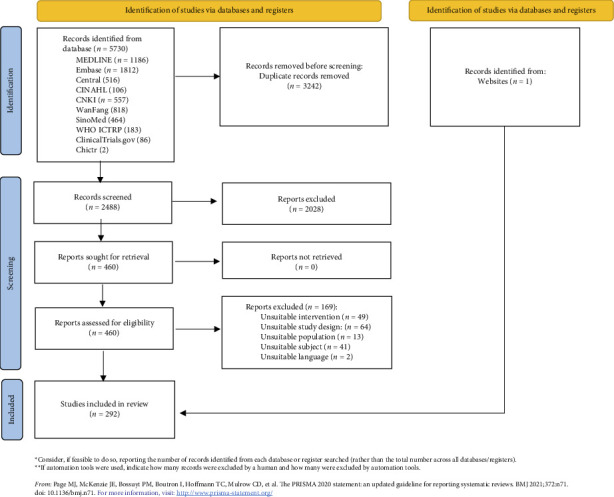
PRISMA flow diagram.

The search results and flow diagram are summarized in Figure 1. A total of 5730 studies were screened, and 292 studies were included in this review, consisting 162 articles and 130 protocols, with a total of 112,601 participants. The references of the included studies are provided in Supporting Table S3. The characteristics of included studies are summarized in Table 1, with further details available in Supporting Table S4. The studies originated from 40 countries, with 134 studies from lower-middle-income countries (LMICs), 100 studies from upper-middle-income countries (UMICs), 45 studies from HICs, and 7 studies from low-income countries (LICs). Additionally, 6 studies were conducted across multiple countries. Geographic distribution of the studies was as follows: Asia (179 studies, 61.30%), transcontinental countries (43 studies, 14.73%), Europe (24 studies, 8.22%), North America (19 studies, 6.51%), South America (10 studies, 3.42%), Africa (16 studies, 5.48%), and Oceania (1 study, 0.34%).

### 3.2. Identification of Outcomes

**Figure 2:**
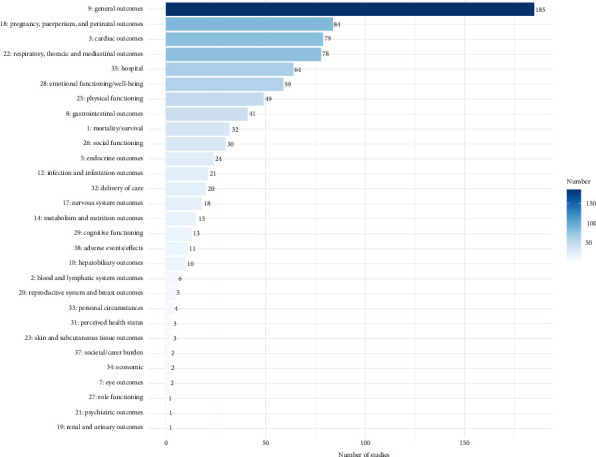
Outcome domain and study frequency.

**Figure 3:**
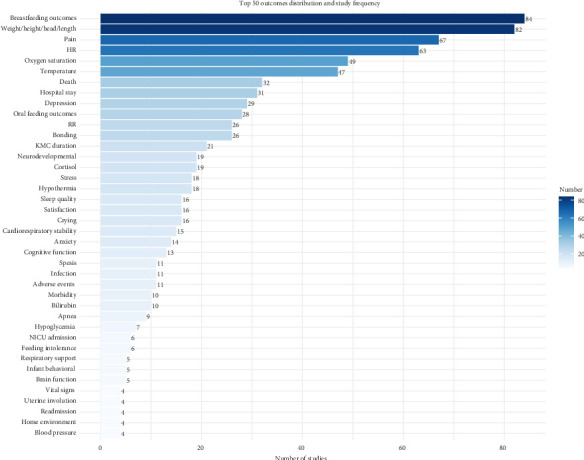
Top 50 outcomes' distribution and study frequency.

A total of 1306 outcomes were extracted from 292 studies. After deduplication and standardization, 142 unique outcomes were obtained, covering five core areas and 29 outcome domains, as shown in Table 2. The most frequently reported core area was physiological/clinical (256 studies, 87.67%), while the most commonly reported outcome domain was general outcomes (185 studies, 63.36%). This was followed by pregnancy, puerperium, and perinatal outcomes (84 studies, 28.77%), as illustrated in Figure 2. Among the top three reported outcomes, breastfeeding outcomes were most prevalent (84 studies, 28.77%), followed by weight/length/head circumference/(82 studies, 28.08%), and pain (67 studies, 22.95%). The top 50 outcomes are depicted in Figure 3, and the frequency distribution of all 142 unique outcomes can be found in Supporting Figure S1.

Evaluation of outcome definitions. Of the 1306 individual outcomes, 290 were accompanied by definitions, with relevant outcomes defined for each core area. Most of the definitions pertain to the physiological/clinical core area, relating to the clinical evaluation of KMC, such as breastfeeding outcomes, hypoglycemia, hypothermia occurrence, pain relief, weight, feeding intolerance, cardiorespiratory stability, infection, depression, and anxiety. Other definitions pertained to KMC practices, such as KMC duration and KMC cognitive aspects.

However, these 290 definitions were not entirely consistent and did not define clearly define the successful implementation of KMC. Definitions of adverse effects varied across studies and included symptoms such as nausea, vomiting, coughing, gagging, reflux, arterial oxygen saturation (SaO_2_) level below 80%, heart rates lower than 80 bpm or greater than 200 bpm, hypothermia, hypoglycemia, and physiological instability during skin-to-skin contact, as well as apnea and clinical instability. The definition of hypothermia also varied, with temperature thresholds such as < 36°C, axillary temperature below 32°C, or 36.5°C.

Additionally, the definition of mortality varied, including death from enrollment to 28 days of age, early infant deaths from enrollment to 180 days of age, and all neonatal deaths within 28 postnatal days.

### 3.3. Outcome Measures

In the 292 studies included, the frequency and classification of 142 unique outcomes are shown in Table 2 and Supporting Figure S1. Figure 2 displays the outcome domains ranked by frequency, while Figure 3 illustrates the distribution and study frequency of the top 50 outcomes.

#### 3.3.1. Death

Mortality was reported as an outcome in a relatively small proportion of the included studies, with only 32 studies (10.96%) documenting mortality or survival outcomes. The observation periods varied, ranging from 7 days to 20 years, with the most commonly reported outcome being neonatal mortality, specifically death within the first 28 days of life. The longest follow-up period was 20 years.

#### 3.3.2. Physiological/Clinical

In the physiological/clinical core area, the outcome domains most frequently reported were as follows: (1) general outcomes, reported in 185 studies (63.36%), with the most commonly reported outcomes being weight/length/head circumference/(82 studies, 28.08%), pain (67 studies, 22.95%), and temperature (47 studies, 16.10%), reflecting overall health or physiological outcomes not specific to any one organ/system. (2) Pregnancy, puerperium, and perinatal outcomes, reported in 84 studies (28.77%). The outcomes reported under this domain are primarily related to breastfeeding, highlighting the role of KMC in promoting breastfeeding. (3) Cardiac outcomes, reported in 79 studies (27.05%), focused mainly on heart rate, which was reported in 63 studies (21.58%). (4) Respiratory, thoracic, and mediastinal outcomes, reported in 78 studies (26.71%), primarily included oxygen saturation, reflecting KMC's stabilizing effect on the respiratory system. (5) Gastrointestinal outcomes were reported in 41 studies (14.04%), with oral feeding outcomes being the most common, featured in 28 studies (9.59%).

#### 3.3.3. Life Impact

A total of 116 studies (39.73%) reported outcomes in the life impact core area, which encompasses nine outcome domains, including physical functioning, social functioning, role functioning, emotional functioning/well-being, cognitive functioning, and delivery of care. The outcome domain most frequently reported was emotional functioning/well-being, with 59 studies (20.21%) reporting outcomes such as depression and stress, reflecting KMC in alleviating negative emotions. The second most reported outcome domain is physical functioning, with 49 studies (16.48%) reporting outcomes, the most common outcome being neurodevelopmental outcomes. Social functioning was the third most reported outcome domain, with 30 studies (10.27%) focusing on outcomes like bonding, which reflects KMC's role in fostering mother–infant (or parent–infant) attachment. Additionally, outcomes related to stress (18 studies, 6.16%), sleep quality (16 studies, 5.28%), and satisfaction (16 studies, 5.48%) were commonly reported. Notably, while most studies focused on parental stress, the stress experienced by newborns received less attention, with only 6 studies (2.05%) reporting on newborn stress.

#### 3.3.4. Resource Use

This core area includes four outcome domains: economic, hospital, need for further intervention, and social/carer burden. All of these, except the need for further intervention, were reported in the studies. The most frequently reported outcome domain was hospital, with 64 studies (21.92%) addressing outcomes in this area. The most commonly reported outcome in this outcome domain was hospital stay (31 studies, 10.62%), followed by KMC duration (21 studies, 7.19%) and NICU admission (6 studies, 2.05%). Additionally, two studies (0.68%) reported outcomes related to economic, specifically cost. These outcomes reflect the impact of KMC on hospital stay, readmission, and associated cost.

#### 3.3.5. Adverse Events

Adverse events were reported in 11 studies (3.77%), with terminology including side effects, adverse reactions, complications, and others. Most studies treated adverse events as composite outcomes, reporting conditions such as nausea, vomiting, coughing, gagging, reflux, SaO_2_ less than 80%, heart rate below 80 bpm or above 200 bpm, hypothermia, hypoglycemia, apnea, or physiological instability during KMC. A few studies also reported specific adverse reactions, such as rash and frequent falls.

## 4. Discussion

### 4.1. Interpretation of Findings

This systematic review included 292 studies, from which 1306 individual outcomes were extracted and synthesized into 142 unique outcomes. These unique outcomes were categorized into five core areas and further subdivided into 29 outcome domains. In contrast, existing systematic reviews or meta-analyses on KMC have primarily focus on outcomes within a single domain, without systematically and comprehensively analyzing the full spectrum of outcome related to KMC [29–43].

The physiological/clinical core area, comprising 256 studies (87.67%), was the most frequently reported, reflecting the significant impact of KMC on various physiological functions. The most commonly reported outcomes within this domain included breastfeeding, growth and development, procedural pain, and respiratory effects. This finding aligns with several systematic reviews that highlight KMC's influence on neonatal growth and development, breastfeeding outcomes, and improvements in vital signs [32, 34, 37, 38, 44–46]. The outcomes within this outcome domain are broad, covering multiple systems such as cardiovascular, respiratory, gastrointestinal, endocrine, and neurological. However, some outcomes are sparsely reported, with certain outcomes being mentioned only once. Furthermore, the assessment of procedural pain exhibited considerable heterogeneity. Pain was assessed using diverse tools, such as the Premature Infant Pain Profile (PIPP), Neonatal Infant Pain Scale (NIPS), Premature Infant Comfort Scale (PICS), etc. Additionally, measurement time points and pain definitions varied across studies. This heterogeneity complicates meta-analysis and direct comparisons.

The life impact core area received considerable attention, with 39.73% of studies reporting outcomes in this area, making it the second most frequently reported domain after physiological/clinical. Relevant outcomes in this area included neurodevelopmental outcomes, sleep quality, bonding, depression, stress, anxiety, cognitive function, and satisfaction. These outcomes reflect a shift towards patient- and family-centered research, emphasizing the emotional and psychological impacts on parents of neonates, particularly mothers and fathers, such as outcomes depression, stress, and anxiety. This indicates a transition in research focus, moving from predominantly physiological and pathological outcomes to a broader consideration of the emotional and psychological experiences of both infants and their families. This aligns with findings from a systematic review that underscored the emotional outcomes for parents of neonates [47]. However, there has been relatively less attention to the emotional experiences of the infants themselves, with only 6 studies (2.05%) assessing neonatal stress.

In contrast, the death core area received limited attention, with only 10.96% of studies reporting mortality/survival outcomes. This limited reporting could stem from several factors: (1) limitations in study design, such as short-term follow-up that might not capture the timing of death, or insufficient sample sizes limiting the statistical power to observe mortality rates; (2) challenges in data collection, especially in low-resource settings where hospital records might be incomplete or systematic mortality tracking is lacking; and (3) insufficient attention from researchers, who may prioritize short-term physiological outcomes. Nonetheless, from both the patient and clinical healthcare provider perspectives, mortality is one of the most critical outcomes and deserves greater emphasis. This is further supported by multiple systematic reviews on KMC, which highlight mortality as a key outcome, reinforcing the importance of this focus [31, 42, 48, 49]. To address this issue, we recommend (1) optimizing research designs, for example, extending follow-up periods and increasing sample sizes; (2) adopting standardized mortality recording tools and training data collection personnel to enhance quality control; and (3) raising researchers' awareness of mortality through avenues like academic conferences. Furthermore, including mortality in the COS for KMC to ensure all KMC studies report standardized mortality data could be a crucial step.

### 4.2. Outcome Heterogeneity and Its Impact

It is important to note that the significant heterogeneity in the outcome reporting and, crucially, a pervasive lack of standardized definitions across studies. This challenge aligns with the conclusions of several systematic reviews [11, 12, 50]. Specifically, the sporadic reporting of numerous outcomes presents a considerable methodological problem. For instance, outcomes such as anemia, asthma, disability, and children's problem behavior, among 56 outcomes, were each reported only once, as detailed in Table 2 and the Supporting Figure S1. Similarly, domains like role function, psychiatric outcomes, and renal and urinary outcomes were each documented in a single study. This fragmented reporting limits the ability to draw robust conclusions regarding KMC's impact on these specific aspects.

Furthermore, the absence of uniform outcome definitions and consistent reporting practices poses a significant challenge with several implications: (1) Impact on Effect Estimation and Reliability: Inconsistent measurement or definition of outcomes can lead to biased effect estimates in meta-analyses, thereby undermining the reliability and generalizability of research conclusions and contributing to inconclusive findings. For example, the definition of hypothermia varied, with temperature thresholds such as < 36°C, axillary temperature below 32°C, or 36.5°C; Pain was assessed using diverse tools with varying measurement time points. Such discrepancies make the pooling of effects complex and difficult. (2) Resource Wastage and Under-evaluation: For instance, some studies report up to 20 outcomes, potentially leading to resource wastage, while others report only a single outcome, failing to capture the full spectrum of KMC's effects.

To address these critical issues, we propose two key solutions: (1) Standardization of Outcome Definitions: Future KMC-related research should prioritize the development of outcome indicators that adhere to standard definitions from relevant fields, adopt standardized definitions within the COMET classification system, or be guided by expert consensus meetings convened to unify definitions. (2) Development of a COS: Developing a COS for KMC would significantly help standardize outcome reporting, reduce heterogeneity, facilitate comparisons across studies, and promote the integration of findings.

### 4.3. Associations Between Core Domains

While our systematic categorization into five core outcome areas provides a structured overview, it is essential to recognize the complex, bidirectional interconnections among them. KMC is a holistic intervention, and its effects within one domain frequently influence, or are influenced by, outcomes in others.

The high prevalence of outcomes within the physiological/clinical core area (87.67% of studies) and the substantial attention given to the life impact core area (39.73% of studies) are particularly noteworthy. These two domains are intricately linked. For example, the immediate physiological benefits of KMC, such as improved respiratory stability, thermoregulation, and pain reduction, directly contribute to the neonate's health and stability. A healthier neonate typically alleviates parental psychological burdens, leading to positive outcomes in the life impact domain, such as reduced stress, anxiety, and depression. Furthermore, improvements in the life impact domain, such as reduced parental stress and enhanced parent–infant bonding, can, in turn, facilitate further physiological improvements. Less stressed and more confident parents are more likely to consistently implement KMC, thereby reinforcing and sustaining the infant's physiological benefits.

Moreover, the death core area, despite being less frequently reported (10.96% of studies), is fundamentally and closely related to all other outcomes. A successful KMC intervention that reduces mortality or improves survival inherently creates the opportunity for all other positive physiological, developmental, and life impact outcomes to be realized. The prevention of death is foundational for achieving any subsequent long-term benefits, including neurodevelopmental achievements and enhancements in family quality of life. This underscores the critical importance of robustly and consistently reporting mortality data, as it serves as the most fundamental measure of KMC's life-saving efficacy.

As evident from our systematic review, the comprehensive effectiveness of KMC lies not merely in its isolated effects within specific domains but in the dynamic interaction and mutually reinforcing benefits across physiological, developmental, psychological, and survival outcomes for both the infant and the family unit. Understanding these associations is key to a holistic evaluation of KMC's efficacy and crucial for guiding future research.

### 4.4. Strengths and Limitations

One of the key strengths of this study lies in its comprehensive and systematic approach to identifying outcomes related to KMC. The search strategy was broad, incorporating not only published articles but also clinical trial registration protocols, ensuring a thorough capture of all potential outcomes associated with KMC. Furthermore, the large number of studies included in the analysis guarantees the comprehensiveness of the outcomes, which is crucial for the development of a future COS. Given that the studies span the past decade, the outcomes presented reflect the current status of KMC implementation in recent years.

However, despite the robust data analysis, there are several limitations. First, we did not conduct a bias risk assessment or perform a meta-analysis, as the primary objective of this review was to provide a comprehensive description of all reported outcomes to inform the future development of a COS. Additionally, some studies lacked complete documentation of outcome definitions, and even when definitions were provided, significant variations existed across studies. This disparity in definitions posed challenges in integrating outcomes, as differences were too large to enable meaningful outcomes merging. Therefore, future research should prioritize standardizing outcome definitions to reduce heterogeneity. Finally, though we aimed for comprehensive outcome mapping, this review did not delve into certain related but distinct areas. For instance, we focused solely on reported outcomes and did not explore the various implementation strategies of KMC or the contextual factors influencing its fidelity across diverse settings. Similarly, while covering “all newborns,” specific subpopulations within KMC (e.g., extremely low-birth-weight infants, infants with specific comorbidities) might exhibit unique outcome profiles that warrant more focused investigation beyond the scope of this broad mapping review.

## 5. Conclusions

This is the first comprehensive systematic review to assess the reporting of outcomes in intervention studies involving KMC, providing the foundational step for the future development of a COS. The findings suggest that current research on KMC outcomes is relatively comprehensive, covering most outcome domains within five core areas, with a particular emphasis on physiological/clinical and life impact outcomes. However, the reporting of mortality, an essential clinical outcome, is notably sparse and requires more attention. Most of studies focus on three key outcomes—breastfeeding outcome, growth and development, and pain—assessing their effects through various approaches. Furthermore, significant heterogeneity in outcome reporting and definitions limits the comparability and synthesis of evidence across studies.

To further explore the effectiveness and mechanisms of KMC, future research should integrate multiomics technologies (e.g., genomics, metabolomics) to investigate the molecular mechanisms underlying KMC's effects on newborn physiology (e.g., thermoregulation) and psychology (attachment behaviors). Additionally, long-term follow-up studies should be conducted to evaluate KMC's impact on children's neurodevelopment, cognitive, and behaviors. To enhance mortality reporting, we recommend optimizing study designs by extending follow-up periods and adopting standardized mortality recording tools. For COS development, we propose establishing multicentered, multidisciplinary teams to standardize KMC outcomes through Delphi surveys and consensus meetings, prioritizing the inclusion of mortality as a core outcome. Creating a global COS promotion platform (e.g., an online database or international collaborative network) will facilitate outcome standardization and data sharing, enhancing the global applicability of KMC research, particularly in LICs and MICs.

This study offers multifaceted implications for both nursing practice and management. First, for clinical nurses, this research reveals the broad spectrum of outcomes associated with KMC intervention. It encourages nurses focusing not only on physiological aspects but also on the multidimensional impacts on newborns' psychological well-being and overall quality of life. This comprehensive understanding will empower nurses to provide parents with more thorough health education and guide them in effectively evaluating KMC's diverse benefits in clinical practice. Second, for nursing managers, this study underscores the critical importance of standardized outcome reporting. Managers should actively champion the adoption or participation in the future development and application of a COS for KMC within their institutions. This will ensure the comparability and integration of KMC-related data. Furthermore, our findings provide a reference for nursing managers when allocating resources and formulating training programs, thereby supporting the more effective implementation of KMC. Finally, this study serves as a foundational step for developing a KMC COS. In conclusion, developing a COS for KMC is crucial for reducing outcome heterogeneity, promoting study comparability, and facilitating evidence integration. By standardizing outcome reporting, the COS will enhance the evidence quality of KMC research, support evidence-based clinical practice, and deliver greater benefits to global newborn health, especially in resource-limited settings.

## Figures and Tables

**Table 1:** Characteristics of included studies.

Study characteristics	Number of studies (*n* [%])
Study type	
Article	162 (55.48)
Protocol	130 (44.52)
Study design	
RCT	240 (82.19)
Quasi-experimental study	34 (11.64)
Cross-over design	18 (6.17)
Year of publication	
2014–2018	110 (37.67)
2019–2024	182 (62.33)
Income group	
LICs	7 (2.40)
LMICs	134 (45.89)
UMICs	100 (34.25)
HICs	45 (15.41)
Multiple countries	6 (2.05)
Geographical region	
North America	19 (6.51)
Europe	24 (8.22)
Asia	179 (61.30)
South America	10 (3.42)
Africa	16 (5.48)
Oceania	1 (0.34)
Transcontinental countries	43 (14.73)
Sample size	
0–100	164 (56.16)
101–300	96 (32.88)
301–500	8 (2.74)
501∼	24 (8.22)
Mean	394
Study population	
Preterm infant	137 (46.92)
Very preterm infant	11 (3.77)
Extremely preterm infant	5 (1.71)
Full-term infant	59 (20.21)
Low-birth-weight infant	41 (14.04)
Very low-birth-weight infant	6 (2.05)
Others (mixed)	33 (11.30)
Intervention, type	
Mothers	218 (74.66)
Fathers	9 (3.08)
Mothers and fathers	31 (10.62)
Mothers and caregivers	15 (5.14)
Not reported	19 (6.50)
KMC duration	
Continue (> 20 h/d)	26 (8.90)
Intermittent (< 1 h/d)	60 (20.55)
Intermittent (1∼3 h/d)	97 (33.22)
Intermittent (3∼9 h/d)	33 (11.30)
Intermittent (> 10 h/d)	5 (1.71)
Not reported	71 (24.32)
KMC initiation	
Immediately after birth	59 (20.21)
Early (within first 72 h)	28 (9.59)
After hemodynamically/clinical stable	61 (20.89)
During procedure	40 (13.70)
During transport	3 (1.03)
After surgery	3 (1.03)
Not reported	98 (33.55)
Number of outcomes	
Max	20
Min	1
Mean	4
10–20	24 (8.22)
6–9	58 (19.86)
1–5	210 (71.92)

Abbreviations: HICs, high-income countries; KMC, kangaroo mother care; LICs, low-income countries; LMICs, lower-middle-income countries; UMICs, upper-middle-income countries.

**Table 2:** Distribution of the 142 unique outcome terms across COMET outcome taxonomy.

Core area	Unique outcome term (*n*, studies)	No. of unique outcomes
Death	1. Mortality/survival (*n* = 32)	1
	Mortality (*n* = 32)	

Physiological/clinical	2. Blood and lymphatic system outcomes (*n* = 6)	4
	Blood loss (*n* = 2)	
	Hb (*n* = 2)	
	Blood flow (*n* = 2)	
	Anemia (*n* = 1)	
	3. Cardiac outcomes (*n* = 79)	6
	HR (*n* = 63)	
	Cardiorespiratory stability (*n* = 15)	
	Blood pressure (*n* = 4)	
	PI (*n* = 2)	
	Bradycardic (*n* = 1)	
	PDA (*n* = 1)	
	5. Endocrine outcomes (*n* = 24)	5
	Cortisol (*n* = 19)	
	Oxytocin (*n* = 3)	
	Gastrin (*n* = 2)	
	Hypothyroidism (*n* = 1)	
	Serum prolactin (*n* = 1)	
	7. Eye outcomes (*n* = 2)	1
	ROP (*n* = 2)	
	8. Gastrointestinal outcomes (*n* = 41)	9
	Oral feeding outcomes (*n* = 28)	
	Feeding intolerance (*n* = 6)	
	Gut microbiome (*n* = 3)	
	Stool (*n* = 3)	
	Feeding method (*n* = 2)	
	NEC (*n* = 2)	
	Enteric enteropathy score (*n* = 1)	
	Vomiting (*n* = 1)	
	Motilin (*n* = 1)	
	9. General outcomes (*n* = 185)	10
	Weight/length/head circumference (*n* = 82)	
	Pain (*n* = 67)	
	Temperature (*n* = 47)	
	Hypothermia (*n* = 18)	
	Crying (*n* = 16)	
	Morbidity (*n* = 10)	
	Vital signs (*n* = 4)	
	Hyperthermia (*n* = 2)	
	Chest/arm circumference (cm) (*n* = 2)	
	Illness severity (*n* = 1)	
	10. Hepatobiliary outcomes (*n* = 10)	1
	Bilirubin (*n* = 10)	
	12. Infection and infestation outcomes (*n* = 21)	3
	Infection (*n* = 11)	
	Sepsis (*n* = 11)	
	Microbial colonization (*n* = 1)	
	14. Metabolism and nutrition outcomes (*n* = 15)	6
	Hypoglycemia (*n* = 7)	
	TPN duration (*n* = 2)	
	Blood sugar (*n* = 2)	
	Malnutrition (*n* = 2)	
	PH (*n* = 2)	
	Nutrition (*n* = 1)	
	17. Nervous system outcomes (*n* = 18)	7
	Brain function (*n* = 5)	
	Electromyographic activity (*n* = 3)	
	Cerebral oxygen (*n* = 3)	
	IVH (*n* = 3)	
	Epilepsy/seizure (*n* = 2)	
	Parasympathetic tone (*n* = 1)	
	Pediatric delirium (*n* = 1)	
	18. Pregnancy, puerperium, and perinatal outcomes (*n* = 84)	1
	Breastfeeding outcomes (*n* = 84)	
	19. Renal and urinary outcomes (*n* = 1)	1
	Urine (*n* = 1)	
	20. Reproductive system and breast outcomes (*n* = 5)	2
	Uterine involution (*n* = 4)	
	Precocious puberty (*n* = 1)	
	21. Psychiatric outcomes (*n* = 1)	
	Mental illness (*n* = 1)	1
	22. Respiratory, thoracic, and mediastinal outcomes (*n* = 78)	13
	Oxygen saturation (*n* = 49)	
	RR (*n* = 26)	
	Apnea (*n* = 9)	
	Respiratory support (*n* = 5)	
	Pneumonia (*n* = 2)	
	PS (*n* = 2)	
	BPD (*n* = 2)	
	NRDS (*n* = 2)	
	OxyHb (*n* = 2)	
	deoxyHb (*n* = 2)	
	Oxygen therapy duration (*n* = 1)	
	PaCO_2_ (*n* = 1)	
	Asthma (*n* = 1)	
	23. Skin and subcutaneous tissue outcomes (*n* = 3)	1
	Healing wounds (*n* = 3)	

Life	25. Physical functioning (*n* = 49)	10
	Neurodevelopmental (*n* = 19)	
	Sleep quality (*n* = 16)	
	Infant behavioral (*n* = 5)	
	Developmental (*n* = 3)	
	Fatigue (*n* = 2)	
	Cerebral palsy (*n* = 2)	
	Visual impairment (*n* = 1)	
	Disability (*n* = 1)	
	Movement (*n* = 1)	
	Physical disorders (*n* = 1)	
	26. Social functioning (*n* = 30)	5
	Bonding (*n* = 26)	
	School absenteeism (*n* = 2)	
	Maternal responsiveness (*n* = 1)	
	Perceived social support (*n* = 1)	
	Social health (*n* = 1)	
	27. Role functioning (*n* = 1)	1
	Father's role (*n* = 1)	
	28. Emotional functioning/well-being (*n *= 59)	11
	Depression (*n* = 29)	
	Stress (*n* = 18)	
	Anxiety (*n* = 14)	
	Resilience of mothers (*n* = 3)	
	Gene expression (*n* = 2)	
	Sense of competence (*n* = 2)	
	Self-efficacy (*n* = 2)	
	Happiness (*n* = 1)	
	Children's problem behavior (*n* = 1)	
	Women's wellbeing (*n* = 1)	
	Oxidative stress (*n* = 1)	
	29. Cognitive functioning (*n* = 13)	15
	Cognitive function (*n* = 13)	
	KMC cognitive (*n* = 2)	
	Preschool years (*n* = 2)	
	Math scores (*n* = 2)	
	Attentional (*n* = 2)	
	MDI (*n* = 1)	
	PDI (*n* = 1)	
	Executive function-preschool (*n* = 1)	
	Nursing knowledge (*n* = 1)	
	Years of school (*n* = 1)	
	Learning disability (*n* = 1)	
	IQ (*n* = 1)	
	Parents' experience (*n* = 1)	
	Perceived intensity (*n* = 1)	
	Locus of control (*n* = 1)	
	31. Perceived health status (*n* = 3)	2
	General health (*n* = 2)	
	Healthcare utilization (*n* = 1)	
	32. Delivery of care (*n* = 20)	3
	Satisfaction (*n* = 16)	
	Compliance (*n* = 3)	
	Participant's perspective (*n* = 1)	
	33. Personal circumstances (*n* = 4)	1
	Home environment (*n* = 4)	

Resource	34. Economic (*n* = 2)	1
	Cost (*n* = 2)	
	35. Hospital (*n* = 64)	14
	Hospital stay (*n* = 31)	
	KMC duration (*n* = 21)	
	NICU admission (*n* = 6)	
	Readmission (*n* = 4)	
	Appropriate care seeking practices (*n* = 3)	
	Early appropriate care seeking practices (*n* = 3)	
	Proportion of KMC (*n* = 3)	
	Duration of photo therapy (*n* = 1)	
	Time to first SSC (*n* = 1)	
	Time in arms (*n* = 1)	
	Transfer time (*n* = 1)	
	Reasons for KC incomplete (*n* = 1)	
	NICU noise (*n* = 1)	
	Preparation (*n* = 1)	
	37. Societal/carer burden (*n* = 2)	3
	Household out of pocket expenses for episode(s) (*n* = 1)	
	Consumption (*n* = 1)	
	Quality of maternal caregiving (*n* = 1)	

Adverse events	38. Adverse events/effects (*n* = 11)	4
	Adverse events (*n* = 11)	
	Rash (*n* = 2)	
	Frequent accidents (frequent falls) (*n* = 1)	
	Ventilation complication (*n* = 1)	

*Note:* PS—surfactant, oxyHb—oxygenated hemoglobin, deoxyHb—deoxygenated hemoglobin, PaCO_2_—partial pressure of carbon dioxide in arterial blood.

Abbreviations: BPD, bronchopulmonary dysplasia; HR, heart rate; IQ, intelligence quotient; IVH, intraventricular hemorrhage; MDI, Mental Developmental Index; NEC, necrotizing enterocolitis; NICU, neonatal intensive care unit; NRDS, neonatal respiratory distress syndrome; PDA, patent ductus arteriosus; PDI, Psychomotor Developmental Index; PH, potential of hydrogen; PI, Pulsatility Index; ROP, retinopathy of prematurity; RR: respiratory rate; TPN, total parenteral nutrition.

## Data Availability

The data that support the findings of this study are available from the corresponding authors upon reasonable request.

## Supporting Information

PRISMA 2020 Checklist.

Supporting Table S1: Search strategy.

Supporting Table S2: Definition and explanation of COMET outcome taxonomy.

Supporting Table S3: References of included studies.

Supporting Table S4: Characteristics of included studies.

Supporting Figure S1: Outcomes' distribution and study frequency.

## References

[1] World Health Organization (2024). Newborn Mortality: Key Facts [EB/OL]. https://www.who.int/news-room/fact-sheets/detail/newborn-mortality.

[2] Perin J., Mulick A., Yeung D. (2022). Global, Regional, and National Causes of Under 5 Mortality in 2000–2019: An Updated Systematic Analysis With Implications for the Sustainable Development Goals. *The Lancet Child & Adolescent Health*.

[3] Muhe L. M., McClure E. M., Nigussie A. K. (2019). Major Causes of Death in Preterm Infants in Selected Hospitals in Ethiopia (SIP): A Prospective, Cross-Sectional, Observational Study. *Lancet Global Health*.

[4] WHO Immediate KMC Study Group, Arya S., Naburi H., Kawaza K. (2021). Immediate ‘Kangaroo Mother Care’ and Survival of Infants With Low Birth Weight. *New England Journal of Medicine*.

[5] World Health Organization (2003). Kangaroo Mother Care: A Practical Guide [EB/OL]. https://www.who.int/publications/i/item/9241590351.

[6] Li Y. X., Hu Y. L., Chen Q. (2022). Clinical Practice Guideline for Kangaroo Mother Care in Preterm and Low Birth Weight Infants. *Journal of Evidence-Based Medicine*.

[7] Seo Y. S., Lee J., Ahn H. Y. (2016). Effects of Kangaroo Care on Neonatal Pain in South Korea. *Journal of Tropical Pediatrics*.

[8] Zhang X., Wang X., Juan J. (2023). Association of Duration of Skin-To-Skin Contact After Cesarean Delivery in China: A Superiority, Multicentric Randomized Controlled Trial. *American Journal of Obstetrics & Gynecology MFM*.

[9] Zhu L. B., Xu Y. H., Li J. F. (2022). The Effect of Kangaroo Mother Care After Duodenal Obstruction in Neonates. *Frontiers in Surgery*.

[10] Li Y. X., Li Y., Li X. (2025). Development of a Core Outcome Set and Core Measurement Set for Kangaroo Mother Care: a Study Protocol. *BMJ Open*.

[11] Abdulghani N., Edvardsson K., Amir L. H. (2018). Worldwide Prevalence of Mother-Infant Skin-to-Skin Contact After Vaginal Birth: A Systematic Review. *PLoS One*.

[12] Moore E. R., Bergman N., Anderson G. C., Medley N. (2016). Early Skin-To-Skin Contact for Mothers and Their Healthy Newborn Infants. *Cochrane Database of Systematic Reviews*.

[13] Williamson P. R., Altman D. G., Bagley H. (2017). The COMET Handbook: Version 1.0. *Trials*.

[14] Dodd S., Clarke M., Becker L., Mavergames C., Fish R., Williamson P. R. (2018). A Taxonomy Has Been Developed for Outcomes in Medical Research to Help Improve Knowledge Discovery. *Journal of Clinical Epidemiology*.

[15] The Core Outcome Measures in Effectiveness Trials (2018). *Outcome Classification [EB/OL]*.

[16] Cochrane (2018). Outcome Taxonomy User Guide. https://community.cochrane.org/sites/default/files/uploads/Outcome%20Taxonomy%20User%20Guide.pdf.

[17] Webbe J. W. H., Duffy J. M. N., Afonso E. (2020). Core Outcomes in Neonatology: Development of a Core Outcome Set for Neonatal Research. *Archives of Disease in Childhood-Fetal and Neonatal Edition*.

[18] Kocakabak C., van den Hoogen A., Rothfus M. (2025). Identifying Outcomes and Outcome Measures in Neonatal Family-Centered Care Trials: A Systematic Review. *Pediatric Research*.

[19] Klerk D. H., van Varsseveld O. C., Offringa M. (2024). Core Outcome Set for Necrotizing Enterocolitis Treatment Trials. *Pediatrics*.

[20] Knol M., Wang H., Bloomfield F. (2019). Development of a Core Outcome Set and Minimum Reporting Set for Intervention Studies in Growth Restriction in the Newborn (COSNEON): Study Protocol for a Delphi Study. *Trials*.

[21] Liberati A., Altman D. G., Tetzlaff J. (2009). The PRISMA Statement for Reporting Systematic Reviews and Meta-Analyses of Studies That Evaluate Health Care Interventions: Explanation and Elaboration. *Annals of Internal Medicine*.

[22] ClinicalTrials.gov. https://clinicaltrials.gov/.

[23] Platform tWictr. https://trialsearch.who.int.

[24] Registry tCCT. https://www.chictr.org.cn/index.html.

[25] Peng H., Shi J., Tang J. (2025). Outcome Reporting in Neonatal Septic Shock Studies: A Systematic Review. *Australian Critical Care*.

[26] Hall C., Shishkina A., Thurman R. (2024). Outcome Reporting in Cardio-Obstetrics Studies: A Systematic Review. *American Heart Journal*.

[27] Wong C., van Oostrom J., Bossuyt P. (2022). A Narrative Systematic Review and Categorisation of Outcomes in Inflammatory Bowel Disease to Inform a Core Outcome Set for Real-World Evidence. *Journal of Crohn’s and Colitis*.

[28] Villani L. A., Pavalagantharajah S., D’Souza R. (2020). Variations in Reported Outcomes in Studies on Vasa Previa: A Systematic Review. *American journal of obstetrics & gynecology MFM*.

[29] Charpak N., Montealegre-Pomar A., Bohorquez A. (2021). Systematic Review and Meta-Analysis Suggest That the Duration of Kangaroo Mother Care Has a Direct Impact on Neonatal Growth. *Acta Paediatrica*.

[30] Pados B. F., Hess F. (2020). Systematic Review of the Effects of Skin-to-Skin Care on Short-Term Physiologic Stress Outcomes in Preterm Infants in the Neonatal Intensive Care Unit. *Advances in Neonatal Care*.

[31] Montealegre-Pomar A., Bohorquez A., Charpak N. (2020). Systematic Review and Meta-Analysis Suggest That Kangaroo Position Protects Against Apnoea of Prematurity. *Acta Paediatrica*.

[32] Sharma D., Farahbakhsh N., Sharma S., Sharma P., Sharma A. (2019). Role of Kangaroo Mother Care in Growth and Breast Feeding Rates in Very Low Birth Weight (VLBW) Neonates: A Systematic Review. *Journal of Maternal-Fetal and Neonatal Medicine*.

[33] Mekonnen A. G., Yehualashet S. S., Bayleyegn A. D. (2019). The Effects of Kangaroo Mother Care on the Time to Breastfeeding Initiation Among Preterm and LBW Infants: A Meta-Analysis of Published Studies. *International Breastfeeding Journal*.

[34] Karimi F. Z., Sadeghi R., Maleki-Saghooni N., Khadivzadeh T. (2019). The Effect of Mother-Infant Skin to Skin Contact on Success and Duration of First Breastfeeding: A Systematic Review and Meta-Analysis. *Taiwanese Journal of Obstetrics & Gynecology*.

[35] Jafari M., Farajzadeh F., Asgharlu Z., Derakhshani N., Asl Y. P. (2019). Effect of Kangaroo Mother Care on Hospital Management Indicators: A Systematic Review and Meta-Analysis of Randomized Controlled Trials. *Journal of Education and Health Promotion*.

[36] Hatfield L. A., Murphy N., Karp K., Polomano R. C. (2019). A Systematic Review of Behavioral and Environmental Interventions for Procedural Pain Management in Preterm Infants. *Journal of Pediatric Nursing*.

[37] Ghojazadeh M., Hajebrahimi S., Pournaghi-Azar F., Mohseni M., Derakhshani N., Azami-Aghdash S. (2019). Effect of Kangaroo Mother Care on Successful Breastfeeding: A Systematic Review and Meta-Analysis of Randomised Controlled Trials. *Reviews on Recent Clinical Trials*.

[38] Cunningham C., Moore Z., Patton D., O’Connor T., Nugent L. E. (2018). Does Kangaroo Care Affect the Weight of Preterm/Low Birth-Weight Infants in the Neonatal Setting of a Hospital Environment?. *Journal of Neonatal Nursing*.

[39] Akbari E., Binnoon-Erez N., Rodrigues M. (2018). Kangaroo Mother Care and Infant Biopsychosocial Outcomes in the First Year: A Meta-Analysis. *Early Human Development*.

[40] Johnston C., Campbell-Yeo M., Disher T. (2017). Skin-to-Skin Care for Procedural Pain in Neonates. *Cochrane Database of Systematic Reviews*.

[41] Disher T., Benoit B., Johnston C., Campbell-Yeo M. (2017). Skin-to-Skin Contact for Procedural Pain in Neonates: Acceptability of Novel Systematic Review Synthesis Methods and GRADEing of the Evidence. *Journal of Advanced Nursing*.

[42] Conde-Agudelo A., Diaz-Rossello J. L. (2016). Kangaroo Mother Care to Reduce Morbidity and Mortality in Low Birthweight Infants. *Cochrane Database of Systematic Reviews*.

[43] Athanasopoulou E., Fox J. R. (2014). Effects of Kangaroo Mother Care on Maternal Mood and Interaction Patterns Between Parents and Their Preterm, Low Birth Weight Infants: A Systematic Review. *Infant Mental Health Journal*.

[44] Durmaz A., Sezici E., Akkaya D. D. (2023). The Effect of Kangaroo Mother Care or Skin-to-Skin Contact on Infant Vital Signs: A Systematic Review and Meta-Analysis. *Midwifery*.

[45] Zengin H., Suzan O. K., Hur G., Kolukısa T., Eroglu A., Cinar N. (2023). The Effects of Kangaroo Mother Care on Physiological Parameters of Premature Neonates in Neonatal Intensive Care Unit: A Systematic Review. *Journal of Pediatric Nursing*.

[46] Park J. J. H., Siden E., Harari O. (2020). Interventions to Improve Linear Growth During Exclusive Breastfeeding Life-Stage for Children Aged 0–6 Months Living in Low- and Middle-Income Countries: A Systematic Review With Network and Pairwise Meta-Analyses. *Gates Open Research*.

[47] Gadapani Pathak B., Sinha B., Sharma N., Mazumder S., Bhandari N. (2023). Effects of Kangaroo Mother Care on Maternal and Paternal Health: Systematic Review and Meta-Analysis. *Bulletin of the World Health Organization*.

[48] Sivanandan S., Sankar M. J. (2023). Kangaroo Mother Care for Preterm or Low Birth Weight Infants: A Systematic Review and Meta-Analysis. *BMJ Global Health*.

[49] Boundy E. O., Dastjerdi R., Spiegelman D. (2016). Kangaroo Mother Care and Neonatal Outcomes: A Meta-Analysis. *Pediatrics*.

[50] Yu Z. B., Han S. P., Xu Y. Q., Wong L. (2008). Meta-Analysis of Clinical Effect of Mother-Preterm Infant Skin-to-Skin Contact. *Zhonghua Hu Li Za Zhi*.

