# Supplementary material for: Outcome Reporting in Clinical Trials on Kangaroo Mother Care in Newborns: A Systematic Review for the Development of Core Outcome Set
Source: J Nurs Manag. 2025 Oct 9;2025:5662163. doi: 10.1155/jonm/5662163 (PMC12530923; doi:10.1155/jonm/5662163)
Supplement: Supporting Information — Additional supporting information can be found online in the Supporting Information section. [file 5662163.f1.pdf]

Supplementary Material

Context

PRISMA2020 CHECKLIST 2

SUPPLEMENTARY TABLE S1 SEARCH STRATEGY 6

SUPPLEMENTARY TABLE S2 THE DEFINITION AND EXPLANATION OF 38 CATEGORY 9

SUPPLEMENTARY TABLE S3 REFERENCES OF INCLUDED STUDIES 10

SUPPLEMENTARY TABLE S4 CHARACTERISTICS OF INCLUDED STUDIES 26

SUPPLEMENTARY FIGURE S1 74

EXPLANATION OF DIFFERENCE IN FROM THE REGISTERED PROTOCOL AND THE MANUSCRIPT 75

PRISMA2020 Checklist

Section and Topic	Item #	Checklist item	Location where item is reported
**TITLE**			
Title	1	Identify the report as a systematic review.	Page 1
**ABSTRACT**			
Abstract	2	See the PRISMA 2020 for Abstracts checklist.	Page 1-2
**INTRODUCTION**			
Rationale	3	Describe the rationale for the review in the context of existing knowledge.	Page 3-4
Objectives	4	Provide an explicit statement of the objective(s) or question(s) the review addresses.	Page 4
**METHODS**			
Eligibility criteria	5	Specify the inclusion and exclusion criteria for the review and how studies were grouped for the syntheses.	Page 5
Information sources	6	Specify all databases, registers, websites, organisations, reference lists and other sources searched or consulted to identify studies. Specify the date when each source was last searched or consulted.	Page 5; Figure 1
Search strategy	7	Present the full search strategies for all databases, registers and websites, including any filters and limits used.	Page 5; Supplementary Table 3
Selection process	8	Specify the methods used to decide whether a study met the inclusion criteria of the review, including how many reviewers screened each record and each report retrieved, whether they worked independently, and if applicable, details of automation tools used in the process.	Page 5-6
Data collection	9	Specify the methods used to collect data from reports, including how many reviewers collected data from each report, whether they worked	Page 6

Section and Topic	Item #	Checklist item	Location where item is reported
process		independently, any processes for obtaining or confirming data from study investigators, and if applicable, details of automation tools used in the process.	
Data items	10a	List and define all outcomes for which data were sought. Specify whether all results that were compatible with each outcome domain in each study were sought (e.g. for all measures, time points, analyses), and if not, the methods used to decide which results to collect.	Page 6
	10b	List and define all other variables for which data were sought (e.g. participant and intervention characteristics, funding sources). Describe any assumptions made about any missing or unclear information.	Page 6
Study risk of bias assessment	11	Specify the methods used to assess risk of bias in the included studies, including details of the tool(s) used, how many reviewers assessed each study and whether they worked independently, and if applicable, details of automation tools used in the process.	Page 5-6
Effect measures	12	Specify for each outcome the effect measure(s) (e.g. risk ratio, mean difference) used in the synthesis or presentation of results.	Page 6
Synthesis methods	13a	Describe the processes used to decide which studies were eligible for each synthesis (e.g. tabulating the study intervention characteristics and comparing against the planned groups for each synthesis (item #5)).	Page 6
	13b	Describe any methods required to prepare the data for presentation or synthesis, such as handling of missing summary statistics, or data conversions.	Not applicable
	13c	Describe any methods used to tabulate or visually display results of individual studies and syntheses.	Page 6
	13d	Describe any methods used to synthesize results and provide a rationale for the choice(s). If meta-analysis was performed, describe the model(s), method(s) to identify the presence and extent of statistical heterogeneity, and software package(s) used.	Page 6
	13e	Describe any methods used to explore possible causes of heterogeneity among study results (e.g. subgroup analysis, meta-regression).	Page 6
	13f	Describe any sensitivity analyses conducted to assess robustness of the synthesized results.	Not applicable
Reporting bias assessment	14	Describe any methods used to assess risk of bias due to missing results in a synthesis (arising from reporting biases).	Page 6

Section and Topic	Item #	Checklist item	Location where item is reported
Certainty assessment	15	Describe any methods used to assess certainty (or confidence) in the body of evidence for an outcome.	/
**RESULTS**			
Study selection	16a	Describe the results of the search and selection process, from the number of records identified in the search to the number of studies included in the review, ideally using a flow diagram.	Page 7; Figure 1
	16b	Cite studies that might appear to meet the inclusion criteria, but which were excluded, and explain why they were excluded.	Supplementary table 5
Study characteristics	17	Cite each included study and present its characteristics.	Supplementary table 5; Table 1
Risk of bias in studies	18	Present assessments of risk of bias for each included study.	/
Results of individual studies	19	For all outcomes, present, for each study: (a) summary statistics for each group (where appropriate) and (b) an effect estimate and its precision (e.g. confidence/credible interval), ideally using structured tables or plots.	Table 1-2
Results of syntheses	20a	For each synthesis, briefly summarise the characteristics and risk of bias among contributing studies.	Page 7
	20b	Present results of all statistical syntheses conducted. If meta-analysis was done, present for each the summary estimate and its precision (e.g. confidence/credible interval) and measures of statistical heterogeneity. If comparing groups, describe the direction of the effect.	Page 7-10
	20c	Present results of all investigations of possible causes of heterogeneity among study results.	Page 11-12
	20d	Present results of all sensitivity analyses conducted to assess the robustness of the synthesized results.	/
Reporting biases	21	Present assessments of risk of bias due to missing results (arising from reporting biases) for each synthesis assessed.	/

Section and Topic	Item #	Checklist item	Location where item is reported
Certainty of evidence	22	Present assessments of certainty (or confidence) in the body of evidence for each outcome assessed.	/
**DISCUSSION**			
Discussion	23a	Provide a general interpretation of the results in the context of other evidence.	Page 10-11
	23b	Discuss any limitations of the evidence included in the review.	Page 13-14
	23c	Discuss any limitations of the review processes used.	Page 13-14
	23d	Discuss implications of the results for practice, policy, and future research.	Page 15
**OTHER INFORMATION**			
Registration and protocol	24a	Provide registration information for the review, including register name and registration number, or state that the review was not registered.	Page 4
	24b	Indicate where the review protocol can be accessed, or state that a protocol was not prepared.	Page 4
	24c	Describe and explain any amendments to information provided at registration or in the protocol.	Page 4
Support	25	Describe sources of financial or non-financial support for the review, and the role of the funders or sponsors in the review.	Page 15
Competing interests	26	Declare any competing interests of review authors.	Page 15
Availability of data, code and other materials	27	Report which of the following are publicly available and where they can be found: template data collection forms; data extracted from included studies; data used for all analyses; analytic code; any other materials used in the review.	Page 15

Supplementary Table S1 Search strategy

**Ovid MEDLINE(R) ALL <2014 to July 7, 2024>**	
1	Intensive Care Units, Neonatal/
2	(NICU or neonatal ICU or newborn ICU or neonatal intensive care unit* or newborn intensive care unit*).ab,ti.
3	infant, newborn/
4	(neonat* OR infant* OR newborn* OR Newborn Infant* OR neonatology OR Low Birth Weight* OR Low-Birth-Weight* OR LBW or VLBW or ELBW preterm* or prematur* or pre-term).ab,ti.
5	1 or 2 or 3 or 4
6	Kangaroo-Mother Care Method/
7	(Kangaroo care OR Kangaroo-Mother Care OR KMC OR SSC OR skin to skin care OR skin-to-skin care OR Kangaroo Mother Care Method OR Kangaroo-Mother Care Methods).ab,ti.
8	6 or 7
9	5 and 8
**Embase<2014 to July 7, 2024>**	
1	neonatal intensive care unit/
2	(NICU or neonatal ICU or newborn ICU or neonatal intensive care unit* or newborn intensive care unit*).ab,ti.
3	newborn/
4	(neonat* or infant* or newborn* or Newborn Infant* or neonatology or Low Birth Weight* or Low-Birth-Weight* or LBW or VLBW or ELBW preterm* or prematur* or pre-term).ab,ti.
5	1 or 2 or 3 or 4
6	kangaroo care/
7	(Kangaroo care or Kangaroo-Mother Care or KMC or SSC or skin to skin care or skin-to-skin care or Kangaroo Mother Care Method or Kangaroo-Mother Care Methods).ab,ti.
8	6 or 7
9	5 and 8
CENTRAL - Cochrane Central Register of Controlled Trials <July 2024>	
1	Intensive Care Units, Neonatal/
2	(NICU or neonatal ICU or newborn ICU or neonatal intensive care unit* or newborn intensive care unit*).ti,ab.
3	infant, newborn/
4	(neonat* or infant* or newborn* or Newborn Infant* or neonatology or Low Birth Weight* or Low-Birth-Weight* or LBW or VLBW or ELBW preterm* or prematur* or pre-term).ti,ab.

5	1 or 2 or 3 or 4
6	Kangaroo-Mother Care Method/
7	(Kangaroo care or Kangaroo-Mother Care or KMC or SSC or skin to skin care or skin-to-skin care or Kangaroo Mother Care Method or Kangaroo-Mother Care Methods).ti,ab.
8	6 or 7
9	5 and 8
**CINAHL via EBSCO**	
S1	MH infant, newborn OR MH intensive care units, neonatal
S2	AB Kangaroo Mother Care
S3	S1 AND S2
**CNKI**	
1	SU=('新生儿'+'早产儿'+'低出生体重'+'新生儿重症监护室')*('袋鼠式护理'+'袋鼠护理'+'袋鼠妈妈护理'+' 袋鼠式护理模式'+'袋鼠式照护'+'皮肤对皮肤护理'+'袋鼠式母亲干预'+'母亲抚触护理')
**WanFang Data**	
1	(题名或关键词:("新生儿") or 题名或关键词:("早产儿") or 主题:(新生儿重症监护室)) and 题名或关键词:("袋鼠式护理")or 主题:(袋鼠护理)or 主题:(袋鼠妈妈护理)or 主题:(袋鼠式护理模式)or 主题:(袋鼠式照护)or 主题:(袋鼠式母亲干预)or 主题:(母亲抚触护理))
**SinoMed**	
1	婴儿，新生
2	“新生儿” OR “低出生体重儿” OR “极低出生体重儿” OR “超低出生体重儿” OR “早产儿” OR “极早产儿” OR “超早产儿” OR “小于胎龄儿” OR “适于胎龄儿” OR “大于胎龄儿” OR “足月儿” OR “过期产儿” OR “新生儿重症监护室”
3	1 or 2
4	母亲抚触疗法
5	“袋鼠式护理” OR “袋鼠护理” OR “袋鼠妈妈护理” OR “袋鼠式护理模式” OR “袋鼠式照护” OR “皮肤接触” OR “袋鼠式母亲干预” OR “母亲抚触护理”
6	4 or 5
7	3 and 6
**World Health Organization International Clinical Trials Registry Platform (WHO ICTRP)**	
Kangaroo care or Kangaroo-Mother Care or KMC or SSC or skin to skin care or skin-to-skin care or Kangaroo Mother Care Method or Kangaroo-Mother Care Methods	
**United States National Library of Medicine ClinicalTrials.gov (ClinicalTrials.gov)**	
Kangaroo care or Kangaroo-Mother Care or KMC or SSC or skin to skin care or skin-to-skin care or Kangaroo Mother Care Method or Kangaroo-Mother Care Methods	
**Chinese Clinical Trial Registry**	
kangaroo mother care OR KMC OR 袋鼠式护理 OR 袋鼠妈妈护理 OR 袋鼠护理	

Total results

Database/Register	Results
MEDLINE	n=1186
Embase	n=1812
CENTRAL	n=516
CINAHL	n=106
CNKI	n=557
WanFang Data	n=818
SinoMed	n=464
World Health Organization International Clinical Trials Registry Platform (WHO ICTRP)	n=183
United States National Library of Medicine ClinicalTrials.gov (ClinicalTrials.gov)	n=86
Chinese Clinical Trial Registry (Chictr)	n=2

Supplementary Table S2 the definition and explanation of 38 category

Core	Outcome domain	Explanation
**Death**	1: Mortality/survival	including overall (all-cause) survival/mortality rates and cause-specific survival/mortality rates, as well as composite survival outcomes including death (e.g., disease-free survival, progression-free survival, etc.).
**Physiological/ clinical**	2: Blood and lymphatic system outcomes<br>3: Cardiac outcomes<br>4: Congenital, familial and genetic outcomes<br>5: Endocrine outcomes<br>6: Ear and labyrinth outcomes<br>7: Eye outcomes<br>8: Gastrointestinal outcomes<br>9: General outcomes<br>10: Hepatobiliary outcomes<br>11: Immune system outcomes<br>12: Infection and infestation outcomes<br>13: Injury and poisoning outcomes<br>14: Metabolism and nutrition outcomes<br>15: Musculoskeletal and connective tissue<br>16: Outcomes relating to neoplasms: benign, malignant and unspecified (including cysts and polyps)<br>17: Nervous system outcomes<br>18: Pregnancy, puerperium, and perinatal outcomes<br>19: Renal and urinary outcomes<br>20: Reproductive system and breast outcomes<br>21: Psychiatric outcomes<br>22: Respiratory, thoracic and mediastinal outcomes<br>23: Skin and subcutaneous tissue outcomes<br>24: Vascular outcomes	‘‘General outcomes’’ include those affecting the whole body which cannot be attributed to a certain body system, for example, fatigue, chills, flu-like symptoms, malaise, anorexia, pain (unspecified, not associated with a particular body system), fever (not attributable to infection), anthropometric measures (e.g., weight), ‘‘global’’ measures, ‘‘symptoms’’ (not associated with a particular body system), ‘‘physical health’’, and fitness.<br>Laboratory parameters (e.g., from blood samples) and scientific measures (e.g., pharmacokinetic outcomes) should be classified within the physiological domain that captures the reason for the assessment (rather than within the ‘‘blood and lymphatic system’’ category, for example).<br>Breastfeeding be classified within the Pregnancy, puerperium, and perinatal outcomes.
**Life**	25: Physical functioning<br>26: Social functioning<br>27: Role functioning<br>28: Emotional functioning/well-being<br>29: Cognitive functioning<br>30: Global quality of life<br>31: Perceived health status<br>32: Delivery of care, including Satisfaction/patient preference, -Acceptability and availability, Adherence/compliance, Withdrawal from treatment, Appropriateness of treatment, Process, implementation, and service outcomes<br>33: Personal circumstances	Physical Function: The impact of disease/condition on daily life activities (e.g., walking ability, independence, cognitive ability, physical appearance, residual dysfunction, mobility, physical function, and health behavior).<br>Social Function: The impact of disease/condition on social functions (e.g., interaction ability, behavior, communication, social functioning, accommodation, and self-management).<br>Role Function: The impact of disease/condition on role performance (e.g., working ability).<br>Emotional Function/Well-being: The impact of disease/condition on mental health or overall well-being (e.g., response to stress, depression, coping ability, self-esteem, confidence, anxiety, perceptions of physical appearance, feelings of helplessness, and mood).<br>Cognitive Function: The impact of disease/condition on cognitive abilities (e.g., memory and attention, intellectual performance, and the ability to learn new things or apply knowledge, mental health, and cognitive function).<br>Knowledge Function: The impact of disease/condition on knowledge (e.g., memory loss, inability to concentrate, or poor knowledge retention) (e.g., academic learning and applied knowledge, mental functioning, and health literacy). Personal circumstances, Outcomes related to the patient's finances, family, and environment.
**Resource use**	34: Economic<br>35: Hospital<br>36: Need for further intervention<br>37: Societal/carer burden	‘‘hospital care’’ or ‘‘emergency care’’ fit within our ‘‘hospital’’ domain; ‘‘care at a general practice surgery, health clinic or other community setting,’’ and ‘‘health care at home’’ belong to our ‘‘societal/carer burden domain’’; and ‘‘medication’’ fits within our ‘‘need for further intervention’’ domain.
**Adverse events**	38: Adverse events/effects	Events that are considered as potential consequences of certain external factors (e.g., accidents, poor reactions, safety, injury, negative impacts, toxicity, and diseases). Specifically, named events should be categorized into appropriate outcome domains. If an event is classified as an adverse event, it should be classified within that domain.

Supplementary Table S3 references of included studies

1. Srinath BK, Shah J, Kumar P, et al. Kangaroo care by fathers and mothers: comparison of physiological and stress responses in preterm infants. J Perinatol. 2016;36(5):401-4.
2. WHO Immediate KMC Study Group, Arya S, Naburi H, Kawaza K, et al. Immediate “Kangaroo Mother Care” and Survival of Infants with Low Birth Weight. N Engl J Med. 2021;384(21):2028–2038.
3. Badiee Z, Faramarzi S, MiriZadeh T. The effect of kangaroo mother care on mental health of mothers with low birth weight infants. Adv Biomed Res. 2014;3:214. doi:10.4103/2277-9175.143262.
4. Sharma D, Murki S, Pratap OT. The effect of kangaroo ward care in comparison with "intermediate intensive care" on the growth velocity in preterm infant with birth weight <1100 g: randomized control trial. Eur J Pediatr. 2016;175(10):1317-1324. doi:10.1007/s00431-016-2766-y.
5. 冯晓燕,杨晓畅,徐红兵,等. 剖宫产术中即刻母婴皮肤接触对母乳喂养的影响. 中国护理管理, 2019, 19 (01): 48-51.
6. Sahlén Helmer C, Birberg Thornberg U, Frostell A, Örtenstrand A, Mörelius E. A Randomized Trial of Continuous Versus Intermittent Skin-to-Skin Contact After Premature Birth and the Effects on Mother-Infant Interaction. Adv Neonatal Care. 2020;20(3):E48-E56.
7. Jamehdar M, Nourizadeh R, Divband A, Valizadeh L, Hosseini M, Hakimi S. KMC by surrogate can have an effect equal to KMC by mother in improving the nutritional behavior and arterial oxygen saturation of the preterm infant: results of a controlled randomized clinical trial. BMC Pediatr. 2022;22(1):242. Published 2022 May 2. doi:10.1186/s12887-022-03316-z.
8. Jajoo M, Dhingra D, Chandil A, Jain R. Effect of Kangaroo Mother Care on Duration of Phototherapy on Neonatal Jaundice: A Randomized Controlled Trial. Indian J Pediatr. 2022;89(5):507-509. doi:10.1007/s12098-021-04013-y.
9. Pandita A, Panghal A, Gupta G, et al. Is kangaroo mother care effective in alleviating vaccination associated pain in early infantile period? A RCT. Early Hum Dev. 2018;127:69-73. doi:10.1016/j.earlhumdev.2018.10.001.
10. Shukla V, Chapla A, Uperiya J, Nimbalkar A, Phatak A, Nimbalkar S. Sucrose vs. skin to skin care for preterm neonatal pain control-a randomized control trial. J Perinatol. 2018;38(10):1365-1369. doi:10.1038/s41372-018-0193-9.
11. Abasalizadeh M, Kazemi F, Aghababaei S, Basiri B, Soltani F. Increasing the Resilience of Mothers With Preterm Infant: The Effect of Kangaroo Mother Care. Journal of Family & Reproductive Health. 2024;18(1):60-6.
12. Acharya N, Singh RR, Bhatta NK, Poudel P. Randomized control trial of Kangaroo Mother Care in low birth weight babies at a tertiary level hospital. Journal of Nepal Paediatric Society. 2014;34(1):18-23.
13. Adeli M, Aradmehr M. A comparative study of maternal-neonate abdominal and kangaroo (skin-to-skin) skin contact immediately after birth on maternal attachment behaviors up to 2 months. Journal of Education & Health Promotion. 2018;7:42.
14. Aghdas K, Talat K, Sepideh B. Effect of immediate and continuous mother-infant skin-to-skin contact on breastfeeding self-efficacy of primiparous women: a randomised control trial. Women & Birth: Journal of the Australian College of Midwives. 2014;27(1):37-40.
15. Agudelo SI, Gamboa OA, Acuna E, Aguirre L, Bastidas S, Guijarro J, et al. Randomized clinical

trial of the effect of the onset time of skin-to-skin contact at birth, immediate compared to early, on the duration of breastfeeding in full term newborns. International Breastfeeding Journal. 2021;16(1).
16. Avcin E, Kucukoglu S. The Effect of Breastfeeding, Kangaroo Care, and Facilitated Tucking Positioning in Reducing the Pain During Heel Stick in Neonates. Journal of Pediatric Nursing. 2021;61:410-6.
17. Bastani F, Rajai N, Farsi Zahra. The Effects of Kangaroo Care on the Sleep and Wake States of Preterm Infants. Journal of nursing research. 2017;25(3):231-9.
18. Bera A, Ghosh J, Singh AK, Hazra A, Mukherjee S, Mukherjee R. Effect of kangaroo mother care on growth and development of low birthweight babies up to 12 months of age: a controlled clinical trial. Acta Paediatr. 2014;103(6):643-50.
19. Bose A, Rath K, Nayak N. Impact of early skin-to-skin contact among cesarean section mother on breastfeeding, neonatal adaptation and maternal satisfaction. European Journal of Molecular and Clinical Medicine. 2021;8(2):2040-6.
20. Brotherton H, Gai A, Kebbeh B, Njie Y, Walker G, Muhammad AK, et al. Impact of early kangaroo mother care versus standard care on survival of mild-moderately unstable neonates <2000 grams: A randomised controlled trial. EClinicalMedicine. 2021;39:101050.
21. Caka SY, Topal S, Yurttutan S, Aytemiz S, Cikar Y, Sari M. Effects of kangaroo mother care on feeding intolerance in preterm infants. Journal of Tropical Pediatrics. 2023;69(2):06.
22. Campbell-Yeo M, Johnston CC, Benoit B, Disher T, Caddell K, Vincer M, et al. Sustained efficacy of kangaroo care for repeated painful procedures over neonatal intensive care unit hospitalization: a single-blind randomized controlled trial. Pain. 2019;160(11):2580-8.
23. Charpak N, Tessier R, Ruiz JG, Hernandez JT, Uriza F, Villegas J, et al. Twenty-year Follow-up of Kangaroo Mother Care Versus Traditional Care. Pediatrics. 2017;139(1):13-.
24. Chen E-M, Gau M-L, Liu C-Y, Lee T-Y. Effects of Father-Neonate Skin-to-Skin Contact on Attachment: A Randomized Controlled Trial. Nursing Research and Practice. 2017;2017.
25. Chidambaram AG, Manjula S, Adhisivam B, Bhat BV. Effect of Kangaroo mother care in reducing pain due to heel prick among preterm neonates: a crossover trial. Journal of Maternal-Fetal & Neonatal Medicine. 2014;27(5):488-90.
26. Choudhary M, Dogiyal H, Sharma D, Datt Gupta B, Madabhavi I, Choudhary JS, et al. To study the effect of Kangaroo Mother Care on pain response in preterm neonates and to determine the behavioral and physiological responses to painful stimuli in preterm neonates: a study from western Rajasthan. J Matern Fetal Neonatal Med. 2016;29(5):826-31.
27. Ciftci K, Yayan EH. The effect of three different methods applied during peripheral vascular access in prematures on pain and comfort levels. Journal of Pediatric Nursing. 2022;67:e129-e34.
28. Collados-Gomez L, Esteban-Gonzalo L, Lopez-Lopez C, Jimenez-Fernandez L, Piris-Borregas S, Garcia-Garcia E, et al. Lateral kangaroo care in hemodynamic stability of extremely preterm infants: Protocol study for a non-inferiority randomized controlled trial cangulat. International Journal of Environmental Research and Public Health. 2022;19(1) (no pagination).
29. Cong X, Ludington-Hoe SM, Hussain N, Cusson RM, Walsh S, Vazquez V, et al. Parental oxytocin responses during skin-to-skin contact in pre-term infants. Early Human Development. 2015;91(7):401-6.
30. Cooijmans KHM, Beijers R, Brett BE, de Weerth C. Daily skin-to-skin contact in full-term infants and breastfeeding: Secondary outcomes from a randomized controlled trial. Maternal & Child Nutrition. 2022;18(1):e13241.

31. Cortes D, Maldonado D, Gallego J, Charpak N, Tessier R, Ruiz JG, et al. Comparing long-term educational effects of two early childhood health interventions. Journal of Health Economics. 2022;86:102693.
32. Coskun D, Gunay U. The Effects of Kangaroo Care Applied by Turkish Mothers who Have Premature Babies and Cannot Breastfeed on Their Stress Levels and Amount of Milk Production. Journal of Pediatric Nursing. 2020;50:e26-e32.
33. Dehghani K, Movahed ZP, Dehghani H, Nasiriani K. A randomized controlled trial of kangaroo mother care versus conventional method on vital signs and arterial oxygen saturation rate in newborns who were hospitalized in neonatal intensive care unit. Journal of Clinical Neonatology. 2015;4(1):26-31.
34. Deng Q LQ, Wang H, Sun H, Xu X. Early father-infant skin-to-skin contact and its effect on the neurodevelopmental outcomes of moderately preterm infants in China: study protocol for a randomized controlled trial. . Trials. 2018;19(1):701.
35. Dezhdar S, Jahanpour F, Bakht SF, Ostovar A. The effects of kangaroo mother care and swaddling on venipuncture pain in premature neonates: A randomized clinical trial. Iranian Red Crescent Medical Journal. 2016;18(4).
36. Diniz KT, Cabral Filho JE, Miranda RM, Souza Lima GM, dos Santos Figueredo NP, Nunes de Araujo KF. Short-time effect of the kangaroo position on electromyographic activity of premature infants: a randomized clinical trial. Jornal De Pediatria. 2020;96(6):741-7.
37. Eckermann HA, Meijer J, Cooijmans K, Lahti L, de Weerth C. Daily skin-to-skin contact alters microbiota development in healthy full-term infants. Gut Microbes. 2024;16(1):2295403.
38. Eksirinimit T, Punthmatharith B, Bansopit N, Kusol K. Effects of Kangaroo Care on body temperature of premature infants and maternal satisfaction at Maharaj Nakhon Si Thammarat hospital, Thailand. Journal of neonatal nursing. 2023;Vol.29(2):302-6p.
39. El Sehmawy AA, Younes Abd Elaziz S, Elwahed RMA, Elsheikh AA. Skin-to-skin contact and its effect on mothers' postpartum psychological distress and their full-term neonate in Egypt. Journal of Tropical Pediatrics. 2023;69(3):05.
40. El-Farrash RA, Shinkar DM, Ragab DA, Salem RM, Saad WE, Farag AS, et al. Longer duration of kangaroo care improves neurobehavioral performance and feeding in preterm infants: a randomized controlled trial. Pediatr Res. 2020;87(4):683-8.
41. Erduran B, Yaman Sozbir S. Effects of intermittent kangaroo care on maternal attachment, postpartum depression of mothers with preterm infants. Journal of Reproductive & Infant Psychology. 2023;41(5):556-65.
42. Fallah R, Naserzadeh N, Ferdosian F, Binesh F. Comparison of effect of kangaroo mother care, breastfeeding and swaddling on Bacillus Calmette-Guerin vaccination pain score in healthy term neonates by a clinical trial. Journal of Maternal-Fetal & Neonatal Medicine. 2017;30(10):1147-50.
43. Forde D, Deming DD, Tan JC, Phillips RM, Fry-Bowers EK, Barger MK, et al. Oxidative Stress Biomarker Decreased in Preterm Neonates Treated With Kangaroo Mother Care. Biological Research for Nursing. 2020;22(2):188-96.
44. Gavhane S, Eklare D, Mohammad H. Long Term Outcomes of Kangaroo Mother Care in Very Low Birth Weight Infants. Journal of Clinical and Diagnostic Research JCDR. 2016;10(12):SC13-SC5.
45. Gere S, Berhane Y, Worku A. Comparison of Chest-to-Back Skin-to-Skin Contact and Chest-to-Chest Skin-to-Skin Contact on the Risk of Oxygen Desaturation and Change in Heart Rate in Low Birth Weight and/or Premature Babies: A Randomized Controlled Clinical Trial. International Journal of Pediatrics. 2021;2021:7196749.

46. Gere S, Berhane Y, Worku A. Chest-to-Back Skin-to-Skin Contact to Regulate Body Temperature for Low Birth Weight and/or Premature Babies: A Crossover Randomized Controlled Clinical Trial. Int J Pediatr. 2021;2021:8873169.
47. Gholami A, Karimi F, Ghasempour Z, Abolhassani M, Rabiee N. Comparison of the effect of kangaroo care and infant massage on the level of maternal anxiety and neonatal pain. J Babol University of Medical Sciences. 2021;23(1):90-7.
48. Goudarzvand L, Dabirian A, Nourian M, Jafarimanesh H, Ranjbaran M. Comparison of conventional phototherapy and phototherapy along with Kangaroo mother care on cutaneous bilirubin of neonates with physiological jaundice. J Matern Fetal Neonatal Med. 2017;32(8):1280-1284.
49. Hanum P, Harahap SW, Silaban VF, Silalahi KL, Harahap RF, Anggeria E. The Effect of Kangaroo Mother Care on Rooting - Sucking Reflex on Low Birthweight Infant. Open Access Macedonian Journal of Medical Sciences. 2022;Part E. 10:550-4.
50. Hardin JS, Jones NA, Mize KD, Platt M. Parent-Training with Kangaroo Care Impacts Infant Neurophysiological Development & Mother-Infant Neuroendocrine Activity. Infant Behavior & Development. 2020;58:N.PAG-N.PAG.
51. Huang JZ, Chen CN, Lee CP, Kao CH, Hsu HC, Chou AK. Evaluation of the Effects of Skin-to-Skin Contact on Newborn Sucking, and Breastfeeding Abilities: A Quasi-Experimental Study Design. Nutrients. 2022;14(9):28.
52. Huang X CL, Zhang L. Effects of Paternal Skin-to-Skin Contact in Newborns and Fathers After Cesarean Delivery. . J Perinat Neonatal Nurs. 2019;33(1):68-73.
53. Hucklenbruch-Rother E, Vohlen C, Mehdiani N, Keller T, Roth B, Kribs A, et al. Delivery room skin-to-skin contact in preterm infants affects long-term expression of stress response genes. Psychoneuroendocrinology.122:104883.
54. HX. G, GX. X, Dong R, Fu H, Wang D, Zhang H. Effect of repeated Kangaroo Mother Care on repeated procedural pain in preterm infants: A randomized controlled trial. International journal of nursing studies. 2015;52(7):1157-65.
55. Jabraeili M, Seyedrasouli A, Kheiri Z, Sadeghi-Bazrgani H, Jannatdoost A. Impact of skin-to-skin care on satisfaction and experience of cesarean mothers: A randomized, double-blinded clinical trial. Annals of Tropical Medicine and Public Health. 2017;10(6):1756-62.
56. Jajoo M, Mittal M, Saikia D, Kumar N. Impact of Kangaroo Mother Care on Heart Rate Variability and Pain Score with Heel Lance Pain in Preterm Neonates: A Cross-over Randomized Controlled Trial. Journal of Neonatology. 2024;38(2):213-8.
57. Jamil S, Rehman A, Shahman M, Saleem MI, Hafeez R, Akram S. Outcome of Intermittent Kangaroo Mother Care in Neonatal Intensive Care Unit. Pakistan Journal of Medical and Health Sciences. 2021;15(11):3106-8.
58. Jayaraman D, Mukhopadhyay K, Bhalla AK, Dhaliwal LK. Randomized Controlled Trial on Effect of Intermittent Early Versus Late Kangaroo Mother Care on Human Milk Feeding in Low-Birth-Weight Neonates. Journal of Human Lactation. 2017;33(3):533-9.
59. Kapoor A, Khan MA, Beohar V. Pain Relief in Late Preterm Neonates: A Comparative Study of Kangaroo Mother Care, Oral Dextrose 50%, and Supine Nesting Position. International Journal of Applied & Basic Medical Research. 2021;11(3):188-91.
60. Karimi FZ, Khadivzadeh T, Saeidi M, Bagheri S. The Effect of Kangaroo Mother Care Immediately after Delivery on Mother-infant Attachment 3 Months after Delivery. International Journal of Pediatrics-Mashhad. 2016;4(9):3561-70.

61. Karimi S, Parsa P, Basiri B, Roshanaei G. The effect of kangaroo mother care on nutritional status and duration of hospitalization of premature infants in Iran. Journal of Postgraduate Medical Institute. 2020;34(1):16-21.
62. Khadivzadeh T, Karimi FZ, Tara F, Bagheri S. The Effect of Postpartum Mother-Infant Skin-to-Skin Contact on Exclusive Breastfeeding in Neonatal Period: A Randomized Controlled Trial. International Journal of Pediatrics-Mashhad. 2017;5(7):5409-17.
63. Kim Chi L, Tien Long N, Duy Huong Huynh T, Carrara HPO, Bergman NJ. Newly born low birthweight infants stabilise better in skin-to-skin contact than when separated from their mothers: a randomised controlled trial. Acta Paediatrica. 2016;105(4):381-90.
64. Kollmann M, Aldrian L, Scheuchenegger A, Mautner E, Herzog SA, Urlesberger B, et al. Early skin-to-skin contact after cesarean section: A randomized clinical pilot study. PLoS ONE [Electronic Resource]. 2017;12(2):e0168783.
65. Kristoffersen L, Bergseng H, Engesland H, Bagstevold A, Aker K, St, et al. Skin-to-skin contact in the delivery room for very preterm infants: a randomised clinical trial. BMJ paediatrics open. 2023;7(1).
66. Kristoffersen L, Stoen R, Rygh H, Sognnaes M, Follestad T, Mohn HS, et al. Early skin-to-skin contact or incubator for very preterm infants: study protocol for a randomized controlled trial. Trials. 2016;17(1):593.
67. Kucukoglu S, Yilmaz Kurt F, Aytekin Ozdemir A, Ozcan Z. The Effect of Kangaroo Care on Breastfeeding and Development in Preterm Neonates. Journal of Pediatric Nursing. 2021;60:e31-e8.
68. Kumbhojkar S MY, Sarawade S. Kangaroo mother care (KMC): an alternative to conventional method of care for low birth weight babies. International Journal of Health Sciences and Research. 2016;6(3):36-42.
69. Kurt FY, Kucukoglu S, Ozdemir AA, Ozcan Z. The effect of kangaroo care on maternal attachment in preterm infants. Nigerian Journal of Clinical Practice. 2020;23(1):26-32.
70. Lamy Filho F, de Sousa SH, Freitas IJ, Lamy ZC, Simoes VM, da Silva AA, et al. Effect of maternal skin-to-skin contact on decolonization of Methicillin-Oxacillin-Resistant Staphylococcus in neonatal intensive care units: a randomized controlled trial. BMC Pregnancy & Childbirth. 2015;15:63.
71. Li W. Efect evaluation of kangaroo mother care in Liping area, Guizhou province,China. 2022.
72. Li X, Zhang Y, Li W. Kangaroo mother care could significantly reduce the duration of phototherapy for babies with jaundice. International Journal of Clinical and Experimental Medicine. 2017;10(1):1690-5.
73. Liao Q, Chen L, Fu S, Hu H. Effect of kangaroo mother care method on pain, growth and breastfeeding in newborns. International Journal of Clinical and Experimental Medicine. 2020;13(6):4403-8.
74. Linner A, Klemming S, Sundberg B, Lillieskold S, Westrup B, Jonas W, et al. Immediate skin-to-skin contact is feasible for very preterm infants but thermal control remains a challenge. Acta Paediatrica, International Journal of Paediatrics. 2020;109(4):697-704.
75. Linner A, Lode Kolz K, Klemming S, Bergman N, Lillieskold S, Markhus Pike H, et al. Immediate skin-to-skin contact may have beneficial effects on the cardiorespiratory stabilisation in very preterm infants. Acta Paediatrica. 2022;111(8):1507-14.
76. Linner A, Westrup B, Rettedal S, Kawaza K, Naburi H, Newton S, et al. Immediate skin-to-skin contact for low birth weight infants is safe in terms of cardiorespiratory stability in limited-resource settings. Global Pediatrics. 2023;3:None.
77. Lode-Kolz K, Hermansson C, Linner A, Klemming S, Hetland HB, Bergman N, et al. Immediate

skin-to-skin contact after birth ensures stable thermoregulation in very preterm infants in high-resource settings. Acta Paediatrica, International Journal of Paediatrics. 2023;112(5):934-41.
78. Lumbanraja SN. Influence of maternal factors on the successful outcome of kangaroo mother care in low birth-weight infants: a randomized controlled trial. Journal of neonatal-perinatal medicine. 2016;9(4):385-92.
79. Mansoori M, Salmani N. Effect of Breast Milk Expression during Kangaroo Mother Care on Milk Volume in Mothers with Premature Infants Admitted to Neonatal Intensive Care Unit. Journal of Evidence-based Care. 2020;10(1):44-50.
80. Manzoor N, Sehar S, Afzal M, Gilani SA. Effect of kangaroo mother care on exclusive breast feeding in low birth weight infants. Rawal Medical Journal. 2023;48(3):692-4.
81. Marulli A, Kamlin COF, Dawson JA, Donath SM, Davis PG, Lorenz L. The effect of skin-to-skin care on cerebral oxygenation during nasogastric feeding of preterm infants. Acta Paediatrica, International Journal of Paediatrics. 2018;107(3):430-5.
82. Mawarti R, Fitriahadi E, Utami I, Intarti WD. Effectivity of KMC and KFC methods on newborn babies body temperature in bpm istri utami sleman. Pakistan Journal of Medical and Health Sciences. 2021;15(3):1144-6.
83. Mazumder S, Taneja S, Dube B, Bhatia K, Ghosh R, Shekhar M, et al. Effect of community-initiated kangaroo mother care on survival of infants with low birthweight: a randomised controlled trial. Lancet. 2019;394 North American Edition(10210):1724-36.
84. Mehler K, Hucklenbruch-Rother E, Trautmann-Villalba P, Becker I, Roth B, Kribs A. Delivery room skin-to-skin contact for preterm infants-A randomized clinical trial. Acta Paediatrica. 2020;109(3):518-26.
85. Miranda RM, Cabral Filho JE, Diniz KT, Clough GF, Alves JGB, Lima GMS, et al. Effect of Kangaroo Position on microcirculation of preterm newborns: a controlled randomized clinical trial. Jornal de pediatria. 2022;Vol.98(2):196-203p.
86. Mirnia K, Bostanabad MA, Asadollahi M, Razzaghi MH. Paternal skin-to-skin care and its effect on cortisol levels of the infants. Iranian Journal of Pediatrics. 2017;27(1).
87. Mohammadi M, Bergh AM, Heidarzadeh M, Hosseini M, Sattarzadeh Jahdi N, Valizadeh L, et al. Implementation and effectiveness of continuous kangaroo mother care: a participatory action research protocol. International Breastfeeding Journal. 2021;16(1):24.
88. Morelius E, Ortenstrand A, Theodorsson E, Frostell A. A randomised trial of continuous skin-to-skin contact after preterm birth and the effects on salivary cortisol, parental stress, depression, and breastfeeding. Early Human Development. 2015;91(1):63-70.
89. Mosayebi Z, Javidpour M, Rahmati M, Hagani H, Movahedian AH. The effect of Kangaroo mother care on pain from heel lance in preterm newborns admitted to neonatal intensive care unit: A crossover randomized clinical trial. Journal of Comprehensive Pediatrics. 2014;5(4).
90. Nimbalkar AS, Patel DV, Nimbalkar SM, Patel VK, Patel DN, Phatak AG. Infant and Young Child Feeding Practices in Infants Receiving Skin to Skin Care at Birth: Follow-up of Randomized Cohort. Journal of Clinical and Diagnostic Research JCDR. 2016;10(12):SC09-SC12.
91. Nimbalkar S, Popat V, Patel P, Pujara R, Shinde M, Patel D. Effect of Kangaroo Mother Care Transport in Preventing Moderate Hypothermia in Low Birth Weight Babies During Transportion to Home After Discharge: A Randomized Controlled Trial. Indian Pediatrics. 2023;60(4):272-6.
92. Nimbalkar S, Shukla VV, Chauhan V, Phatak A, Patel D, Chapla A, et al. Blinded randomized crossover trial: Skin-to-skin care vs. sucrose for preterm neonatal pain. Journal of Perinatology.

2020;40(6):896-901.
93. Obaid Ur Rehman M, Hayat S, Gul R, Irfan Waheed KA, Victor G, Khan MQ. Impact of intermittent kangaroo mother care on weight gain of neonate in nicu: Randomized control trial. JPMA - Journal of the Pakistan Medical Association. 2020;70(6):973-7.
94. Parsa P, Karimi S, Basiri B, Roshanaei G. The effect of kangaroo mother care on physiological parameters of premature infants in Hamadan City, Iran. The Pan African medical journal. 2018;30:89.
95. Patel DV, Soni SN, Shukla VV, Phatak AG, Shinde MK, Nimbalkar AS, et al. Efficacy of Skin-to-Skin Care versus Swaddling for Pain Control Associated with Vitamin K Administration in Full-Term Neonates: A Randomized Controlled Trial. Journal of Tropical Pediatrics. 2022;68(4):06.
96. Pujara RK, Upadhyay V, Thacker JP, Rana BB, Patel SS, Joshi JM, et al. Efficacy of skin-to-skin vs. cloth-to-cloth contact for thermoregulation in low birth weight newborns: a randomized crossover trial. Journal of Tropical Pediatrics. 2023;69(2):06.
97. Ramani M, Choe EA, Major M, Newton R, Mwenechanya M, Travers CP, et al. Kangaroo mother care for the prevention of neonatal hypothermia: a randomised controlled trial in term neonates. Archives of Disease in Childhood. 2018;103(5):492-7.
98. Rangey PS, Sheth M. Comparative Effect of Massage Therapy versus Kangaroo Mother Care on Body Weight and Length of Hospital Stay in Low Birth Weight Preterm Infants. International Journal of Pediatrics. 2014;2014:434060.
99. Rasouli Larma'i N, Ahmadpour-Kacho M, Zahed Pasha Y, Hajiahmadi M, Mazloomi A. The effect of kangaroo mother care on the duration of phototherapy in term infants with hyperbilirubinemia. [Persian]. Journal of Babol University of Medical Sciences. 2016;18(6):15-20.
100. Rheinheimer N, Beijers R, Bruinhof N, Cooijmans KHM, de Weerth C. Effects of daily full-term infant skin-to-skin contact on behavior and cognition at age three - secondary outcomes of a randomized controlled trial. Journal of Child Psychology & Psychiatry & Allied Disciplines. 2023;64(1):136-44.
101. Rheinheimer N, Beijers R, Cooijmans KHM, Brett BE, de Weerth C. Effects of skin-to-skin contact on full-term infants' stress reactivity and quality of mother-infant interactions. Developmental Psychobiology. 2022;64(7):e22308.
102. Ropars S, Tessier R, Charpak N, Uriza LF. The long-term effects of the Kangaroo Mother Care intervention on cognitive functioning: Results from a longitudinal study. Developmental Neuropsychology. 2018;43(1):82-91.
103. Samra H, Dutcher J, McGrath JM, Foster M, Klein L, Djira G, et al. Effect of Skin-to-Skin Holding on Stress in Mothers of Late-Preterm Infants A Randomized Controlled Trial. Advances in Neonatal Care. 2015;15(5):354-64.
104. Sen E, Manav G. Effect of Kangaroo Care and Oral Sucrose on Pain in Premature Infants: A Randomized Controlled Trial. Pain management nursing : official journal of the American Society of Pain Management Nurses. 2020;04:1-9.
105. Seo YS, Lee J, Ahn HY. Effects of Kangaroo Care on Neonatal Pain in South Korea. Journal of Tropical Pediatrics. 2016;62(3):246-9.
106. Sharma A. Efficacy of early skin-to-skin contact on the rate of exclusive breastfeeding in term neonates: a randomized controlled trial. African Health Sciences. 2016;16(3):790-7.
107. Sharma D, Murki S, Oleti TP. Study comparing "Kangaroo Ward Care" with "Intermediate Intensive Care" for improving the growth outcome and cost effectiveness: randomized control trial. J Matern Fetal Neonatal Med. 2018;31(22):2986-93.
108. Shattnawi KK, Al-Ali N. The Effect of Short Duration Skin to Skin Contact on Premature Infants'

Physiological and Behavioral Outcomes: A Quasi-Experimental Study. Journal of Pediatric Nursing. 2019;46:e24-e8.
109. Shukla VV, Bansal S, Nimbalkar A, Chapla A, Phatak A, Patel D, et al. Pain Control Interventions in Preterm Neonates: a Randomized Controlled Trial. Indian pediatrics. 2018;55(4):292-6.
110. Shukla VV, Chaudhari AJ, Nimbalkar SM, Phatak AG, Patel DV, Nimbalkar AS. Skin-to-Skin Care by Mother vs. Father for Preterm Neonatal Pain: A Randomized Control Trial (ENVIRON Trial). International Journal of Pediatrics (United Kingdom). 2021;2021 (no pagination).
111. Singh K, Chawla D, Jain S, Khurana S, Takkar N. Immediate skin-to-skin contact versus care under radiant warmer at birth in moderate to late preterm neonates - A randomized controlled trial. Resuscitation. 2023;189:109840.
112. Singh P, Arif F, Kumar N. A Hospital Based Prospective Study to Compare the Kangaroo Mother Care (Kmc) with Conventional Method of Care (Cmc) in the Care of Low Birth Weight Infants (Lbwi) at Tertiary Care Center. International Journal of Medicine and Public Health. 2024;14(2):329-32.
113. Sinha B, Sommerfelt H, Ashorn P, Mazumder S, More D, Taneja S, et al. Effect of Community-Initiated Kangaroo Mother Care on Fecal Biomarkers of Gut Function in Low Birth Weight Infants in North India: A Randomized Clinical Trial. American Journal of Tropical Medicine and Hygiene. 2022;106(3):945-52.
114. Soltani S, Zohoori D, Adineh M. Comparison the Effectiveness of Breastfeeding, Oral 25% Dextrose, Kangaroo-Mother Care Method, and EMLA Cream on Pain Score Level Following Heal Pick Sampling in Newborns: a randomized clinical trial. Electronic Physician [Electronic Resource]. 2018;10(5):6741-8.
115. Srinath BK, Shah J, Kumar P, Shah PS. Kangaroo care by fathers and mothers: comparison of physiological and stress responses in preterm infants. Journal of Perinatology. 2016;36(5):401-4.
116. Srivastava G, Garg A, Chhavi N, Faridi M. Effect of kangaroo mother care on pain during orogastric tube insertion in low-birthweight newborns: An open label, randomised trial. Journal of Paediatrics & Child Health. 2022;58(12):2248-53.
117. Srivastava S GA, Bhatnagar A, Dutta S. Effect of very early skin to skin contact on success at breastfeeding and preventing early hypothermia in neonates. Indian Journal of Public Health Research and Development. 2014;58(1):22-6.
118. Swarnkar K, Vagha J. Effect of kangaroo mother care on growth and morbidity pattern in low birth weight infants. Journal of Krishna Institute of Medical Sciences University. 2016;5(1):91-9.
119. Taneja S, Sinha B, Upadhyay RP, Mazumder S, Sommerfelt H, Martines J, et al. Community initiated kangaroo mother care and early child development in low birth weight infants in India-a randomized controlled trial. Bmc Pediatrics. 2020;20(1).
120. Tully KP, Holditch-Davis D, White-Traut RC, David R, O'Shea TM, Geraldo V. A Test of Kangaroo Care on Preterm Infant Breastfeeding. JOGNN - Journal of Obstetric, Gynecologic, & Neonatal Nursing. 2016;45(1):45-61.
121. Tumukunde V, Medvedev MM, Tann CJ, Mambule I, Pitt C, Opondo C, et al. Effectiveness of kangaroo mother care before clinical stabilisation versus standard care among neonates at five hospitals in Uganda (OMWaNA): a parallel-group, individually randomised controlled trial and economic evaluation. Lancet. 2024;403(10443):2520-32.
122. uuml;nd, uuml;z, Uuml, Ouml;zt, uuml;rk S. The Effect of Skin-to-Skin Contact on Placental Separation Time and Initiation of Breastfeeding. Breastfeeding medicine. 2023;Vol.18(7):522-7p.
123. uuml;rker TK, ouml;zen D. The Effect of Kangaroo Care and Breastfeeding on Reducing the Pain

due to Hepatitis B Vaccine Injection in Newborn Infants: a Comparative Analysis. Journal of pediatric research. 2022;Vol.9(3):252-8p.
124. Veeraiah R, Mangalgi S, Maralusiddappa PGC, Veerabhadraiah KM, Shenoy S. Effect of Kangaroo Care Following Immersion Bath on Body Temperature in Preterm Neonates. Journal of Neonatology. 2023;37(4):307-10.
125. WANG. 袋鼠式护理对母乳喂养 新生儿神经行为及新生儿疼痛的影响. 2018.
126. Wang Y, Dong W, Zhang L, Zhang R. The effect of kangaroo mother care on aEEG activity and neurobehavior in preterm infants: a randomized controlled trial. Journal of Maternal-Fetal & Neonatal Medicine. 2022;35(25):6483-8.
127. Wang Y, Zhang L, Dong W, Zhang R. Effects of Kangaroo Mother Care on Repeated Procedural Pain and Cerebral Oxygenation in Preterm Infants. American Journal of Perinatology. 2023;40(8):867-73.
128. Wang Y, Zhao T, Zhang Y, Li S, Cong X. Positive Effects of Kangaroo Mother Care on Long-Term Breastfeeding Rates, Growth, and Neurodevelopment in Preterm Infants. Breastfeeding Medicine: The Official Journal of the Academy of Breastfeeding Medicine. 2021;02:02.
129. Yildirim F, Buyukkayaci Duman N, Sahin E, Vural G. The Effect of Kangaroo Care on Paternal Attachment: A Randomized Controlled Study. Advances in Neonatal Care. 2023;23(6):596-601.
130. Yildizdas HY, Erdem B, Karahan DY, Ozlu F, Sertdemir Y. Effect of whole body massage on pain scores of neonates during venous puncture and comparison with oral dextrose and Kangaroo care, a randomized controlled evaluator-blind clinical study. Journal of Perinatology. 2023;43(5):590-4.
131. Yilmaz F, Kucukoglu S, Aytekin Ozdemir A, Ogul T, Aski N. The Effect of Kangaroo Mother Care, Provided in the Early Postpartum Period, on the Breastfeeding Self-Efficacy Level of Mothers and the Perceived Insufficient Milk Supply. Journal of Perinatal & Neonatal Nursing. 2020;34(1):80-7.
132. Zhang B, Duan Z, Zhao Y, Williams S, Wall S, Huang L, et al. Intermittent kangaroo mother care and the practice of breastfeeding late preterm infants: results from four hospitals in different provinces of China. International Breastfeeding Journal. 2020;15(1):64.
133. Zhang X, Wang X, Juan J, Yang H, Sobel HL, Li Z, et al. Association of duration of skin-to-skin contact after cesarean delivery in China: a superiority, multicentric randomized controlled trial. American Journal of Obstetrics and Gynecology MFM. 2023;5(8) (no pagination).
134. Zheng Y, Xia Y, Ye W, Zheng C. The Effect of Skin-to-Skin Contact on Postoperative Depression and Physical Recovery of Parturients after Cesarean Section in Obstetrics and Gynecology Department. Computational & Mathematical Methods in Medicine. 2022;2022:9927805.
135. Zhu LB, Xu YH, Li JF, Hu X, Lu CY, Li RL, et al. The Effect of Kangaroo Mother Care After Duodenal Obstruction in Neonates. Frontiers in Surgery. 2022;9:813052.
136. Zou Y, Li Y, Jiang M, Liu X. Effect of early skin-to-skin contact after vaginal delivery on pain during perineal wound suturing: A randomized controlled trial. Journal of Obstetrics and Gynaecology Research. 2022;48(3):729-38.
137. 丁磊, 单春剑, 王义婷, 骆莎莎. 袋鼠式护理对足月新生儿母乳喂养及行为状态的影响. 中国实用护理杂志. 2018;34(24):1877-82.
138. 何力, 黄朝梅, 刘丹, 邵巧仪, 武兆磊, 贾晓琴. 袋鼠式护理方案改善早产儿经口喂养的效果评价. 蚌埠医学院学报. 2019;44(11):1567-9.
139. 刘艳红, 李胜玲, 崔慧敏, 王晓燕, 孙彩霞. 指导无创机械通气早产儿母亲实施袋鼠式护理对其亲职压力和育儿胜任感的影响. 中华护理杂志. 2022;57(22):2700-7.

140. 周佩艳，罗飞翔. 低体重早产儿呼吸窘迫综合征无创通气治疗期间实施母亲参与袋鼠式照护的临床观察. 中华全科医学. 2023;21(11):1888-90+911.
141. 张燕，卢碧君，沈阳，王瑞钗. 袋鼠式护理对喂养不耐受的极低出生体重早产儿生长发育及母亲护理满意度的影响. 广州医科大学学报. 2019(2).
142. 李润杰，朱丽，张红艳，马红. 基于自然生物角度的袋鼠式护理模式应用于剖宫产初产妇研究. 川北医学院学报. 2021;36(2):261-4.
143. 李玉，李胜玲，孙彩霞，刘艳红，费英山，高丹丹. 袋鼠式护理对无创机械通气早产儿呼吸功能的影响. 护理学杂志. 2021;36(6):11-4.
144. 毕叶，官祥丽. "袋鼠式护理"对早产儿神经行为及体格发育的影响. 解放军护理杂志. 2017;34(17):12-6.
145. 熊小云，谢小华，杨传忠，孙盼盼，陈丽莲，张剑，et al. 无创辅助通气超未成熟儿应用袋鼠护理改善呼吸功能的效果. 南昌大学学报·医学版. 2019;59(3).
146. 熊小云，谢小华，杨传忠，等. 袋鼠式护理改善无创辅助通气超未成熟儿喂养的效果评价. 护理学杂志. 2019;34(15):1-4.
147. 牛晓芬，和丽. 袋鼠式护理对剖宫产初产妇母乳喂养及新生儿黄疸的影响. 中华健康管理学杂志. 2019(06):485-6-7-8-9.
148. 王瑜. 袋鼠式护理对早产儿脑功能、行为神经发育、反复操作性疼痛的影响 [硕士]: 西南医科大学; 2020.
149. 甘桂芬，蔡园. 袋鼠式护理对出生体质量低于2000g早产儿住院期间生长发育和临床疗效指标的影响. 中国中西医结合儿科学. 2018;10(6).
150. 田鸾英，李海燕，王箴. 早期皮肤接触对剖宫产产妇心理状态及母乳喂养的影响：前瞻性随机对照试验. 中华围产医学杂志. 2020(02):105-10.
151. 盖丽，姜红，范玲. 早产儿母亲与父亲实施袋鼠式护理的非劣效性研究. 中国实用护理杂志. 2021;37(20):1568-72.
152. 翟佳，何婧，梅花，肖桂华. 袋鼠式护理在早产儿临床护理中的效果分析. 实用医院临床杂志. 2018;15(1).
153. 翟聪利，孙慧娜，毛竹香，等. 母婴皮肤接触持续时间对新生儿影响的研究. 中华护理杂志. 2018;53(12):1419-23.
154. 胡建新，乐云，刘明秀，李萍. 袋鼠式护理对缓减足月儿疫苗注射所致疼痛的效果分析. 重庆医学. 2016;45(20):2869-71.
155. 谢诺，李娜，裴会，吕一男，白晨. 不同持续时间的早期母婴皮肤接触对新生儿行为状态和母乳喂养的影响. 中国医师杂志. 2018;20(12).
156. 郭路，叶朝，张菁，穆莉萍，冯玉华，刘莹, et al. 实施袋鼠式护理对产妇泌乳、子宫复旧及新生儿疼痛的影响研究. 中国实用护理杂志. 2020;36(18):1383-8.
157. 陆丹琼，王金燕，余霞，王金雅，沈军英，王素萍. 袋鼠式护理对新生儿重症监护室窒息患儿的神经行为影响分析. 中国实用护理杂志. 2020(12):909-10-11-12.
158.陈允蒙，林艳琼，陈韦静. 剖宫产术后袋鼠式护理对初产父亲角色适应及主观幸福感的影响. 中华健康管理学杂志. 2023;17(6):418-23.
159. 鲁萍，厉建英，周迎春，夏芳琴，吕俊英，王建平. 袋鼠式护理在极低出生体重儿中的应用. 中华全科医学. 2022;20(03):523-6.
160. 黄艳萍，刘军，段志英，程媛媛，赵梦. 早期母婴皮肤接触对新生儿母乳喂养的影响. 中华现代护理杂志. 2018;24(30):3684-7.
161. 黄蓉，万宏伟，杨巾夏. 早期持续皮肤接触促进初产妇母乳喂养启动的研究. 中华现代护理杂志. 2016;22(13):1787-91.

162. 黄麒榕, 黄棋, 李彦伶, 唐文豪, 黄艳芳, 董文斌, et al. 袋鼠式护理对早产儿胃肠道功能的影响. 成都医学院学报. 2021;16(06):763-7.
163. NCT02189746, Zambia, 2014, Kangaroo Mother Care to Prevent Hypothermia in Preterm Infants
164. NCT02189759, Zambia, 2014, Kangaroo Mother Care to Prevent Hypothermia in Term Infants
165. NCT02473055, USA, 2015, Kangaroo Care and Premature Infant Sleep
166. NCT02631343, India, 2015, Community Kangaroo Mother Care for Improving Child Survival and Brain Development in Low Birth Weight Newborns
167. NCT02653534, India, 2016, Impact of Promoting Community Initiated Kangaroo Mother Care (KMC) for Low Birth Weight Infants
168. NCT02811432, Uganda, 2016, Kangaroo Mother Care Before Stabilisation Amongst Low Birth Weight Neonates in Africa
169. NCT02849665, Brazil, 2016, Immediate Effect of Kangaroo Position in Electromyographic Activity and Microcirculation of Newborn Preterm
170. NCT02919540, Jordan, 2016, Effect of "Kangaroo Mother Care" on Premature Infants Physiological Outcomes
171. NCT02998463, United Kingdom, 2016, Facilitating Skin-to-Skin Contact In the Postnatal Period
172. NCT03023267, Israel, 2017, The Contribution of Parent-infant Interaction While Singing During Kangaroo Care, on Preterm-infants' Autonomic Stability and Parental Anxiety Reduction
173. NCT03141736, Zambia, 2017, Kangaroo Mother Care With Plastic Bag
174. NCT03200925, United States, 2017, The Effect of Video Education on Skin-to-Skin at the Time of Delivery
175. NCT03521310, Norway, 2018, Immediate Parent -Infant Skin-to-Skin Study (IPISTOSS)
176. NCT03545204, Pakistan, 2018, Implementation Research of Kangaroo Mother Care in Rural Pakistan. (KMC)
177. NCT03555981, Gambia, 2018, Early Kangaroo Mother Care in Gambian Hospitalised Unstable Neonates (eKMC)
178. NCT03574350, Colombia, 2018, Effect on Early Somatic Growth of a Daily Kinesthetic Stimulation on Preterm Infants (KISONKAP)
179. NCT03611088, Brazil, 2018, Effect of Kangaroo Position on Electromyographic Activity, Macrocirculation and Microcirculation of Preterm Newborns
180. NCT03740594, Egypt, 2018, Effect of KMC on Neurobehavior of Preterm Neonates
181. NCT03745963, Canada, 2018, The Influence of Skin-to-skin Contact on Cortical Activity During Painful Procedures on Preterm Infants in the NICU (iCAPmini)
182. NCT03795454, Finland, 2019, Can Singing Kangaroo Improve Outcome of Preterm Infants (SingKang)
183. NCT03990116, Spain, 2019, Efficacy of Lateral Kangaroo Care in Hemodynamic Stabilization of Premature Infant. (Cangulat)
184. NCT04252547, Turkey, 2020, Effect of Kangaroo Care on Test Weighing
185. NCT04364204, Ghana, 2020, Hypothermia Prevention in Low Birthweight and Preterm Infants
186. NCT04506541, Turkey, 2020, The Effects Of KC On Exclusively Breastfeeding And Baby's Growth And Development According To Attachment Theory
187. NCT04619459, Turkey, 2020, The Effect of Kangaroo Mother Care Applied to the Healthy Newborns on Breastfeeding
188. NCT04696770, Canada, 2021, Mindful Kangaroo Care: Mindfulness Intervention for Mothers

During Skin-to-skin Care

189. NCT04725435, Turkey, 2021, Evaluation of Cerebral Oxygenation Results in Premature Infant
190. NCT05491265, Pakistan, 2022, Impact of Intermittent Kangaroo Mother Care Versus Conventional Care Method on Vital Sign and Arterial Oxygen Saturation in Preterm Hospitalized in Neonatal Intensive Care Unit
191. NCT04881071, Spain, 2021, Efficacy of a Scarf to Facilitate Mother-newborn Contact Designed to Facilitate Skin-to-skin Contact (MOKA)
192. NCT04967118, Finland, 2021, Neonatal Pain Management and Pain Monitoring Using New Methods
193. NCT05001334, Turkey, 2021, Effect of Kangaroo Care on Phyiological Weight Loss in Term Newborns
194. NCT05165004, Egypt, 2021, Effect of Kangaroo Mother Care Versus Hammock Positioning
195. NCT05292924, Mexico, 2022, Early Versus Inmediately Skin to Skin Contact in Cesarean Section
196. NCT05337215, Turkey, 2022, The Effect of Postpartum Kangaroo Father Care on Paternal Attachment (kangaroocare)
197. NCT05492747, Turkey, 2022, Kangaroo Care an EffectonTransitionTime From Gavage Feeding (KangarooCare)
198. NCT05607706, Turkey, 2022, Effect of Maternal Odor and Kangaroo Care on Serum Cortisol and Comfort Levels in Preterm Infants: a Randomised Trial
199. NCT05640297, Bangladesh, 2022, Impact of Kangaroo Mother Care Plus Massage Therapy on Growth of Preterm Low Birth Weight Infants at Discharge
200. NCT05657886, Turkey, 2022, Half Swaddle and Kangaroo Care in Preterm on Breastfeeding, Infant Attachment, Sleep Quality and Depression of Mothers
201. NCT05700552, Turkey, 2023, Skin-to-skin Contact for Newborn First Injection Pain
202. NCT05807191, Turkey, 2023, The Effect of Using Clinical Guidelines on Kangaroo Care on Newborn and Maternal Outcomes
203. NCT05820386, France, 2023, Skin-to-skin Contact During the Transfer From the Delivery Room to the Neonatal Intensive Care Unit: Impact on Very Preterm Infants and Their Parents (TRANSPAPNEO)
204. NCT05991648, Colombia, 2023, Effect of Kangaroo Care on Heart Rate Variability in Late-onset Neonatal Sepsis
205. NCT05993442, Italy, 2023, Optimising Kangaroo Care to Reduce Neonatal Severe Infection/Sepsis and Resistant Bacterial Colonisation Among High-risk Infants in Neonatal Intensive Care. (NeoDeco)
206. NCT06047470, United States, 2023, Macronutrients in Lactating NICU Parents - Impact of Kangaroo Care
207. NCT06077409, Turkey, 2023, The Effect of Skin-to-Skin Contact and Gentle Touch Method Applied During Blood Collection on Early Detection of Pain and Physiological Parameters
208. NCT06130488, Pakistan, 2023, Implementation at Scale and Evaluation of KMC (KMC)
209. NCT06154148, Belgium, 2023, Comparison of Two Different Skin-to-skin Contact Techniques: Sustained Diagonal Flexion vs. Traditional
210. NCT06195410, Turkey, 2023, The Effect of Mother and Father Kangaroo Care on Newborn and Parent Outcomes in Premature Infants
211. NCT06196502, Turkey, 2023, The Effect of Gentle Human Touch and Kangaroo Care on Pain and

Crying Physiological Parameters During Hepatitis-B Vaccination in a Newborn

212. NCT06256406, Italy, 2024, Effects of Confortable Environment, Kangaroo Care and Music Therapy in Newborns in Neonatal Intensive Care Unit (PRE-CARE)
213. NCT06266884, Turkey, 2024, The Effect of Kangaroo Care on Father-Baby Attachment
214. NCT06277869, Bangladesh, 2024, Effectiveness Trial of Thermal Jacket
215. NCT06278441, Turkey, 2024, The Effect of White Noise and Kangaroo Care Practices on Stress Parameters in Newborns With Heel Lancing
216. NCT06320587, Turkey, 2024, The Effect of Kangaroo Care on Parents' Perceived Parenting Self-Efficacy, Infant Attachment and Newborn Vital Signs
217. NCT06338410, Pakistan, 2024, Effect of Kangaroo Mother Care on Oxidative Stress and Bonding (KMC)
218. NCT06348316, Turkey, 2024, Early Half Swaddling and Kangaroo Care Practices on Maternal Sleep Quality and Postpartum Depression in Term Babies
219. NCT06350071, Turkey, 2024, Effect of Kangaroo Care and Swaddling Methods on Pain Level and Crying Time During Heel Blood Collection
220. NCT06380231, Turkey, 2024, Skin-to-skin Contact Between Premature Infant and Mother
221. NCT06400446, Turkey, 2024, The Effect of Kangaroo Care After Cardiac Surgery
222. NCT06436404, Turkey, 2024, Hammock Position and Kangaroo Care
223. ACTRN12614000218695, Philippines, 2014, Kangaroo Mother Care and Its Lactogenic Effects
224. ACTRN12618001880235, Ghana;India;Nigeria;Malawi;Tanzania, 2018, Effect of immediate Kangaroo Mother Care on neonatal mortality for mothers and babies
225. ChiCTR1900026363, China, 2019, Effect of kangaroo mother care on neurodevelopmental outcomes and repeated procedural pain in premature infants
226. ChiCTR2000029746, China, 2020, Effect of early skin-to-skin contact (kangaroo mother care) on pain of delivery mothers with episiotomy or perineal laceration repair: a randomized controlled trial
227. ChiCTR2000033636, China, 2020, randomized controlled study on the effect of kangaroo mother care on gastrointestinal function of premature infants
228. ChiCTR2200057854, China, 2022, The clinical application of kangaroo mother care in neonates with duodenal obstruction after surgery
229. ChiCTR2400080924, China, 2024, The effect of Kangaroo mother care on the Postpartum depression emotion
230. ChiCTR-IOR-17012745, China, 2017, ", Early Father-Infant Skin to Skin Contact and the impact on Neurodevelopment Outcomes of Moderately Preterm Infants in China"
231. CTRI/2014/12/005309, India, 2014, Provision of respiratory support along with Kangaroo Mother care
232. CTRI/2016/01/006524, India, 2016, Effect of Community Initiated Kangaroo Mother Care on babies with Low Birth Weight
233. CTRI/2016/02/006653, India, 2016, ", Community Kangaroo Mother Care for improving child survival and brain development in Low Birth Weight newborns"
234. CTRI/2016/06/007028, India, 2016, Comparison of Music Therapy with Kangaroo Mother Care for pain reduction in premature babies
235. CTRI/2017/02/007824, India, 2017, Comparing Kangaroo Mother care and Oral Sucrose in management of Pain in neonates
236. CTRI/2017/04/008395, India, 2017, Effect of music therapy on breast milk production in mothers

practicing kangaroo mother care

237. CTRI/2017/04/008430, India, 2021, Infections and growth in low birth weight infants: Role of community initiated kangaroo mother care on breast milk and underlying biological pathways
238. CTRI/2017/07/009006, India, 2021, A trial to study the effect of kangaroo mother care (KMC) on duration of phototherapy in neonatal jaundice
239. CTRI/2017/10/010114, India, 2017, What is the effect of community initiated Kangaroo Mother Care on mortality across socioeconomic status and household expenditure on care seeking for enrolled babies
240. CTRI/2017/11/010612, India, 2017, Comparing Kangaroo Mother Care and Oral Dextrose 50% in reducing Pain in Preterm newborn undergoing Heel Prick
241. CTRI/2017/12/010740, India, 2017, To compare physiological responses during kangaroo care by fathers and mothers stable in preterm neonates
242. CTRI/2018/01/011348, India, 2018, ", Kangaroo Care for pain reduction. Mothers versus NonMothers"
243. CTRI/2018/02/011950, India, 2018, A Randomized controlled trial to assess the effectiveness of an interventional package in mothers and preterm neonates on preterm neonatal feeding in terms of exclusive breast milk feeding at discharge
244. CTRI/2018/05/014006, India, 2018, Comparing Kangaroo Mother Care and Sucrose in Reducing Pain in Preterm Neonates on Heel Prick
245. CTRI/2018/08/015154, India, 2018, Monitoring Hypothermia in neonates via bracelet to improve Kangaroo Mother Care
246. CTRI/2018/08/015369, India, 2018, Immediate skin to skin contact of the baby with mother or the surrogate
247. CTRI/2019/05/019428, India, 2019, How much time of Kangaroo Mother care is enough to prevent pain during procedure in a neonate
248. CTRI/2019/11/021899, India, 2019, Immediate KMC neurodevelopmental follow-up study
249. CTRI/2020/01/022984, India, 2020, Randomized controlled trial of pain control in neonate and mother with immediate skin to skin care
250. CTRI/2020/11/029050, India, 2020, Transport of Newborns We will check the utility of KMC for safe and effective transport back home
251. CTRI/2021/06/033970, India, 2021, A study to check whether music combined with standard Kangaroo Mother Care help in improving neurodevelopmental outcome of preterm infants.
252. CTRI/2021/10/037264, India, 2021, Study of effect of early kangaroo mother care on babies with birth weight less than 1500grams
253. CTRI/2023/03/050420, India, 2023, Implementing Kangaroo Mother Care soon after birth in district hospitals
254. CTRI/2023/04/052060, India, 2023, ", Impact of continuous kangaroo mother care initiated immediately after birth(iKMC)"
255. CTRI/2023/05/052498, India, 2023, A randomized control trial to know the effect of Skin to Skin versus Cloth to Cloth on compliance to Kangaroo mother care
256. CTRI/2023/05/052960, India, 2023, Assessment of parent compliance to kangaroo mother care in low birth weight babies between 1.5-2.5kg using the BEMPU device and KANGA sling as compared to the conventional care-A theee arm hospital based randomised control Trial
257. CTRI/2023/06/053402, India, 2023, Comparison of pain relieving effect of kangaroo Mother care

and expressed breast milk administration during removal of adhesive tape in low-birth weight neonate

258. CTRI/2023/07/055853, India, 2023, ", study effect of kangaroo mother care v/s conventional care on clinical outcomes in hemodynamically stable low birth weight babies (less than or equal to 1800 grams)"
259. CTRI/2023/08/057053, India, 2023, ", Integrating Kangaroo mother care for small babies immediately after birth into routine health services in Uttar Pradesh"
260. CTRI/2024/01/062057, India, 2024, Impact of early prolonged Kangaroo Mother Care on normal birth weight infants
261. CTRI/2024/04/065577, India, 2024, Benefits of coconut oil application in preterm receiving KMC
262. CTRI/2024/05/067516, India, 2024, To know which is best among two methods of care(yaksons touch and kangaroo mother care) during light treatment on babies with jaundice
263. IRCT20090810002324N19, Iran (Islamic Republic of), 2022, The effect of two-different time methods of kangaroo care on anxiety, depression, and general health in mothers of premature infants.
264. IRCT2013052813489N1, Iran (Islamic Republic of), 2014, The Effect of Kangaroo Mother Care in Colicky Infants
265. IRCT201306082324N11, Iran (Islamic Republic of), 2014, The effect of kangaroo Mother Care (KMC) by mothers and telephone advice on anthropometric indexes and some of the behavioral features in preterm newborns
266. IRCT2014052117787N1, Iran (Islamic Republic of), 2014, The Effect of Kangaroo Mother Care on Maternal General Health
267. IRCT2014120217972N4, Iran (Islamic Republic of), 2015, Paternal vs Maternal Kangaroo and routine care for pain relief in preterm neonates after heel lancet procedure
268. IRCT2015030310426N7, Iran (Islamic Republic of), 2015, Effect of kangaroo mother care on premature infants_ physiological indices and breastfeeding in the Fatemieh Hospital in Hamadan in 2015
269. IRCT20150424021917N10, Iran (Islamic Republic of), 2020, The effect of kangaroo care by mother and surrogate on nutritional behavior of preterm neonates : A randomised controlled clinical trial
270. IRCT201505142639N16, Iran (Islamic Republic of), 2015, Efficacy of kangaroo mother care, breastfeeding and swaddling on BCG vaccine pain score in neonates
271. IRCT201505231760N40, Iran (Islamic Republic of), 2015, The effect of Kangaroo Mother Care (KMC) on serum bilirubin level in full term newborns with hyperbilirubinemia admitted for phototherapy
272. IRCT2015052914251N3, Iran (Islamic Republic of), 2015, Effect of neonate's massage and kangaroo mother care on neonates' pain
273. IRCT201506064617N12, Iran (Islamic Republic of), 2015, Effects of kangaroo mother care on the level of bilirubin in the newborn after cesarean and maternal satisfaction from care after birth
274. IRCT2017013032307N1, Iran (Islamic Republic of), 2017, The effect of kangaroo mother care on maternal fatigue
275. IRCT20171230038142N6, Iran (Islamic Republic of), 2019, The effect of postpartum Kangaroo Mother Care on depression in mothers
276. IRCT20180429039464N5, Iran (Islamic Republic of), 2021, The Effect of Kangaroo Mother Care

on Sleep Quality of Mothers With Preterm infants In Neonatal Intensive Care Unit
277. IRCT20180519039709N1, Iran (Islamic Republic of), 2018, Comparision the effect of kangaroo mother care (KMC) with Field massage on serum bilirubin level in full term newborns with hyperbilirubinemia
278. IRCT20181024041451N1, Iran (Islamic Republic of), 2019, The effects of Kangaroo Mother Care education intervention on the Premature Infants
279. IRCT20181121041718N1, Iran (Islamic Republic of), 2019, The effect of Home Visit Program based on the Continued kangaroo Mother Care on Maternal Resiliency and Development of Premature Infant
280. IRCT20190703044082N4, Iran (Islamic Republic of), 2021, The effect of kangaroo mother care by role-playing method on resilience and breastfeeding self-efficacy in mothers of premature neonates, Fatemieh hospitalized in neonatal intensive care unit, Hamadan, 202
281. IRCT20190703044082N5, Iran (Islamic Republic of), 2021, The effect of kangaroo mother care by role-playing method on mother-infant attachment and sleep quality in mothers of premature neonates, Fatemieh hospitalized in neonatal intensive care unit, Hamadan, 2021
282. IRCT20191215045749N1, Iran (Islamic Republic of), 2020, The effect of oral sucrose on Premature Newborns pain ";" The effect of kangaroo Mother care on Premature Newborns pain; "
283. IRCT20201130049544N1, Iran (Islamic Republic of), 2021, Investigating the effects of Kangaroo care on Delirium in Neonates under Non-Invasive mechanical Ventilation
284. IRCT20210109049974N1, Iran (Islamic Republic of), 2023, Comparison of kangaroo mother care and tactile-kinesthetic stimulation on weight gain of late preterm infants in hospitalized in neonatal intensive care units.
285. IRCT20210501051142N3, Iran (Islamic Republic of), 2022, Comparison of three methods of foot massage, kangaroo care and standard care on bilirubin level and physiological indicators
286. IRCT20211225053516N1, Iran (Islamic Republic of), 2022, Comparison of the effect of maternal and grandmother kangaroo infant care on vital signs of premature neonates
287. IRCT20240204060894N1, Iran (Islamic Republic of), 2024, Investigating the effect of kangaroo mother care (KMC) on the vital signs of preterm infants
288. ISRCTN85196891, Mexico, 2023, Comparing methods for weight gain in premature infants
289. PACTR201607001698226, Kenya, 2016, Averting deaths among preterm and low-birth weight neonates using Community Kangaroo Mother Care intervention: A cluster randomized trial
290. PACTR202009770590788, Ethiopia;Ethiopia, 2018, The Effects of Short Duration Kangaroo Mother Care on Neonatal Pain from Adhesive Tape Removal: A Randomized Controlled Trial Among Preterm Neonates Admitted to Tikur Anbessa Specialized and Gandhi Memorial Hospitals in Addis Ababa, Ethiopia
291. PACTR202403567799525, United Republic of Tanzania, 2024, Addressing neonatal mortalities through prolonged Immediate Kangaroo Mother Care in Kibaha Tanzania
292. RBR-2q8bzk, Brazil, 2016, The effect of combining breastfeeding plus skin-to-skin contact on the BCG vaccination induced pain in term infants: a randomized clinical trial

Supplementary Table S4 characteristics of included studies

Author/year	Study type	Sample	Participants	Intervention	Initiation	KMC giver	KMC Duration	Control	Outcomes
WHO Immediate KMC Study Group 2021	RCT	3211	LBWI	immediate KMC	immediate	Mothers	16.9h	conventional care in an incubator or a radiant warmer until their condition stabilized and KMC	mortality, hypothermia, hypoglycemia, suspected sepsis, exclusive breastfeeding rate, satisfaction, maternal depression, hospital stay
Jayaraman D 2017	RCT	160	neonates weighing between 1000 and 1800 g	early KMC	within the first 4 days of life	Mothers	>4h/day	late KMC off respiratory support and off intravenous fluids	breastfeeding outcomes, mortality, morbidity, KMC duration, vital signs, adverse events, weight, length
Khadivzadeh 2017	RCT	114	<2500g	immediate SSC	immediate	Mothers	2h post-birth	routine care	breastfeeding outcomes, oral feeding outcomes, weight
Kollmann 2017	RCT	35	≥37w	early SCC	immediate, within 5 minutes after birth	Mothers	continuously until the back to their room at the delivery suite	late SCC	arterial oxygen saturation (SaO2), HR, maternal cortisol levels, newborn cortisol levels, pain, change in rectal temperatures of newborns
Li 2017	RCT	216	35~40w	KMC group were accepted phototherapy intermittently	During the phototherapy	Mothers	intermittently, 3times/d, 7d	phototherapy	weight, bilirubin, duration of photo therapy

Author/year	Study type	Sample	Participants	Intervention	Initiation	KMC giver	KMC Duration	Control	Outcomes
Mirnia 2017	RCT	45	1200～2500g	SSC	NA	Fathers	0.75h	routine care	cortisol
Marulli 2018	crossover	39	<33w	SSC	NA	Mothers	1.5h	no	cerebral tissue oxygenation, HR, bradycardic events, cerebral hypoxia, cerebral, oxygen, hypoxic events were
Badiee 2018	RCT	50	<37w	KMC	NA	Mothers	1h/d, 7d	standard care	physical disorders, anxiety symptoms and sleep, depression, social health
Adeli 2018	RCT	68	38～42w	kangaroo skin contact	during breastfeeding	Mothers	0.6h	routine care	Maternal attachment behaviors, oxytocin, time to first SSC, breastfeeding outcomes
Deng 2018	RCT	50	32~34w	father-infant SSC	medical stability	Fathers	>1h/d	standard care	anxiety, salivary cortisol, sleep, neuroimaging, neurodevelopmental, bonding, pain, hypothermia, apnea, RR, HR, oxygen saturation, adverse events
Pandita 2018	RCT	61	Term	KMC	during vaccination	Mothers	0.5h	swaddled	pain scores, duration of cry, HR, Oxygen Saturation
Parsa 2018	quasi-experimental study	100	34~36w	daily KMC	NA	Mothers	1h/d, 7d	routine care	body temperature, Oxygen Saturation, HR, RR
Ramani 2018	RCT	203	≥37w	KMC	immediate	mothers	7h	local standard thermoregulation care	axillary temperature, moderate or severe hypothermia, hyperthermia, NICU admission, death, KMC duration

Author/year	Study type	Sample	Participants	Intervention	Initiation	KMC giver	KMC Duration	Control	Outcomes
Ropars 2018	RCT	746	≤2000g	KMC for 24 hours/day (or as long as possible) until they showed behavioral manifestations that they were ready to be separated	NA	mothers	24h, continuous	kept in incubators	General cognitive functioning (IQ), Attentional measures
Sharma 2018	RCT	79	1000~1100g	Kangaroo ward care (KWC):as many hours per day as possible ensuring a minimum of six hours per day.	NA	mothers	>6h	Intermediate Intensive care (IIC)	weight, head circumference, length, feeding method, TPN Duration, duration of intravenous fluids
Shukla 2018	RCT	100	29~36w	SSC was provided at least 10 min before and was continued as per the unit protocol post heel stick procedure	during heel stick procedure	mothers	0.2h	0.2 ml of 24% Sucrose was provided 2 min before the procedure.	HR, oxygen saturation, pain
Soltani 2018	RCT	161	37~42w	1.Breastfeeding; 2. 25% dextrose; 3.KMC; 4.KMC+EMLA	during heel stick procedure	mothers	0.3h	no	pain scores, weight
Campbell-Yeo 2019	RCT	242	≤36w	1.maternal KC, duration of their infant's hospital stay.2.KC+sucrose	clinically stable	mothers	0.3h	Sucrose	pain, adverse events
Huang 2019	RCT	108	37~41w	SSC	after cesarean	Fathers	0.5h	routine care.	duration of crying, HR, anxiety, depression, bonding, temperature, Self-sucking of breast milk, Breastfeeding period
Mazumder 2019	RCT	8402	1500–2250g	community-initiated KMC	stable and feeding	Mothers	24h, continuous	routine care	mortality, time of breastfeed initiation, breastfeeding initiated within 1 h of birth, KMC duration, weight, length, infection, diarrhea, pneumonia, head circumference, morbidity

Author/year	Study type	Sample	Participants	Intervention	Initiation	KMC giver	KMC Duration	Control	Outcomes
Shattnawi 2019	quasi-experimental	89	26～37w	SSC was initiated immediately after NICU admission, for five days	stable	mothers	1-2h/5d, intermittent SSC	standard care	weight gain, sleeping, crying patterns, apnea, length, head circumference
Yilmaz 2019	quasi-experimental	60	2500～4000g	KMC	the first day after giving birth	mothers	0.5h	no intervention	breastfeeding self-efficacy
Coşkun 2019	RCT	84	27~36w	KC	NA	mothers	0.3h/d, 3w	standard care	Mother's milk amount, parental stressor
Recife 2020	RCT	44	28～37w	Kangaroo Unit	clinically stable	mothers	1-2h	no intervention	electromyographic activity
El-Farrash 2020	RCT	120	31~35w	KC for 60 min daily, KC for 120 min daily	the first 24 h of life	mothers	1-2h	conventional care	HR, RR, temperature , oxygen saturation, neurobehavioral , salivary cortisol, oral feeding outcomes, breastfeeding outcomes
Forde 2020	RCT	51	24～36w	1 hr of KMC on Day 3 of life	on day 3 of life	mothers	1h	had only incubator care onDay3	HR, perfusion index (PI), temperature, abdominal tissue saturation, illness severity, stress-related biochemical markers
Hardin 2020	RCT	33	29~38w	recommended-use KC (> 1 h. per day for 6 weeks; low-use KC (< 1 h per day for 6 weeks)	NA	mothers	1h, 6w	no intervention	Cortisol, neurodevelopment, oxytocin
Karimi 2020	RCT	100	34～36w	KMC for an hour per day for seven days.	stable	mothers	1h,7d	conventional care (incubator)	HR, RR, temperature
Hucklenbruch-Rother 2020	RCT	88	25～32w	delivery room-SSC, 45 min after birth and was performed for 60 min continuously supervised	45 min after birth	mothers	1h, mothers	visual contact	mother-child interaction, cortisol, gene expression

Author/year	Study type	Sample	Participants	Intervention	Initiation	KMC giver	KMC Duration	Control	Outcomes
Liao 2020	RCT	78	37~42w	KMC, for 3 consecutive days after birth, the two groups of newborns were given corresponding nursing care twice a day for 1 hour each time	for 3 consecutive days after birth	Mothers	1h	routine care	the score of NIPS, sleep quality, weight, height, head circumference, breastfeeding success rate
Linner 2020	RCT	55	28~33w	SSC was immediately	immediately	parent	1h	routine care	body temperature, KMC duration, adverse events, feeding at discharge, expressed breast milk
Mansoori 2020	quasi-experimental	50	28～33w	KMC: two sessions of milking were performed during KMC	NA	Mothers	NA	the KMC twice a day	Breast milk expression
Mehler 2020	RCT	88	25~32w	DR-SSC, started approximately 45 minutes after birth and was performed for 60 minutes continuously	45 minutes after birth	Mothers	1h	5 minutes of VC	the quality of mother-child interaction, reactivity of HPA axis, Depressive symptoms, bonding
Nimbalkar 2020	crossover	100	28~36w	SSC was given 15-min before first heel-stick	during heel stick procedure	mothers or fathers	0.3h	sucrose was given 2-min before second heel-stick.	pain scores
Rehman 2020	RCT	140	neonates	Intermittent KMC was applied for one hour at a time every 4-hourly for seven days.	NA	Mothers	6h/d, Intermittent KMC	conventional therapy.	weight gain, length, oral feeding outcomes
Helmer 2020	RCT	31	32~35w	continuous SSC	immediately	Mothers	24h, continuous	intermittent SSC	infants' cortisol reactivity, depression, SSC time, mother–infant interaction

Author/year	Study type	Sample	Participants	Intervention	Initiation	KMC giver	KMC Duration	Control	Outcomes
							SSC		
Sen 2020	RCT	64	32～37w	KC	during heel stick procedure	Mothers	0.3h	oral sucrose	HR, oxygen saturation, pain
Taneja 2020	RCT	552	1500–2250 g	community-initiated KMC, skin-to-skin contact and exclusive breastfeeding	stable	Mothers	24h, continuous SSC	Home Based Post Natal Care (HBPNC) visits	Cognitive, language, motor and socio-emotional outcomes, temperature, maternal depression, maternal sense of competence, mother-infant bonding, number of days and average hours per day), home environment
Yilmaz 2020	quasi-experimental	60	2500～4000g	KMC, 30 minutes. twice in the morning and evening,	the first 3 days after birth	Mothers	1h/d	regular protocols	breastfeeding self-efficacy, breastfeeding outcomes
Zhang 2020	quasi-experimental	844	34~36w	Intermittent KMC	stable	Mothers	1h, Intermittent KMC	No KMC	feeding outcome, breastfeeding outcomes
Agudelo 2021	RCT	297	>37w	Immediate (in the first minute after birth) SSC	Immediate	Mothers	1h	early onset (start exactly at 60 min of life) SSC	percentage of exclusively breastfed infants, duration in months of exclusive breastfeeding, weight loss, hospitalization in the first week of life

Author/year	Study type	Sample	Participants	Intervention	Initiation	KMC giver	KMC Duration	Control	Outcomes
Shukla 2018	RCT	200	28~36w	KMC with music, music, KMC	during heel stick procedure	mothers	0.3h	music therapy	PIPP score
Wang 2021	RCT	79	33~35	2.5 hours/day KMC	NA	Mothers	2.5 hours/day KMC	standard care	physical growth, Breastfeeding feeding outcomes, feeding intolerance, neurobehavioral development
Bi 2017	RCT	98	28~36w	KMC, 30~60min, 2/d, for 14days	stable	Mothers	1-2h,14d	standard care	RR, HR, oxygen saturation, mental development, psychomotor development index (PDI), neurodevelopment, weight, length, head circumference
Huang 2018	RCT	160	>37w	SSC was immediately for 90min	Immediate	Mothers	1.5h	SSC within 30min	the time of feeding cues, oral feeding outcomes, the time of sucking initiation, duration of the first breastfeeding session, breastfeeding initiation rate, breastfeeding completion rate within 90 minutes, exclusive breastfeeding rate before discharge, exclusive breastfeeding rate before discharge
He 2019	RCT	92	28~34w	KMC more than 1h	NA	Mothers	>1h/d	routine care	oral feeding ability, time to reach basic feeding criteria, duration of parenteral nutrition, feeding transition period, Feeding intolerance, apnea
Feng 2019	RCT	100	39~40w	SSC within 5~10min after birth	Immediate	Mothers	0.3-0.6h	routine care	time to first lactation, feeding cues

Author/year	Study type	Sample	Participants	Intervention	Initiation	KMC giver	KMC Duration	Control	Outcomes
Zhang 2019	quasi-experimental	60	1000～1500g	KMC for 1h, 2~3times/d, duration for 1month	NA	Mothers	2-3h, 1m	routine care	feeding intolerance, time of sleep, time to reach full enteral feeding, time to regain normal birth weight, daily milk intake, length of hospital stay, satisfaction, weight, length, head circumference
Xiong 2019	RCT	114	<28w	KMC for 3h, 1 times/d, duration for 2w	stable	mothers or fathers	3h, 2w	routine care	duration of non-invasive ventilation, duration of headbox oxygen therapy, time of oxygen therapy, apnea
Xiong 2019	RCT	123	<1000g/<28w	KMC for 3h, 1 times/d, duration for 2w	stable	mothers or fathers	3h, 2w	routine care	time to reach full enteral feeding, feeding patterns, achieve to full oral feeding, exclusive breastfeeding rate
Niu 2019	RCT	120	>37w	KMC lasts no less than 30min, 3 times/d	after cesarean	Mothers	1.5h	routine care	time to first lactation, first breastfeeding success rate, breastfeeding self-efficacy and knowledge, bilirubin, stool
Wang 2020	RCT	76	31～33w	KMC lasts no less than 1h	stable	Mothers	1h	routine care	sleep, neurodevelopmental outcome, pain, HR, oxygen saturation, cerebral tissue oxygen saturation
Tian 2020	RCT	210	>37w	SSC 1~2h	with 1h after birth	Mothers	1~2h	routine care	time to initiation of lactation, first breastfeeding success rate, breastfeeding self-efficacy and knowledge, depression, exclusive breast-feeding rate
Guo 2020	RCT	200	2500~4000g	KMC， 1h, 2/d, at least 14d	with 0.5h after birth	Mothers	1h, 2/d, at least 14d	routine care	awareness of KMC, time to first lactation, breast engorgement, 48h breast-milk volume, breastfeeding status, pain, uterine involution

Author/year	Study type	Sample	Participants	Intervention	Initiation	KMC giver	KMC Duration	Control	Outcomes
Lu 2020	RCT	76	33～42w	KMC，1h, 3~4/w	NA	mothers or fathers	1h, 3~4/w	routine care	neurodevelopmental, visual impairment, hearing impairment, cerebral palsy, limb functional impairment, weight, length, head circumference
Zhou 2023	RCT	156	28~36w	KMC，3h, 1/d, Continue until the end of non-invasive ventilation	stable	Mothers	3h, 1/d,	routine care	arterial blood gas parameters, duration of non-invasive ventilation, use of PS, use of invasive ventilation, ventilation complication
Chen 2023	RCT	160	37~42w	KMC by father30~40 min, 2 /d，3d	after the newborn returned to the ward.	fathers	1-1.2h, 3d	routine care	father's role, sense of well-being or Happiness
Srinath 2016	cross-over	26	preterm infants, ＜35 weeks，＜ 1500g	kangaroo mother care，60min	stable	mothers or fathers	1h	kangaroo father care，60min	HR, temperature, noninvasive blood pressure, oxygen saturation, Salivary cortisol
Kurt 2020	quasi-experimental	60	preterm infants	KMC，30min，2 times/day，5days	stable	mothers	1h, 5d	routine practice，10min，2 times/day，5days	maternal attachment
Abasalizadeh 2024	RCT	60	preterm infants	KMC，1h/day，1weeks	NA	mothers	1h/day，1weeks	conventional method of care (CMC)	resilience of mothers
Acharya 2014	RCT	126	LBW	KMC, 6h/days，＜4times/days，	stable	mothers	6h/d, Intermittent KMC	babies were adequately clothed, covered and kept with their mother/kept under radiant warmer	average weight gain, temperature, apnea, duration of hospital stay

Author/year	Study type	Sample	Participants	Intervention	Initiation	KMC giver	KMC Duration	Control	Outcomes
Aghdas 2014	RCT	114	full term mothers between 18 and 35 years of age	skin-to-skin contact SSC，at least 2 h	immediate	mothers	>2h	routine care	breastfeeding self-efficacy, success in first breastfeeding, mean time of first breastfeeding initiation
Bera 2014	Quasi-Experimental Study	449	LBW	On the first day, KMC 1 h , on the second day for 2 h and on the third day for 3 h. The duration was increased after that to as long as the mother felt comfortable.	On the first day	mothers	1-3h	standard care	weight, head circumference, length, chest circumference, arm circumference, development
Çaka 2023	RCT	168	Preterm infants	Kangaroo Mother Care，60min，days (min: 3, max: 5)	stable	mothers	1h	standard care	Body temperature, RR, oxygen saturation, HR, weight, time of transition to full enteral feeding, length of hospital stay, feeding intolerance
Chidambaram 2014	crossover	100	preterm neonates	Kangaroo mother care，15min	stable	mothers	0.2h	NA	Premature Infant Pain Profile (PIPP)
Cong 2015	cross-over	28	pre-term infants	Maternal skin-to-skin contact (M-SSC)	stable	mothers or fathers	0.5h	paternal SSC	salivary oxytocin assay, salivary cortisol, parental anxiety
Dehghani 2015	RCT	53	more than 1800g，32 weeks and above	Kangaroo mother care，3 consecutive days and each time for 1-h	NA	Mothers	1-h	conventional incubator care	temperature average, arterial oxygen saturation rate, average HRs, RR
Eckermann 2024	RCT	127	full-term infants	skin-to-skin contact，1h/ day SSC from birth to five weeks of age	immediately	Mothers	1h/ day	care-as-usual	alpha diversity, microbiota volatility, microbiota age, gut brain module abundances

Author/year	Study type	Sample	Participants	Intervention	Initiation	KMC giver	KMC Duration	Control	Outcomes
Eksirinimit 2023	quasi-experimental study	64	premature infants	KC 1 h/day for three consecutive days	NA	mothers	1 h/day	usual care	temperature
El Sehmawy 2023	RCT	400	fullterm babies	ssc, 1 to 3 h/day during the first 12 postpartum weeks, starting from the first day	starting from the first day	mothers	1-3 h/day	usual mother–infant care	baby weight, hours of baby sleep, breastfeeding frequency, anxiety, depression, Pittsburg sleep quality index, pain, wound healing
Erduran 2023	RCT	60	preterm infants	intermittent kangaroo care，30min/day，10days	NA	mothers	0.5h, 10days	routine care	maternal attachment, depression
Kristoffersen 2016	RCT	136	28-31, >1000g	Early skin-to-skin contact	Within 2 hours after birth	mothers	NA	incubator	Bayley-III, temperature, complications to prematurity (intraventricular hemorrhage or periventricular leukomalacia seizures, NEC,anxiety, motor function, social emotional function, and BPD
Jajoo 2024	cross-over	70	Stable preterm neonates	kangaroo mother care，30min	during heel stick procedure	Mothers	0.5h	under control (bassinet) conditions	pain, HR, oxygen saturation
Kristoffersen 2023	RCT	108	preterm infants born at GA 28–31 weeks with birth weight >1000 g	2 hours of early skin-to-skin contact	early SSC initiated shortly after birth in the delivery	Mothers	2h	standard care (SC)	neurodevelopmental outcome, hypothermia, the need for mechanical ventilation, HR, RR, oxygen saturation

Author/year	Study type	Sample	Participants	Intervention	Initiation	KMC giver	KMC Duration	Control	Outcomes
Lamy Filho 2015	RCT	102	birth weight of 1300 to 1800 g	SSC，60min，2 times/day，7days	NA	Mothers	1-2h, 7d	routine care	colonization status of newborns' nostrils after 7 days of intervention
Linnér 2023	RCT	3211	LBW	immediate kangaroo mother care，continued until discharge	immediate	Mothers	continuously	conventional care，	HR, RR, oxygen saturation
Lode-Kolz 2023	RCT	91	very preterm infants	immediate skin-to-skin contact,first 6 postnatal hours	immediate	mothers or fathers	5h	on a resuscitative or in an incubator，intermittent SSC was initiated after the 6 first hours	Mean axillary temperature
Manzoor 2023	RCT	100	LBW	kin to skin contact (SSC)	NA	mothers	NA	Conventional Method of Care (CMC)	exclusive breast feeding
Mörelius 2015	RCT	32	late preterm infants	continuous SSC	immediately	Mothers/fathers	continuous,19 h/d	standard care	Salivary cortisol, parental stress, depression-parental, breastfeeding outcome
Mosayebi 2014	crossover	64	Preterm Newborns	KMC at an angle of approximately 60º in skin-to-skin contact with their mother	during heel stick procedure	Mothers	0.3h	incubator care	pain
Nimbalkar 2023	RCT	152	LBW	Neonates in the study group received KMC while transported from the hospital to their homes	during transport	Mothers	NA	they wore cotton cloth (jabla), diapers, socks, cap, and gloves and were covered with a warm blanket during the transport	hypothermia

Author/year	Study type	Sample	Participants	Intervention	Initiation	KMC giver	KMC Duration	Control	Outcomes
Patel 2022	RCT	100	Late preterm and full-term newborns	skin-to-skin care (SSC),Vitamin K injection was given intramuscularly at the anterolateral aspect of the newborn's thigh 30 min after birth while SSC continued	immediate	Mothers	0.5h	After 30 min of immediate SSC, the newborn was shifted under radiant warmer	NIPS score
Pujara 2023	crossover	23	LBW	Kangaroo Mother Care,2h/times，4 times/day	on day 1 of enrollment	Mothers	8h	CCC was provided by the mother wearing her routine cloths and the dressed newborn was kept prone on mother's clothed chest secured with the binder	Mean temperature difference at different time points, hypothermia, hyperthermia
Rangey 2014	A quasi-experimenta l study	30	Low Birth Weight Preterm Infants	KMC 15min,3 times/day,5 days	stable	Mothers	0.75h,5d	Massage therapy，received 15 minutes of MT thrice daily for 5 days	Body weight, length of hospital stay
Rheinheimer 2023	RCT	116	full-term infant	skin-to-skin contact (SSC)，for 1 hr a day for 5 weeks	immediately	Mothers	1h/d, 5w	care-as-usual (CAU)	Children's executive functioning, Children's problem behavior
Rheinheimer 2022	RCT	116	full-term infants	skin-to-skin contact (SSC)，for 1 hr a day for 5 weeks	immediately	Mothers	1h/d, 5w	care-as-usual (CAU)	Infant cortisol reactions, infant behavioral reactions, quality of maternal caregiving, mother–infant adrenocortical synchrony

Author/year	Study type	Sample	Participants	Intervention	Initiation	KMC giver	KMC Duration	Control	Outcomes
Samra 2015	RCT	40	Late-Preterm Infants	skin-to-skin care (SSC)，3 times/ week at 50 minutes per session throughout NICU stay	immediately	Mothers	0.8h	held the baby wrapped in a blanket and were also encouraged to hold their infant for a minimum of 3 times a week at 50 minutes per session throughout NICU stay	Maternal stress
Singh 2023	RCT	100	Neonates born 33-36 weeks	skin-to-skin contact (SSC),60min	Immediate	Mothers	1h	care under a radiant warmer	the stability of the cardiorespiratory system, HR, RR, SpO2, axillary temperature, blood sugar, breastfeeding outcomes, the duration of the respiratory support, the duration of NICU admission, the duration of hospital stay
Singh 2024	RCT	80	LBWI	Kangaroo mother care (KMC)，1h	stable	Mothers	1h	conventional method of care (CMC)，were managed under radiant warmers	oxygen saturation, weight, body temperature, hypothermia episodes
Sinha 2022	RCT	200	LBW	community-initiated Kangaroo Mother Care	NA	Mothers	continuously	usual care	the concentration of the individual fecal biomarkers, enteric Enteropathy score
Srivastava 2022	RCT	80	LBW	kangaroo mother care (KMC),60min	during procedure	Mothers	1h	no KMC	pain

Author/year	Study type	Sample	Participants	Intervention	Initiation	KMC giver	KMC Duration	Control	Outcomes
Srivastava 2014	RCT	298	in term babies	early skin-to-skin contact (SSC)，naked neonate prone on mother's chest immediately or soon after delivery	immediately	Mothers	>2h	standard care，after drying and weighing, the baby was clothed, wrapped in a sheet and a blanket and placed next to her/his mother	IBFAT score, infants exclusively breastfed, axillary temperature, weight loss at discharge
Tumukunde 2024	RCT	2221	All liveborn neonates aged younger than 48 h and weighing 700–2000 g	KMC ≥1 h per session	initiated before clinical stabilization	caregiver	≥1 h	standard care	mortality, hypothermia, Time to stabilization, time to death, time to exclusive breastmilk feeding, duration of hospital admission, readmission frequency, daily weight gain, women's wellbeing, maternal responsiveness
Gündüz 2023	RCT	84	newborns were 37 and 40 weeks, being healthy	skin-to-skin contact (SSC)	immediately	Mothers	NA	routine hospital procedures	the placental separation time, breastfeeding
Türker 2022	RCT	70	full-term by vaginal delivery	kangaroo mother care	during vaccinations	Mothers	0.2h	breastfeeding (BF)	HR, oxygen saturation, duration of crying, pain

Author/year	Study type	Sample	Participants	Intervention	Initiation	KMC giver	KMC Duration	Control	Outcomes
Veeraiah 2023	Quasi-Experimental Study	76	Preterm neonates	kangaroo mother care（given KMC for one hour instead of being wrapped in a cloth）	after bath	Mothers	1h	standard care（the neonate was wrapped with a clean cloth）	Axillary temperature
Wang 2022	RCT	65	preterm infants	KMC for at least 1 h / day,14 consecutive days	NA	Mothers	>1h/d， 14d	traditional care (TC)，cared for under radiant warmers or in the incubators and treated with routine nursing procedures	brain function, the neonatal behavioral neurological
Wang 2023	RCT	73	preterm infants	KMC 30 min before the heel stick until the end of the recovery phase	during heel stick procedure	Mothers	0.5h	infants were nested in prone position 30 minutes before the heel stick and remained in the same position until the end of the recovery phase	Premature infant pain profile (PIPP) scores, HR, SpO2, regional cerebral tissue oxygenation saturation (rcSO2), Cerebral fractional tissue oxygen extraction
Yildirim 2023	RCT	90	healthy infants	KC for an average of 45 minutes per day	NA	fathers	0.75h	no routine KC with father practice in postpartum care	bonding

Author/year	Study type	Sample	Participants	Intervention	Initiation	KMC giver	KMC Duration	Control	Outcomes
Yildizdas 2023	RCT	74	Newborns with gestational age ≥34 weeks	KMC 5 min and then the babies were placed in the incubator	during venous puncture	Mothers	0.08h	In massage group, in dextrose group, 3 ml of 10% dextrose was given orally while the babies were in incubator	NIPS score
Zhang 2023	RCT	659	≥37 gestational weeks with a singleton pregnancy	skin-to-skin contact，Intervention group 1 (G1), 2 (G2), and 3 (G3) received 30, 60, and 90 minutes of skin-to-skin contact immediately after the cesarean delivery	immediately	Mothers	0.5-1.5h	routine care	rates of early initiation of breastfeeding within 1 hour after birth, exclusive breastfeeding, intraoperative blood loss, NICU or neonatal ward admission before discharge, blood hemoglobin (Hb)
Zheng 2022	RCT	280	newborns showed Apgar scores of 8 ~ 10	Skin-to-skin contact，the participant and the newborn should be given continuous SSC within three days of hospitalization, with twice a day	after bath	family members	2h/d 3d	routine care	depression, the lactation, breastfeeding conditions, uterine involution, postpartum duration of lochia, pain
Zhu 2022	RCT	60	After duodenal obstruction in Neonates(>37 w and >2,500g	Kangaroo mother care was conducted in the intervention group based on ERAS conventional care，take 20 min the first time，with 2 h once a day	after surgery	mothers or fathers	2h	Implementation of the conventional postoperative ERAS care	The length of hospital stay, oral feeding outcomes

Author/year	Study type	Sample	Participants	Intervention	Initiation	KMC giver	KMC Duration	Control	Outcomes
Zou 2022	RCT	241	gestational age is 37 0/7–41 6/7 weeks of delivery	After the newborn was delivered, Skin-to-skin contact was performed immediately afterwards	immediately	Mothers	2h	The perineal wound was sutured after the placenta was delivered. After the wound was sutured, skin contact was carried out for more than 120 min	the pain of parturient during suturing, the healing of the perineal wound, the maternal cooperation rate
Ding 2018	RCT	80	full-term newborns	KC group. 1h/day	NA	Mothers	1h/d	routine obstetric nursing	oral feeding outcomes, neonatal behaviors, body temperature, weight
Wang 2018	RCT	872	full-term newborns	KMC，1h，2 times/day	early SSC	Mothers	2h/d	routine care	evaluation of first breastfeeding, oral feeding outcomes, pain, neonatal behavioral neurological, sleep quality and sleep duration, uterine involution
Gan 2018	RCT	136	preterm infants less than 2000 grams	KMC，30mins ，2 times/day	stable	Mothers	1h/d	routine care	discharge weight, discharge length, discharge head circumference, mean weight gain, incidence of extrauterine developmental delay, neonatal behavioral neurological, apnea, bronchopulmonary dysplasia, patent ductus arteriosus, IVH, feeding intolerance, gastrointestinal hemorrhage, Necrotizing enterocolitis, nosocomial infection, Fasting time, parenteral nutrition time
Zhai 2018	RCT	110	premature infants	KMC，1~2h ，2 times/day	stable	mothers or fathers	>2h	routine care	RR, HR, body temperature, daily milk intake, weight

Author/year	Study type	Sample	Participants	Intervention	Initiation	KMC giver	KMC Duration	Control	Outcomes
Zhai 2018	RCT	296	full-term infant	routine care, skin-to-skin contact,90min	immediately	Mothers	1.5h	routine care, skin-to-skin contact,30min	body temperature, cry frequency, time of foraging reflex, duration of first breastfeeding, rates of exclusive breastfeeding
Xie 2018	RCT	80	full-term infant	routine care 5~6min, skin-to-skin contact,1h	immediately	Mothers	1h	routine care 5~6min, skin-to-skin contact, 27~35min	cry time, newborn behavior, breastfeeding
李润杰, 朱丽, 张红艳, 马红 2021	Quasi-Experimental Study	84	full-term infant	KMC，2times/(after bath and 4p.m.), 60mins,3days,Carry on after discharge	after bath	Mothers	2h/d 3d	Routine care	initial milk secretion time, degeneration degree of uterus, depression
Liu 2022	RCT	64	31-35weeks 1000-2200g	KMC,15:00pm, once/day,2h,7days	stable, P7	Mothers	2h, 7d	Routine care	parenting stress, parenting sense of competence
Gai 2021	RCT	132	<37 weeks <2000g	KFC,2h	stable	fathers and mothers	2h	KMC,2h	HR
Huang 2016	RCT	71	37-42weeks ≥2500g	SSC,1h	immadiately after birth (within 10min)	Mothers	1h	SSC,20-30min	time when rooting reflex appeared, success rate of first breastfeeding, duration of first sucking, initial lactation time, rate of exclusive breastfeeding at discharge
Lu 2022	RCT	160	28-32weeks 1000-1499g	KMC,2times/day,60min(Initial time:30 mins)	after birth day 5	Mothers	2h	Routine care	hospitalization expenses

Author/year	Study type	Sample	Participants	Intervention	Initiation	KMC giver	KMC Duration	Control	Outcomes
Li 2021	RCT	59	31-35weeks 1000-2200g	KC, once/day,15:00pm,2h, day1,3,5,7	stable	Mothers	2h	Routine care	pH, PaCO2, PaO2, SaO2, RR, HR, SpO2
Huang 2021	RCT	53	29-32weeks 1200-1799g	KMC,13:00-16:00, once/day,1h,14days	stable	Mothers	1h, 14d	Routine care	gastrointestinal hormone, feeding intolerance, daily milk adding speed, time to return to birth weight
Hu 2016	RCT	90	≥37weeks ≥2500g	KMC,15mins	during vaccinations	Mothers	0.25h	wrapped in swaddling clothes	pain, HR, SaO2
Sisay Gere 2021	Crossover	52	<37weeks <2500g	CB-SSC+CC-SSC	NA	Mothers	continuous, 3d	CC-SSC+CB-SSC 3days, position alternatively every 2h,1h washout	mean skin T, skin Temperature change per arm
Darwin Cortés 2022	RCT	441	≤1200g, 1201-2000g	KMC	stable	Mothers	continuous	Traditional Care	preschool years, school absenteeism, high school graduation, math test score, language test score
Sisay Gere 2021	RCT	46	32-37weeks ≥1000g, <2500g	CB SSC 2h/day,3days	stable	Mothers	2h, 3d	CC SSC 2h/day,3days	SpO2, HR
Laleh Goudarzvand 2017	RCT	70	37-42weeks 2500-4000g	KMC, 4-5 PM	NA	Mothers	1h	conventional phototherapy	cutaneous bilirubin

Author/year	Study type	Sample	Participants	Intervention	Initiation	KMC giver	KMC Duration	Control	Outcomes
Razieh Fallah 2016	RCT	120	37-42 weeks 2500-4000 g	Intervention1:Breast feeding,2 mins before, during and 1min after vaccination Intervention2:KMC,10 mins before, during and 1min after vaccination	during vaccinations	Mothers	0.3h	swaddled,10 mins before, during and 1min after vaccination	pain, duration of crying
A. Gholami 2021	RCT	90	28-36weeks	Intervention1:massage,5mins,3times/day(1h after morning feeding, half hour after mid-day,45min after 2nd massage); Intervention2:KC,started from 20 to 30 mins once/day and gradually increased to 1 to 3h 3times/day	stable	Mothers	3-9h	usual care	covert anxiety, pain
K. H. M. Cooijmans 2021	RCT	116	≥37weeks ≥2500g	SSC,≥1 daily uninterrupted hour, the first 5 post_x005f natal weeks, starting immediately after birth	immediately	Mothers	1h/d	care-as-usual	exclusive breastfeeding duration, continued breastfeeding duration
Wu Li 2022	quasi-experimental design	347	34-36 weeks	KMC, encourage continuous and long-term skin contact; If the mother interrupts KMC due to physical factors or no other family members participate，perform KMC every morning, noon, and evening, no less than 1h	as soon as possible after childbirth.	Mothers	continuous	placed in a stroller and followed by medical care	body temperature, RR, pain, sleep, number of vomiting, the number of daily crying and the duration of each crying, Breastfeeding situation, Breastfeeding situation, Success rate of first breastfeeding, Exclusive breastfeeding rates, Time of first successful breastfeeding(h), Achieve exclusive breastfeeding time(h)

Author/year	Study type	Sample	Participants	Intervention	Initiation	KMC giver	KMC Duration	Control	Outcomes
Emel Avcin 2021	quasi-experimental design	140	≥ 37 weeks, > 2500 g,	breastfeeding:5 mins before blood collection;<br>KC: during blood collection<br>facilitating tucking position:1 min before blood collection	during procedure (blood collection)	Mothers	0.2h	Routine care	crying time, body<br>temperature, oxygen saturation, heart peak heart rate, RR
Sibel Kucukoglu 2021	quasi-experimental	60	>28 to <37 weeks	KC,30min, at least once/day, afternoon, every day during the hospital stay and until the end of the first month after discharge	stable	Mothers	0.5h	Routine care	body weight, length, the daily time and duration of KC
Fatemeh Zahra Karimi 2016	RCT	65	term pregnancy with healthy infant	KMC, in the first 2h post birth and uninterrupted minimum 30 mins at a time during the neonatal period, day and night	Immediately	Mothers	>0.5h	routine hospital care	Mother-infant Attachment, Anxiety
Mamta Jajoo 2022	RCT	50	1001-1999 g, 30-40 weeks	KMC,1h every 8h	stable	Mothers	3h/d	phototherapy in a conventional manner,24h/day	Duration of phototherapy
K. Swarnkar 2016	quasi-Experimental Study	60	<2500g	KMC,minimum total period of 8h/day	stable	Mothers	>8h	incubators or cots	weight gain, length, head circumference, Hospital stay, time to start breast feed, Exclusive Breast feeding at 42 weeks, morbidities
Parida Hanum 2022	quasi-experimental	40	<2500g	intermittent KMC,2 h,once/day	after birth	Mothers	2h	standard NICU unit procedures	rooting-sucking reflex, duration of KMC

Author/year	Study type	Sample	Participants	Intervention	Initiation	KMC giver	KMC Duration	Control	Outcomes
N. Rasouli Larma'i 2016	RCT	106	Healthy term newborns	KMC,30-45min, at least 6times/24h，morning, afternoon, night	NA	Mothers	3-4.5h	Conventional phototherapy	Bilirubin level, duration of phototherapy or length of hospital stay
Rafael Moura Miranda 2022	RCT	26	28-34weeks	KC position,8-12h/day	NA	Mothers	8-12h/day	resting in the crib	blood flow, tissue oxygen saturation, tissue temperature
Deepak Sharma 2016	RCT	141	<32weeks <1100g	KMC, at least 6h/day	stable	Mothers	>6h/d	intermediate intensive care	weight, head circumference, length, breastfeeding, hospital stay, mortality, aspiration pneumonia, malnutrition, anemia, readmission to NICU, cystic periventricular leukomalacia, retinopathy of prematurity
Gao 2015	RCT	75	<37weeks	in 30min before, and throughout the recovery phase of the heel stick，left for 30min	during heel stick procedure	Mothers	0.5h	incubator	crying, grimacing, HR
Kamile Çiftci 2022	RCT	148	32-37weeks	Intervention1: KC,5min before procedure util 1min after procedure Intervention2: Fetal position Intervention3:Swaddling	during peripheral vascular access procedure	Mothers	0.1h	routine peripheral vascular access	NIPS
RETNO MAWARTI 2021	quasi-experimental	60	all newborn babies	KMC+KFC	NA	mothers and fathers	NA	KMC only	heavy hypothermia, light hypothermia, temperature
Chen 2017	RCT	83	≥37weeks	SSC, at least 15min,daily,during the first 3 days postpartum	first three days postpartum	fathers	0.25h	standard care	Father-Child Attachment

Author/year	Study type	Sample	Participants	Intervention	Initiation	KMC giver	KMC Duration	Control	Outcomes
Young Sun Seo 2016	quasi-experimental	56	≥37weeks ≥2500g	KMC,10 min before sampling and continued 3 min after sampling	during procedure (blood collection)	Mothers	0.2h	fed 1 h before the initiation of the heel stick	HR, SpO2, pain, the duration of crying
Farideh Bastani 2017	RCT	70	32-37weeks <1500g	KC,70min	NA	Mothers	1.1h	in-arms_x0002_holding	The mean of deep sleep, The mean for quiet awake
Shahin Dezhdar 2016	RCT	90	<37 weeks <2500g	Intervention1: Swaddling Intervention2:KMC,10 min, sampling, maintained for a further 2min	during venous puncture	Mothers	0.2h	Routine care	HR, SaO2, pain
Amit Sharma 2016	RCT	199	Term	SSC	Immediate	Mothers	0.57h	radiant warmer for a period of 45 min	breastfeeding, length, occipit -frontal circumference, weight, times of urine, times of stool
Huang 2022	Quasi-Experimental	104	37-42weeks 2500-4000g	Immediate SSC,60min	Immediate	Mothers	1h	early SSC,20 min	newborn sucking ability, breastfeeding self-efficacy, success rate of breastfeeding, exclusive breastfeeding rate
Agnes Linnér 2022	RCT	91	28-34weeks	immediate SSC, a parent after birth and continued throughout the first 6 h	Immediate	mothers or fathers	6h	standard incubator care	SCRIP scores, adverse event, Initiation and duration of SSC
Helen Brotherton 2021	RCT	277	<2000g age1-24h	KMC, continuous SSC，>18 h/day in prolonged sessions	early initiated <24 h after admission	caregivers	continuous	Intermittent KMC at least 60min SSC several times/day	all-cause mortality, time to death, cardiorespiratory stability, hypothermia, exclusive breast feeding, clinically suspected infection, blood culture confirmed infection, duration of admission, weight gain (g/day)

Author/year	Study type	Sample	Participants	Intervention	Initiation	KMC giver	KMC Duration	Control	Outcomes
Aditi Bose 2021	Quasi-experimenta l	80	Cesarean	early SSC at least 45mins	early SSC	Mothers	0.75h	no intervention	breast feedings score, HR, temperature, maternal satisfaction
Mahnaz Jabraeili 2017	RCT	105	cesarean with spinal anesthesia;37-42 weeks	SSC,3 days, at 7 am, 1 pm, and 7 pm for 30 min	immediately	Mothers	0.5h	Routine care	the satisfaction of the mothers
ARCHANA SOMASHEK HAR NIMBALKAR 2016	RCT	100	Term, >1800g	early SSC, beginning of 30min-1h after birth, average 17h on day1 of life	The KMC performed with support of the researchers in the recovery room for 30 min.	Mothers	17h	conventional care	Infant and young child feeding practices
Marzieh Mohammadi 2021	Quasi-Experimenta l Design	208	Preterm neonates, <2500g	Continuous KMC	NA	Mothers	continuous	did not receive C-KMC	weight, head circumference, length, breastfeeding, Practice of C-KMC, Duration of hospitalization
S.N.Lumbanra ja 2016	RCT	40	1000-2500g	KMC,4-6h/day	stable	Mothers	4-6h/day	conventional care	Distribution of Z score for babies' weight, height, head circumference, weight gain
Choudhary 2016	cross-over design	140	28-30 weeks	KMC	During heel-lance	mothers	KMC, 24h	conventional care	HR, SaO2, PIPP score, duration of cry
Sangita Kumbhojkar 2016	RCT	120	<2000g	at least 1-2h/time	stable	Mothers	1-2h	conventional care	weight gain, head circumference, length, hospital duration, hypothermia, sepsis, apnea, exclusive breastfeeding rate

Author/year	Study type	Sample	Participants	Intervention	Initiation	KMC giver	KMC Duration	Control	Outcomes
Mahboubeh Jamehdar 2022	RCT	70	32-35weeks	KMC(mother and surrogate)60min,3times/day(mother 1,surrogate2),4days	NA	Mothers and surrogate	3h	KMC(mother),60min, 3times/day,4days	feeding behavior, HR, RR, temperature, arterial oxygen saturation
Laura Collados-Gómez 2022	RCT	70	<28weeks	KC, lateral position, at least 60min/day	hemodynamically stable	mothers OR fathers	1h/d	KC, prone position, at least 60min/day	axillary temperature, pain, Intraventricular hemorrhage
S. Gavhane 2016	RCT	91	<1500g	KMC, at least 8h/day	stable	Mothers	>8h/d	conventional care	Malnutrition, wasting, stunting, height, small head, feeding information, exclusive breastfeeding rate
Kim Chi Luong 2016	RCT	100	1500-2490g	SSC	stable	Mothers	NA	Routine care	the stability of cardio-respiratory system, hypothermia
S. Jamil 2021	RCT	226	1500-2499g	Intermittent KMC,4hourly intervals for 7 days	stable	Mothers	>8h	conventional care	body weight, mean duration of hospital stay
Anju Kapoor 2021	RCT	149	34-36weeks	Intervention1:KMC,at least 30min before prick<br>Intervention2:D50 (0.5 ml/kg solution administered before prick	during procedure	Mothers	0.5h	supine nesting position	PIPP score, HR, SpO2
V. V.Shukla 2021	RCT	128	28-36weeks	KMC,15min before 1st stick+KFC 2nd stick	during procedure	mothers or fathers	0.3h	KFC,15min before 1st stick+KMC 2nd stick	PIPP score

Author/year	Study type	Sample	Participants	Intervention	Initiation	KMC giver	KMC Duration	Control	Outcomes
Kristin P. Tully 2016	RCT	231	>1000g <1750g	KC,at least 15min	NA	Mothers	0.3h	Control1:auditory-tactile-visual-vestibular, 3times/week til 2months adjusted age; Control2:preterm infant care information	depression symptoms, maternal situational anxiety, post-traumatic stress symptoms, worry about child health, parenting stress
Nathalie Charpak 2017	RCT	264	<2000g	KMC	NA	NA	NA	traditional care	cumulative mortality at 20 Years, overall IQ at 20 Years, asthma, epilepsy, frequent accidents (frequent falls), endocrine system alteration, hypothyroidism, precocious puberty, short stature, learning disability, severity of neuromotor abnormality at 20 y(With disability/Without disability), mental illness, years of preschool, school absenteeism, years of school, mathematics score
2014	RCT	140	32~36w	OTHER: Continuous KMC to 1 hour after birth, OTHER: Standard KMC to 1 hour after birth, OTHER: Continuous KMC to discharge, OTHER: Standard KMC to discharge	immediately	Mothers	Continuous KMC	NA	axillary temperature<36℃, blood pressure, blood glucose, seizure, seizure activity, respiratory distress syndrome (RDS), sepsis, NICU Admission, death, cardiorespiratory failure, any axillary temperature < 36.0 ℃, duration of KMC

Author/year	Study type	Sample	Participants	Intervention	Initiation	KMC giver	KMC Duration	Control	Outcomes
2014	RCT	375	≥37w	OTHER: Continuous KMC to 1 hour after birth, OTHER: Standard KMC to 1 hour after birth, OTHER: Continuous KMC to discharge, OTHER: Standard KMC to discharge	immediately	Mothers	Continuous KMC	NA	number of infants with axillary temperature <36.0 ℃, number infants admitted to the NICU or higher level care, duration of KMC
2015	RCT	90	≥28w	Kangaroo Care	NA	Mothers	2-3h	no intervention	EEG-based sleep, HR, RR
2015	RCT	550	CHILD	Behavioral: KMC, Other: essential newborn care	as soon as possible after birth	Mothers	NA	NA	maternal depression, maternal sense of competence, mother infant bonding, mother infant interaction, newborn behaviour, infant temperature, cognitive function, home environment
2016	RCT	8402	≥1500 to ≤2250 g	KMC	as soon as possible after birth	Mothers	NA	No Intervention	mortality, proportion exclusively breastfed, weight gain, length gain, incidence of infection and hospitalization, early recognition of illness, early appropriate care seeking practices, cognitive function, head circumference
2016	RCT	2221	≥700g and ≤2000g	KMC at least 18 hours per day	initiated as soon as possible following randomisation	Parent/caregiver	>18h	Standard care	mortality, prevalence of hypothermia, time from intervention/control procedures starting to clinical stabilization, time from intervention/control procedures starting to death, mean duration of hospital stay in days, proportion of neonates exclusively breastmilk feeding at discharge, frequency of readmission, daily weight gain, Infant-caregiver attachment, women's well-being at 28 days

Author/year	Study type	Sample	Participants	Intervention	Initiation	KMC giver	KMC Duration	Control	Outcomes
2016	RCT	50	27-34 w	KMC	NA	NA	NA	No Intervention	electromyographic activity, tissue oxygen saturation, relative oxygenated haemoglobin concentration - oxyHb, relative deoxygenated haemoglobin concentration - deoxyHb, Relative total haemoglobin concentration -totalHb, temperature, blood flow
2016	RCT	35	≥1800g	KMC	NA	Mothers	NA	No Intervention	breastfeeding outcome, Body temperature, HR, RR
2016	RCT	110	37 to 42 w	Snuby® skin-to-skin facilitating garment	NA	Mothers	NA	No Intervention	abnormal neonatal temperature, breastfeeding status, mother infant bonding, neonatal weight velocity, maternal participant's perspective, midwifery participant's perspective
2017	RCT	50	≤ 37 w	Applying music therapy with kangaroo care to mothers and fathers	NA	mothers and fathers	NA	Applying only Kangaroo care to mothers and fathers	parasympathetic tone
2017	RCT	0	≥32 0/7 - 36 6/7 w	Other: KMC & WHO protocol (0-1 hour) Other: KMC, WHO protocol & bag (0-1 hour), Other: KMC & WHO protocol (1-24 hours) Other: KMC, WHO protocol & bag (1-24 hours)	immediately	Mothers	continuous	NA	moderate or severe hypothermia, hyperthermia, skin rash, NICU admission, NRDS, sepsis, hypotension, hypoglycemia or seizure, morbidity, death, mortality, maternal body temperature, temperature, duration of KMC and plastic bag to infant's body temperature
2017	RCT	240	> 37 w	KMC (a video education)	NA	NA	NA	no video	intention to practice skin-to-skin contact after birth

Author/year	Study type	Sample	Participants	Intervention	Initiation	KMC giver	KMC Duration	Control	Outcomes
2018	RCT	91	28+0 - 32+6w	SSC: continuous SSC with one parent/caregiver the first 6 hours after birth and as much as possible the first 72 hours after birth.	the first 6 hours after birth	parent/caregiver	continuous	conventional care	cardiorespiratory stability, need for respiratory support, need for surfactant, need of continuous positive airway pressure, ceed for oxygen, HR, temperature, sepsis episodes, status of breast-feeding, time to full enteral nutrition, time to recovered birth weight
2018	RCT	2000	≥1200-<2500 g	Community based Kangaroo Mother Care	within 48 hours after birth	mothers or caregiver	continuous	essential newborn and routine standard care	neonatal mortality, epigenetic profiling in buccal cells, telomere profiling in buccal cells，DNA-analysis from stool samples, DNA-analysis from stool samples, anxiety, saliva cortisol both mother and child, neuro behaviour of child, depression
2018	RCT	279	<2000g	Early KMC: Continuous kangaroo mother care started within 24h of hospital admission, aiming for minimum 18h/day and until hospital discharge	within 24h of hospital admission	mothers or caregiver	continuous>18 h/d	Standard care	all-cause mortality, time to death, cardiorespiratory stability, number and proportion of Participants With Hypothermia, Weight Gain, Exclusive Breastfeeding, Suspected Infection, Pneumoniae, Duration of Hospital Admission
2018	RCT	66	≤33 w	Kinesthetic Stimulation in Kangaroo Position	NA	parent	15d	Kinesthetic Stimulation In incubator	weight gain
2018	RCT	50	27~37w	Kangaroo position	NA	NA	NA	no intervention	electromyographic activity, oxygen artery saturation, HR, oxygen saturation, oxygenated hemoglobin concentration, eoxygenated hemoglobin concentration, temperature, oxygen flow,

Author/year	Study type	Sample	Participants	Intervention	Initiation	KMC giver	KMC Duration	Control	Outcomes
2018	RCT	120	31~35w	KMC 60 min or 120 min	stable	Mothers	1-2h,7d	conventional care	neurodevelopment, cortisol
2018	RCT	126	32～36w	skin-to-skin with their mother at least fifteen minutes prior to heel lance	during heel stick procedure	Mothers	0.2h	24% Oral sucrose	pain, HR, Maternal acceptability
2019	RCT	140	30~37w	Singing Kangaroo: singing during the skin-to -skin sessions	NA	parent	NA	Silent Kangaroo	auditory cortical responses, parental anxiety, neurocognitive development, language development
2019	RCT	70	<28w	Posture lateral kangaroo care	NA	parents	NA	Posture prone kangaroo care	Change from skin temperature
2020	Crossover	46	32~40w	Kangaroo Care: half an hour	NA	Mothers	0.5h	NA	test weighing, HR, oxygen saturation
2020	RCT	380	<2500g	KMC with bracelet	early KMC	Mothers	continuous	KMC	hypothermia, weight, proportion of babies who received KMC, all cause mortality, exclusive breastfeeding
2020	RCT	214	36~38w	KMC	immediately	Mothers	0.15h	no intervention	breastfeeding conditions, weight, height, head circumference, baby's development, maternal attachment
2020	RCT	112	38-40w	KMC	immediately	Mothers	3h	no intervention	breastfeeding outcomes
2021	RCT	32	neonates	Mindful Kangaroo Care	stable	Mothers	NA	Standard care	change in stress symptoms, change in anxiety symptoms, change in depression symptoms, acceptability
2021	RCT	106	24-37w	KMC at least 1 hour	NA	Mothers	>1h	Standard care	Oxygen Saturation, HR, SpO2, pain, RR,
2022	RCT	120	28～37w	KMC	stable	Mothers	6h/d, intermittent KMC	standard care	temperature, HR, oxygen saturation

Author/year	Study type	Sample	Participants	Intervention	Initiation	KMC giver	KMC Duration	Control	Outcomes
2021	RCT	143	mother	Scarf specifically designed to facilitate mother-neonate skin-to-skin contact	Two hours after delivery	Mothers	NA	Usual clinical practice	mother-neonate skin-to-skin contact time, time in arms or in physical contact of the neonate with the mother / father, time of crying, newborn weight loss, parents satisfaction with hospital care
2021	Crossover	36	32~42w	SSC	during heel stick procedure	Mothers	0.5h	30% oral glucose	pain, change in the activation in the somatosensory cortical areas following the noxious stimulation (baseline), HR, change in oxygen saturation, RR
2021	RCT	102	37~42w	KC	NA	Mothers	NA	no intervention	Physiological weight loss evaluation
2021	RCT	90	Preterm	KMC, Hammock Positioning	stable	Mothers	NA	ICU Routine Care	HR, RR, oxygen saturation, neonates' temperature, neonates' weight, sleep
2022	RCT	280	>37w	Early SSC: within the first 30 minutes of birth	immediately Early SSC: within the first 30 minutes of birth	Mothers	NA	Cesarean delivery and SSC one hour after birth	Breastfeeding
2022	RCT	90	fathers, healthy baby	kangaroo father care, the first KC was applied within the first 4-6 hours after birth, mean 15-20 minutes.	within the first 4-6 hours after birth	fathers	0.3h	introductory information form	bonding
2022	RCT	50	27~36w	KC	NA	Mothers	1-1.3h	standard care	the transition period of preterm infants fed by gavage to full oral feeding
2022	RCT	92	28~37w	KC	NA	Mothers	NA	No Intervention	Kangaroo care relieves stress
2022	RCT	33	<34w	KMC with Massage therapy	NA	NA	NA	No Intervention	weight, head circumference, length of hospital stay in days

Author/year	Study type	Sample	Participants	Intervention	Initiation	KMC giver	KMC Duration	Control	Outcomes
2022	RCT	160	<2500g	KC	NA	Mothers	2h/d, 6m	No Intervention	breastfeeding, mother infant attachment, mother sleep quality, postpartum depression
2023	RCT	60	37~42w	SSC	immediately	Mothers	0.2h	No Intervention	Pain
2023	RCT	40	26~37w	Kangaroo care was applied for 65 minutes	stable	Mothers	1h	No Intervention	RR, HR, oxygen saturation, body temperature, pain, Parent Satisfaction points, nurse parent support score
2023	RCT	118	24~34w	SSC: transferred using a direct skin-to-skin contact with their father from the delivery room to the intensive neonatal care.	during the transfer between the delivery room and the NICU	fathers	NA	transferred in an incubator set to 36°C	Change in the infant skin temperature, Change in the infant's heart rate, Occurrence of bradycardia episodes in infant during the transfer, Change in the infant's oxygen saturation, Duration of the transfer procedure, PH, Carbon dioxide partial pressure (pCO2), Blood glucose, Occurrence of hypothermia, Time to the first SSC in the NICU, Quality of parental bond, Parental stress, Weight, Height, Head circumference, mortality, morbidity, Hospital stay, breastfeeding outcomes, parents' experience of their infant hospitalization
2023	RCT	20	26~36w	KC	NA	Mothers	NA	no intervention	Heart Rate Variability, Cardiorespiratory stability
2023	RCT	11440	<28w	KC	NA	NA	NA	no intervention	Neonatal severe infection/sepsis, Resistant bacterial colonization over time, Incidence of neonatal sever infections, Incidence of neonatal morbidity, Incidence of SSIs, Prevalence of breastfeeding, Incremental cost-effectiveness ratio (ICER), Budget impact of the intervention arm

Author/year	Study type	Sample	Participants	Intervention	Initiation	KMC giver	KMC Duration	Control	Outcomes
2023	Crossover	25	neonates	KC: 1h	NA	parents	1h	no KC	Breast milk calories (kcal/dL), Breast milk fat (g/dL), Breast milk protein (g/dL), Breast milk carbohydrate, Breast milk volume, Implementation of scheduled Kangaroo Care Visits in the UC Davis NICU - KC time, Implementation of scheduled K C Visits in the NICU - KC incomplete, Implementation of scheduled Kangaroo - Parental attitudes, Implementation of scheduled KC- Provider attitudes, Duration of breast milk provision, Infant growth (Birth/discharge weight and discharge weight in grams, Birth length and discharge length in cm), Infant growth (Birth/discharge head circumference, Infant growth (Birth weight z-score and discharge weight z-score), Infant growth (Birth/discharge length z-score, Infant growth (Birth/discharge head circumference z-score
2023	RCT	90	32~37w	SSC; begin 5 minutes before blood collection, continue throughout the procedure, and skin to skin will continue until 5 minutes after the procedure. Gentle Human Touch	during procedure (blood collection)	Mothers	0.1h	no intervention	Preterm Pain, Preterm Crying, HR, Oxygen Saturation

Author/year	Study type	Sample	Participants	Intervention	Initiation	KMC giver	KMC Duration	Control	Outcomes
2023	Quasi-Experimental Design	2500	1200~200g	KMC	NA	Mothers	NA	NA	number of infants having birth weight of 2000 grams received KMC, increase in exposure to KMC priming interactions in the antenatal period, proportion of the health facilities providing KMC services, number of infants received early initiation breastfeeding, exclusive breast feeding
2023	RCT	80	27~34w	Sustained Diagonal Flexion positioning	NA	Mothers	NA	Sustained Diagonal Flexion positioning	daily duration of skin-to-skin during postnatal hospitalization, continuation of skin-to-skin after discharge, Breastfeeding at discharge, Cardiovascular stability of the infant, Physiological Stability of the Cardiorespiratory System in Premature Infants (SCRIP) score, Physiological parameters, Physiological parameters, Physiological parameters, pain score, Position of joints and muscle tone, Parental psychological wellbeing - French version of the Parental Stressor Scale (PSS), Parental psychological wellbeing
2023	Crossover	38	26~37w	KC: for 65 minutes, and after a 24-72 hour washout period, the father applied kangaroo care for 65 minutes.	NA	mothers and fathers	1h	NA	RR, HR, oxygen saturation, body temperature, comfort behavior scale scores of the premature infant, parent satisfaction points, NICU parental stress scale points

Author/year	Study type	Sample	Participants	Intervention	Initiation	KMC giver	KMC Duration	Control	Outcomes
2023	RCT	80	28~42w	Gentle Human Touch; KC	during vaccination	Mothers	0.1h	NA	pain level, newborn crying duration, oxygen saturation, HR, RR
2024	Quasi-Experimental Design	50	neonates	KC, Music therapy	NA	caregivers	NA	NA	weight, oxygen saturation, HR, parenting stress index, general movements, neurological assessment, levels of noise in NICU
2024	RCT	50	36～38w	KMC	NA	parents	NA	no intervention	fathers' attachment level
2024	RCT	300	neonates	KMC with the thermal jacket	NA	caregivers	NA	conventional KMC	body temperature
2024	RCT	90	≥37w	KC with white noise; KC	during heel stick procedure	Mothers	>1h	no intervention	total crying time, values of pulse, oxygen saturation, cortisol
2024	Crossover	100	28~37w	kangaroo care by both their mother and father.45 minutes, 2 days a week for 5 weeks.	NA	mothers or fathers	0.75h	KC by mother	mother-to-infant bonding, perceived mother parenting self-efficacy scale, postnatal paternal-infant attachment, perceived father parenting self-efficacy, HR, oxygen saturation, body temperature, RR
2024	RCT	40	24~36W	KMC: on third day of life.	on third day of life.	Mothers	1h	no intervention	biomarker of oxidative stress, mother- infant bonding
2024	RCT	136	Term single birth	KC, twice a day and for 60 minutes every day for 6 months in a row; Half swaddle+ KC	NA	Mothers	2h/d, 6m	no intervention	mother sleep quality, postpartum depression
2024	RCT	120	38~42w	KC; swaddling group	during heel stick procedure	Mothers	0.2h	no intervention	neonatal infant pain, crying times
2024	RCT	76	24-37w	KMC	NA	Mothers	NA	no intervention	perfusion index, HR, oxygen saturation, preparation

Author/year	Study type	Sample	Participants	Intervention	Initiation	KMC giver	KMC Duration	Control	Outcomes
2024	RCT	60	> 37 w	KC, provide skin-to-skin contact for at least 1 hour per day for seven days, starting immediately after feeding	after surgery	Mothers	1h/d	standard care	change in oxygen saturation, change in heart rate, change in respiratory rate, change in blood pressure, change in level of pain
2024	RCT	36	32~36w	Hammock position group; KC Group	NA	Mothers	NA	no intervention	determining the descriptive characteristics of babies, comfort level, decrease pain and stress level, physiological stability
2014	RCT	50	<2000g	kangaroo tube for 4 hours minimum per day for 7 days.	NA	Mothers	4h/day, 7d	NA	maternal serum prolactin, the adequacy of breast milk emptying, breastfeeding outcomes, infant's weight gain
2018	RCT	4200	1000~1800g	continuous SSC initiated as soon as possible after birth with a mother or a surrogate, at least 20 h/d, continued during entire hospital stay	immediately	mother or a surrogate	continuous, >20h/d	NA	mortality, exclusive breastfeeding, time to being fully breastfed, suspected sepsis, hypothermia, hypoglycemia, time to clinical stabilization, maternal satisfaction, maternal depression
2019	Quasi-Experimental Design	76	31~33w	KMC	stable	Mothers	NA	NA	amplitude integrated EEG, regional cerebral oxygen saturation, neonatal behavioral neurological assessment, pain, HR, SPO2
2020	RCT	120	37～41w	early skin-to-skin contact	early skin-to-skin	Mothers	NA	non-early	pain, faces pain scale-revised, maternal cooperation degree, healing of maternal perineal tears wounds
2020	RCT	54	29~32w	KMC	NA	NA	NA	NA	gastrin, motilin, intestinal flora
2022	Quasi-Experimental Design	60	>37w	KMC	after surgery	NA	NA	NA	effects of kangaroo nursing on feeding after duodenal obstruction in neonates

Author/year	Study type	Sample	Participants	Intervention	Initiation	KMC giver	KMC Duration	Control	Outcomes
2024	Quasi-Experimental Design	120	38～41w	KMC	NA	Mothers	NA	Postpartum routine care	depression, fatigue, satisfaction, the volume of blood loss in the first 24 hours after birth, the time of the first lactation
2017	RCT	50	32~34w	early father-infant SSC	early father-infant skin to skin care	fathers	NA	routine nursing	behaviour state of preterm infant
2014	RCT	200	<37w	KMC in first 6 hours of life	in first 6 hours of life	NA	NA	conventional care	requirement of supplemental oxygen, requirement of ventilation
2016	RCT	10500	1500~2250g	KMC	NA	Mothers	NA	Routine visits	mortality, infection, illness, weight, length and head circumference, proportion exclusively breastfed, early appropriate care seeking practices, motor and mental development
2016	RCT	550	1500~2250g	early SSC	early SSC	Mothers	NA	Routine Care	maternal depression, maternal sense of competence, mother infant bonding , mother infant interaction, newborn behaviour, infant temperament, cognitive function, home environment, neonatal mortality, early initiation of breastfeeding, proportion exclusively breastfed, weight and length gain, incidence of infection in neonatal, illness, early and appropriate care seeking practices
2016	RCT	200	26~36w	Music therapy, KMC	stable	Mothers	NA	music therapy	pain, HR, oxygen saturation
2017	RCT	100	26~36w	KMC, at least 10 min in advance before procedure	during procedure	caregivers	0.2h	Sucrose reduces pain	Pain

Author/year	Study type	Sample	Participants	Intervention	Initiation	KMC giver	KMC Duration	Control	Outcomes
2017	RCT	60	28~32w	Music therapy, KMC	NA	Mothers	NA	NA	breast milk volume expressed, maternal stress, mean change in volume of milk expressed from
2021	RCT	4600	1500~2250g	KMC, early skin to skin care as soon as possible	early skin to skin care as soon as possible	Mothers	NA	No intervention	LAZ score, postpartum depressive symptoms, Relative abundance of and diversity of infant gut microbiome, breast milk antimicrobial proteins, breastfeeding performance, cortisol levels, infant breast milk intake during the neonatal period, WHZ score, gut inflammatory markers
2021	RCT	50	30~40w	KMC: will receive phototherapy with the same settings along with one hour of KMC eight hourly	NA	mothers or caregiver	3h/d	conventional care	duration of phototherapy, temperature, rate of fall in serum bilirubin, weight gain, hospital stay
2017	RCT	4502	1500~2250g	KMC	NA	NA	NA	No intervention	mortality, total expenditure/consumption and income of households, health care utilization, household out of pocket expenses for episode(s)
2017	Quasi-Experimental Design	100	<37w	Baby in KMC will be undergoing Heel prick	during heel stick procedure	NA	NA	Oral Dextrose 50%	pain
2017	Crossover	52	28～34w	KC father for 1 h	stable	fathers and mothers	1h	KC Mother for 1 h	HR, salivary cortisol levels, oxygen Saturation
2018	RCT	128	neonates	SSC given by Mother: KMC will be provided at least 10 min in advance and would continue the intervention at least 5 min post procedure.	during procedure	mothers or caregiver	0.2h	KMC by father	pain

Author/year	Study type	Sample	Participants	Intervention	Initiation	KMC giver	KMC Duration	Control	Outcomes
2018	RCT	140	28～34w	package: KMC, exclusive breast milk feeding, non-nutritive sucking	NA	Mothers	NA	routine care	exclusive breast milk feeding
2018	Crossover	100	28~36w	KMC at least 15 min in advance and would continue the intervention at least 15 min post procedure.	during procedure	caregivers	0.5h	Sucrose	pain
2018	RCT	128	1000~2000g	KMC	NA	NA	NA	routine care	KMC duration, weight, morbidity, hypothermia
2018	RCT	4200	1000~1800g	immediately KMC, continuous skin-to-skin contact/ kangaroo mother care initiated immediately after birth with mother	immediately	Mothers	continuous	KMC after stabilization	mortality, time to stabilization, hypothermia, risk of infection, time to hospital discharge, exclusive breastfeeding, maternal satisfaction, maternal depression, time to being fully breastfed, suspected sepsis, hypoglycemia
2019	Crossover	136	28～36w	four duration of KMC, 10min vs 15min vs 20min vs 30min	during procedure	NA	0.1-0.5h	NA	beneficial effects of short duration of KMC
2019	RCT	3000	1000~1800g	immediate KMC, continuous SSC	immediately	Mothers	continuous	KMC after stabilization	developmental delay
2020	RCT	100	> 37 w	Immediate SSC, at least 35 minutes	Immediate	caregivers	0.5h	routine care	pain
2020	RCT	152	neonates	SSC	during transport	NA	NA	routine care	prevalence of hypothermia
2021	RCT	96	<37w	KMC plus Instrumental music for 5 days with play time being minimum of 90 minutes per day	stable	Mothers	1.5h/d, 5d	KMC alone for 5 days	neurodevelopmental outcome, maternal stress, neonatal stress, weight gain, exclusive breast feeding rate

**Author/year**	**Study type**	**Sample**	**Participants**	**Intervention**	**Initiation**	**KMC giver**	**KMC Duration**	**Control**	**Outcomes**
2021	RCT	126	<1500g	KMC started within 7d	started within 7d	Mothers	NA	start receiving KMC after they attain full feeds and hemodynamically stable	hospital stay, the incidence of sepsis, necrotizing enterocolitis, bronchopulmonary dysplasia, intraventricular hemorrhage, apnoea, feed intolerance, extra-uterine growth retardation (EUGR), neurodevelopmental outcome, mortality rates,
2023	Quasi-Experimental Design	500	<37w	immediate KMC, initiated within 2 hours after birth, for at least 8 hours per day during the stay in the level 2 M-SNCU	immediate KMC, initiated within 2 hours after birth	Mothers	continuous >8 h/d	Conventional care	proportion of preterm or LBW infants who died, clinical sepsis, proportion of preterm or LBW infants who has hypothermia, exclusively breastfed, median age at initiation of breastmilk feeding, proportion of preterm or LBW infants who has hypoglycemia, proportion of preterm or LBW infants receiving KMC at discharge
2023	RCT	60	1000～1500g	Immediate KMC before 72 hours of life	Immediate KMC before 72 hours of life	Mothers	continuous	KMC after 72 hours of life	neonatal mortality, time taken for physiological stabilization, hypothermia, time taken to reach full breast feeding, hypoglycemia, clinically suspected sepsis, duration of hospital stay, weight gain
2023	RCT	90	<2000g	Skin to Skin Contact:	NA	Mothers	NA	Cloth to Cloth Contact	duration of SSC, Growth, temperature, time to initiate KMC
2023	RCT	90	1500~2500g	BEMPU DEVICE	NA	Mothers	NA	KMC	assess parent compliance to kangaroo mother care, correlation between compliance to kangaroo mother care and growth parameters of babies, correlation between compliance to kangaroo mother care and early breastfeeding

Author/year	Study type	Sample	Participants	Intervention	Initiation	KMC giver	KMC Duration	Control	Outcomes
2023	RCT	100	<37w	KMC	during procedure	Mothers	NA	expressed breast milk administration	pain, hemodynamic stability
2023	RCT	70	<1800g	KMC	stable	partents	NA	conventional care	hemodynamically stable, weight, head circumference weekly, length, hypothermia, hypoglycemia, morbidities, mortality
2023	RCT	3000	<37w	Immediate initiation of KMC within 2 hours of birth or admission	immediately	NA	NA	initiation of KMC only after clinical stabilization	proportion of preterm or LBW infants who died, proportion of KMC, Proportion of preterm or LBW infants on respiratory support, proportion of preterm or LBW infants receiving KMC, proportion of preterm or LBW infants who are exclusively breastfed at discharge, implementation outcomes, median age at initiation of breastmilk feeding, clinical sepsis, hypoglycemia, hypothermia, proportion of preterm or LBW infants receiving KMC
2024	RCT	516	neonates	KMC for a minimum of 8 hours per day during the first 72 hours of the newborn life.	early KMC, within first 72h	Mothers	continue,>8h/d	standard care	weight loss in initial 48 hours, weight gain, moderate to severe hypothermia, mean daily duration of SSC during facility stay, proportion of infants exclusively breastfed, maternal depression, morbidities, hospitalization rate, mortality,
2024	RCT	400	<37w	KMC+Coconut oil application	NA	NA	NA	KMC	weight gain, infection rate, readmission rate

Author/year	Study type	Sample	Participants	Intervention	Initiation	KMC giver	KMC Duration	Control	Outcomes
2024	RCT	105	neonates with jaundice	Intervention1: Yaksons touch. Intervention2: KMC,30mins, 2 times/day	during light treatment	Mothers	1h/d	Routine phototherapy	reduction of serum bilirubin levels, duration of phototherapy, hospital stay
2022	RCT	165	32-37 W	Intervention 1: KC group,30 mins,3 times/day<br>Intervention 2: The not scheduled KC group. This care will be performed at any time and according to the mother's desire.	NA	Mothers	1.5h	KC not be used	mother's anxiety, mother's depression, general Health, depressed mood
2014	Quasi-Experimental Design	50	Infants between 15 and 60 days	KC position at least 2 hours/day.	NA	Mothers	2h	NA	duration of crying and fussiness
2014	RCT	105	32-37 w	Intervention 1: KMC with telephone advice group: 2-4 hours 1~3 times /day. Intervention 2: KMC group: 2-4h,1~3 times/day.	NA	Mothers	2-12h/d	infant's severe crying, defecation, sleep and breast-feeding that will be recorded daily .no further action.	height, weight, head circumference, chest circumference, Severe crying, defecation, sleep, number of breast-feeding,
2014	RCT	100	healthy baby	researcher teaches attentive samples. KC brochures and C.D s are given to mothers.	NA	Mothers	NA	Essential training for usual care	promotion of maternal general health

Author/year	Study type	Sample	Participants	Intervention	Initiation	KMC giver	KMC Duration	Control	Outcomes
2015	RCT	240	28-36 w	KFC, KMC	NA	mothers and fathers	NA	Routin care or incubatore	pain caused by heel blood sampling, changes in HR, change in oxygen saturation
2015	Quasi-Experimental Design	100	LBW; 32-36 w	KMC, half 1h for 4 times/day	stable	NA	2h/d	routine care	neonatal weight gain, physiological indices, number of breastfeeding
2020	RCT	80	31-35 w, > 1500 g	Mother/surrogate KC, 3 times of daily care once by mother and twice by surrogate in the last two time of care up to 5 days, 60 mins.	NA	Mother/surrogate	3h/d,5d	3 times of daily care by mother,5 days, 60 mins	nutritional behavior of preterm neonate, nutritional effect, HR, temperature, RR, arterial oxygen saturation
2015	RCT	120	37-42 w; 2500-4000 g;	Intervention 1: breast feeding. Intervention 2:KMC of neonate with close contact of thorax and abdomen skin of mother.	during vaccination	Mothers	0.2h	swaddling of neonate	pain score of BCG vaccine injection, pain score of less than 4 during BCG vaccine injection, duration of neonate crying during BCG vaccine injection
2015	RCT	106	2500-4000g; 37-42 w;	KMC and photo-therapy	during light treatment	Mothers	NA	only photo-therapy	neonatal serum bilirubin level, duration of hospitalization in neonatal unit, duration of phototherapy
2015	RCT	126	32-37 w	Time:15 mins before and 15 mins after invasive procedures. Intervention 1: KM Intervention 2: massage by mother	during procedure	Mothers	0.5h	supine position in incubator	pain, HR, oxygen saturation

Author/year	Study type	Sample	Participants	Intervention	Initiation	KMC giver	KMC Duration	Control	Outcomes
2015	RCT	116	37-40 weeks	KMC,30mins,3times/day	immediately	Mothers	1.5h	Routine care	the amount of bilirubin, mothers satisfaction from care after birth, the mothers experience of KMC
2017	RCT	100	28-34 w; >1200 g ,<2000 g	KMC,60 mins, twice/day in late morning and evening shifts, 1week	NA	Mothers	2h, 7d	Routine care	fatigue, depression
2019	RCT	90	32-36 w	24-48h after delivery, performed the method of KMC 10 days, 3times/day	early KMC, 24 - 48 hours after delivery	Mothers	10d	recommended care of preterm infants	postpartum depression
2021	Quasi-Experimental Design	72	<37 w	intermittent KMC the second day after birth,7 days and 1h/day	early KMC, the second day after birth	Mothers	1 h/d, 7d	Routine care	sleep quality of mothers with Preterm Infant
2018	RCT	90	37-42 w;2500-4000 g	Intervention 1: KMC,30-45min, ≥5 times/day in the morning, afternoon, and night.<br>Intervention 2: Field massage	NA	Mothers	2.5-3.75h/d	standard phototherapy and only breastfeed	neonatal blood bilirubin level

Author/year	Study type	Sample	Participants	Intervention	Initiation	KMC giver	KMC Duration	Control	Outcomes
2019	RCT	124	≤ 32 weeks;birth weight ≤ 1800g	KC Education based on the Expanded Health Belief Model	stable	Mothers	NA	routine hospital training	mother's perceived sensitivity score, mother's perceived intensity score, mother's perceived benefit score, The score of the perceived barriers of the mother, The score of the Cues to action of the mother, mother's self-efficacy score, mother's perceived social support score, mother's Locus of control score, weight of baby discharge time, The duration of oxygen therapy, duration of hospitalization, retinopathy, the death of babies, average baby's weight gain per day
2019	RCT	50	Weight ≥1000 g;<br>26 to 32 weeks	KMC,3times/day, 30-40 mins, continue to care for a month at home after discharge.	stable	Mothers	1.5-2h/d, 1m	KMC,3times/day,30-40 mins, routine care after discharge	resilience of mothers of premature infants, premature infants development
2021	RCT	50	Premature infants weight ≤1800 g,<br>30 w	each mother individually attends two sessions of 30 minutes in the conference room and is taught how to perform cuddle care using the baby model.KMC 3 times/day,30 mins .	NA	Mothers	1.5h/d	routine KMC training (pamphlet)	resilience, breastfeeding self-efficacy

Author/year	Study type	Sample	Participants	Intervention	Initiation	KMC giver	KMC Duration	Control	Outcomes
2021	RCT	50	≤1,800g,30 w	each mother individually attends two sessions of 30 minutes in the conference room and is taught how to perform cuddle care using the baby model. KMC 3 times/day,30 mins	NA	Mothers	1.5h/d	routine KMC training (pamphlet)	maternal-infant attachment, sleep quality of mothers
2020	RCT	66	28-37weeks;Birth weight ≥1500g	Intervention 1:KMC 30mins<br>Intervention 2: oral sucrose	during procedure(blood collection)	Mothers	0.5h	Routine care	pain score, RR, HR, percentage of arterial blood oxygen
2021	RCT	50	corrected age ≥35 weeks	SSC ≥30 mins (24 h/day), intermittent or continuous	NA	Mothers	intermittently, or continuous	Routine care	score of cornell assessment of pediatric delirium (CAPD) questionnaire
2023	RCT	80	34-37 weeks	Intervention1:tactile-kinetic stimulation<br>Intervention2: KMC, 3times/day,60 mins for 7 days	stable	Mothers	3h/d, 7d	NA	weight
2022	RCT	84	≥37 weeks;<br>> 2,500 g	Intervention 1:Massage<br>Intervention 2: KMC,30mins, ≥5times /day (every 3h) in the morning, evening and night shifts during breastfeeding.	NA	Mothers	>1.5h/d	phototherapy	bilirubin

Author/year	Study type	Sample	Participants	Intervention	Initiation	KMC giver	KMC Duration	Control	Outcomes
2022	RCT	80	32-36 weeks; weighing 1500-2500 g	KMC	NA	Mothers	NA	NA	vital signs
2024	Quasi-Experimental Design	47	<37 weeks weigh < 2500 g	KMC start once a day for 24 to 34 mins and gradually reach 1 to 3 hours three times/day	stable	Mothers	1-3h/d	without hugging	vital signs of premature infants
2023	RCT	85	corrected GA 30 to 35 weeks	Intervention1:KMC Intervention2:Vimala Massage Intervention3:KMC+Vimala Massage	stable	NA	NA	Routine care	weight, hospital stay
2016	RCT	266	<37 weeks <2500g	Community Health Volunteer-supported KMC	NA	Mothers	NA	NA	neonatal death, weight gain, breastfeeding
2018	RCT	160	28-36weeks	KMC	NA	NA	NA	Incubator care	pain
2024	RCT	176	28-36 weeks 1000g to <2500g	Immediate KMC	immediately	Mothers	NA	NA	reduced number of deaths, reduced risk of infections
2016	RCT	109	37-41 w	SSC	during vaccination	Mothers	NA	2 ml of glucose 25% oral solution	pain, crying time, adverse effects

Supplementary Figure S1

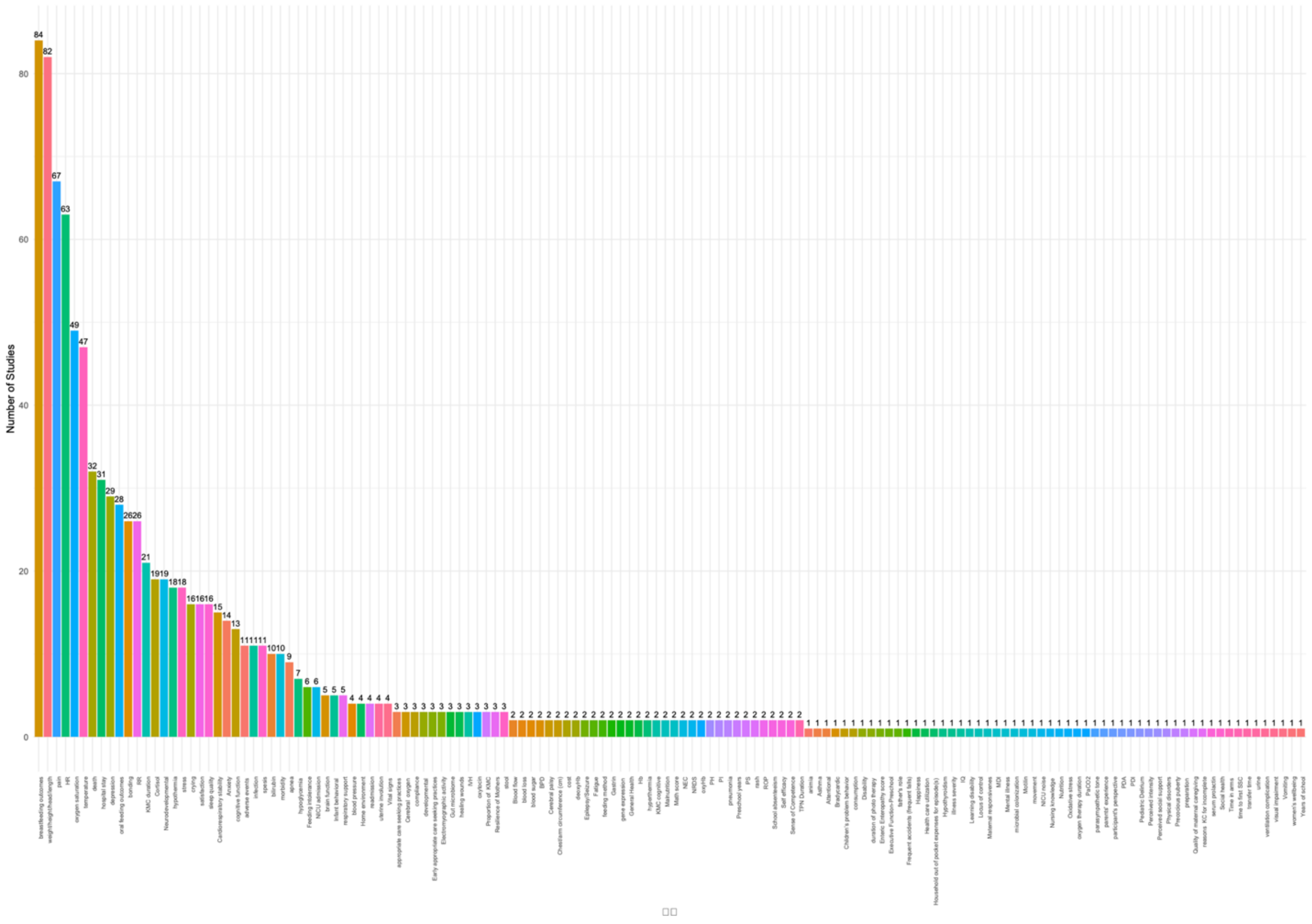

Explanation of difference in from the registered protocol and the manuscript

During the review process, certain deviations from the registered protocol were made to enhance the comprehensiveness and relevance of our search and analysis. These deviations are detailed as follows.

(1) Search Time: To incorporate the most recent publications, the search end date was extended from July 1, 2024, to July 7, 2024, following updates to available data during the study period. This minor adjustment did not significantly alter the results but enhanced the timeliness of the included studies.

(2) Databases: To improve literature coverage, we added CINAHL and SinoMed to the original database list (MEDLINE, Cochrane Library, Embase, and WanFang Data). These additions were made after initial exploratory searches revealed their relevance for capturing nursing-focused and Chinese medical literature on KMC, respectively, thereby ensuring a more comprehensive review.

(3) Quality Assessment: The primary aim of this review was to extract, analyze, and synthesize outcomes and their frequency of use to develop a core outcome set (COS) for KMC trials. Although our PROSPERO registration initially outlined plans to assess the quality of outcome reporting, we determined during the study that formal quality assessment of included studies or their outcome reporting was not necessary. This decision was informed by the observation that most systematic reviews of reported outcomes in KMC trials similarly omit quality assessments (e.g., [1-5]). Our focus was on comprehensively capturing the range of outcomes reported in KMC trials rather than evaluating treatment effects or methodological rigor. Omitting quality assessment streamlined the data extraction process and facilitated a more inclusive capture of outcomes. We have further clarified this rationale in the methods section (2.4 Quality Assessment) and discussed it in the strengths and limitations section (4.4) of the manuscript.

References

[1] Peng H, Shi J, Tang J, et al. Outcome reporting in neonatal septic shock studies: A systematic review. Aust Crit Care. 2025, 38(4):101227.

[2] Hall C, Shishkina A, Thurman R, et al. Outcome reporting in cardio-obstetrics studies: A systematic review. Am Heart J. 2024, 278:223-234.

[3] Wong C, van Oostrom J, Bossuyt P, et al. A Narrative Systematic Review and Categorisation of Outcomes in Inflammatory Bowel Disease to Inform a Core Outcome Set for Real-world Evidence. J Crohns Colitis. 2022, 16(10):1511-1522.
[4]Dadouch R, Faheim M, Susini O, et al. Variation in outcome reporting in studies on obesity in pregnancy-a systematic review. Clin Obes. 2019, 9:e12341.
[5]Villani LA, Pavalagantharajah S, D'Souza R. Variations in reported outcomes in studies on vasa previa: a systematic review. Am J Obstet Gynecol MFM. 2020, 2:100116.
